# CO_2_ Hydrogenation on Gas-Phase Palladium–Zinc Bimetallic Clusters

**DOI:** 10.1021/acsomega.5c05712

**Published:** 2025-09-19

**Authors:** Bárbara Zamora Yusti, Eszter Makkos, Ewald Janssens, László Nyulászi, Tibor Höltzl

**Affiliations:** a Department of Inorganic and Analytical Chemistry, 61810 Budapest University of Technology and Economics, Műegyetem rkp. 3., Budapest H1111, Hungary; b HUN-REN-BME Computation Driven Chemistry Research Group, 61810 Budapest University of Technology and Economics, Műegyetem rkp. 3., Budapest H1111, Hungary; c Quantum Solid-State Physics, Department of Physics and Astronomy, KU Leuven, Celestijnenlaan 200D, Leuven B3001, Belgium; d Nanomaterials Science Group, Furukawa Electric Institute of Technology, Késmárk utca 28/A, Budapest H1158, Hungary

## Abstract

Hydrogenation of CO_2_ to valuable products is an attractive method to mitigate the greenhouse effect. Palladium–zinc-based nanomaterials are stable and selective catalysts in the methanol formation process. For a better understanding of the catalytic reaction mechanisms, here, we investigate hydrogen activation and carbon dioxide hydrogenation on Pd_*x*_Zn_*x*_ (*x* = 2–4) and Pd_6_ clusters using systematic density functional theory analysis, selected on the basis of high-level benchmarks. We show that alloying palladium with zinc makes the H_2_ adsorption and dissociation less favored and increases the energies of the highest lying transition states, while zinc strongly stabilizes the formate intermediate, by binding one of its oxygen atoms through a (partial) ionic bond.

## Graphical abstract

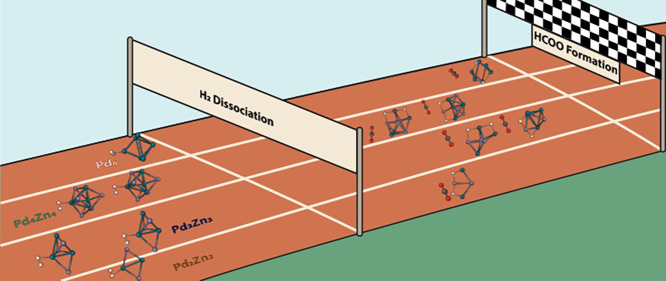

## Introduction

One possible solution to mitigate the effects of CO_2_ emissions in the atmosphere is to capture this greenhouse gas and utilize it as a feedstock for valuable chemicals,^1^ thereby promoting a circular economy based on Power-to-X technologies. An attractive method for this conversion is the thermal hydrogenation of CO_2_, using hydrogen from renewable sources. A highly desired product is methanol, which is easy to store and transport, and is compatible with the established processes of the chemical industry.^2^ Although the CO_2_ hydrogenation reaction toward methanol (eq 1.) is thermodynamically favored, catalysts are necessary to reduce the energy barriers involved. Copper-based catalysts, such as the well-known Cu/ZnO, are commonly used for this process.^3^ However, along with the desired CO_2_ hydrogenation to methanol, competing reactions, such as the reverse water gas shift (RWGS) reaction (eq 2), are also feasible. Accordingly, there is a significant demand to enhance both the activity and selectivity of the CO_2_ hydrogenation to methanol, prompting a search for better catalysts.

$${\mathrm{CO}}_{2}+3H_{2}\to {\mathrm{CH}}_{3}\mathrm{OH}+H_{2}O$$ 1

$${\mathrm{CO}}_{2}+H_{2}\to \mathrm{CO}+H_{2}O$$ 2

$${\mathrm{CH}}_{3}\mathrm{OH}+H_{2}O\to {\mathrm{CO}}_{2}+3H_{2}$$ 3

Consequently, a number of metals and their alloys are undergoing intensive investigation.^4^ Palladium-based catalysts are well-known for their activity in redox reactions.^5–7^ Pd/ZnO was also found to effectively catalyze the CO oxidation, where strong metal–support interaction and alloying has been observed.^8^ Recently, these catalysts are gaining attention for CO_2_ conversion due to their ability for fine-tuning the selectivity toward the desired methanol product, with the additional benefit of their exceptional resistance to sintering.^9^ It has been shown that depending on the microstructure, composition, and reaction conditions, palladium–zinc based catalysts are active in CO_2_ hydrogenation toward methanol (eq 1),^10–17^ reverse water gas shift reaction^18,19^ (eq 2), or methanol steam reforming reactions (eq 3, i.e. the reverse of CO_2_ hydrogenation).^20–28^ An interesting feature of Pd based catalysts is the condition dependent ability of atomic dispersion of the metal, and the reagglomeration into particles, which contributes to the sustained activity.^5^ It is known that also the support (CeO_2_,^29^ TiO_2_,^30–32^ ZrO_2_,^33^ SiO_2_,^32,34^ Al_2_O_3_,^31,32,34^ and Ga_2_O_3_ ^35,36^ ) has a significant influence on the catalytic activity.

Identification of the active phase and active sites, as well as the understanding of the reaction mechanisms are crucial for catalyst development based on atomistic design.^37–39^ However, due to the complex structures of these catalysts, which can even change under operando conditions, their reactivity remains the focus of intensive research. Oxide-supported metal catalysts present additional challenges due to the possible strong metal–support interactions.^40^ In these systems, not only the metal particle and the oxide support, as well as their interface, have different roles in the catalytic activity, furthermore, even alloys may form in a reducing hydrogen atmosphere.

Among the different palladium-based catalysts used for methanol synthesis, Pd/ZnO systems are particularly important, as they offer a clear separation of the possible competing reactions, leading preferentially to methanol.^11,12,17^ The mechanism of the PdZn and Pd/ZnO-catalyzed hydrogenation is the subject of intensive research. It has been observed experimentally that PdZn alloy, preferentially in a 1:1 metal ratio, and PdH_*x*_ hydrides can form during the reaction.^11,12,17^ Zinc was found to have a critical role in the methanol formation, as pure palladium, in the absence of zinc, behaves as a RWGS catalyst.^11^ It has been proposed that the PdZn phase is responsible for the RWGS reaction, while the active sites for the methanol synthesis are located at the Pd/ZnO interface.^17^

The CO_2_ hydrogenation mechanism has been computationally investigated on transition metal surfaces, including Pd (111).^41^ It has been shown that direct CO_2_ dissociation has a higher barrier than hydrogenation, and the hydrogenation preferentially follows the carboxylate route, while the formate pathway is only slightly higher in energy.^41^ Carbon monoxide is an important intermediate, which preferentially hydrogenates first on its carbon atom. Methane formation was found to exhibit higher barriers than the routes leading to methanol.^41^ The reaction mechanisms on Pd(111) and PdZn(111) alloys were compared, and it was concluded that zinc weakens the bond with carbon atoms of the adsorbates,^42^ while the oxygen–metal bonds are strengthened. These results explain the preference of the formate pathway compared to carboxylate on the alloys. The binding energy change of intermediates in the water gas shift reaction is related to the different electron-donation and electron-accepting properties of zinc and palladium.^43,44^

The Pd and PdZn, either as nanoalloys or core–shell nanoparticles, catalyzed CO_2_ hydrogenation reaction mechanism to methanol has been investigated using Density Functional Theory (DFT) based methods.^45^ It has been concluded that zinc lowers the rate-determining reaction barrier and that the Pd_32_Zn_6_ nanoalloy is more active than the Pd_32_Zn_6_ core–shell nanoparticle. However, only three cluster sizes were examined, without systematically investigating the stability of the catalysts or the size effects.

Metal clusters are active catalysts in several processes, and interestingly, they can be formed dynamically at operando conditions. Size-selected small metal clusters can be synthesized in the gas phase, and their reactions with various gases can be investigated.^46^ Gas phase metal clusters are idealized model systems for heterogeneous catalysts that allow to systematically study the effect of the composition, size and charge state on the reaction under well-defined conditions.^47^ In a combined experimental-computational investigation of metal clusters, the CO oxidation reaction mechanism on small palladium clusters was found to be similar to that on palladium surfaces, while the CO desorption is less hindered on the clusters.^48^ Clusters can also be deposited on surfaces,^49^ where they can be highly active catalysts for various processes, including CO_2_ hydrogenation, and can be model systems to investigate the role of the support. CO_2_ hydrogenation to formate/carboxylate has been investigated computationally using ZrO_2_ supported subnanometer palladium–zinc model clusters.^33^

In this work, we conduct a detailed computational investigation of CO_2_ hydrogenation over three bimetallic clusters—Pd_2_Zn_2_, Pd_3_Zn_3_, and Pd_4_Zn_4_—that feature the preferred 1:1 Pd:Zn ratio. We selected these three specific cluster sizes to facilitate a systematic yet computationally feasible comparison of the reaction pathways. Pd_6_ was chosen as the monometallic reference, while Pd_3_Zn_3_ serving as a directly comparable bimetallic counterpart with the same number of atoms. The smaller (Pd_2_Zn_2_) and larger (Pd_4_Zn_4_) clusters were included to examine size effects across the series. Additionally, these cluster sizes are relevant for experimental techniques such as IRMPD spectroscopy, which typically probes similar size ranges. Two mechanistic pathways are explored: one initiated by H_2_ dissociation followed by CO_2_ adsorption, and the other starting from CO_2_ coordination in the presence of intact H_2_. Both routes are assessed in terms of their ability to yield key intermediates, Pd_*x*_Zn_*x*_HCOO (formate pathway) and Pd_*x*_Zn_*x*_COOH (carboxylate pathway). For comparison, the reactions are also studied on the pure Pd_6_ cluster to gather information on the effect of Zn in the absence of any oxide interface.

## Methods of Computation

The structural search for the neutral Pd_6_, Pd_2_Zn_2_, Pd_3_Zn_3_ and Pd_4_Zn_4_ clusters was carried out using the CALYPSO^50^ code in conjunction with the Gaussian 16 ^51^ quantum chemical program. Two local optimizations at the BP86/LANL2DZ level of theory, with increasingly tight convergence criteria were, performed consecutively for each cluster. The preoptimization employed loose convergence criteria (maximum step size of 0.0100 au, RMS force of 0.0017 au and the SG1 (50,194) integration grid, while tighter criteria were used for the final optimization (maximum step size of 0.0018 au, RMS force of 0.0012 au and a default ultrafine (99,590) integration grid). Subsequently, the obtained isomers were reoptimized at the PBE0-D3/def2-TZVP level of theory using the Q-Chem software.^52^ Analytical second derivatives of the molecular energy with respect to the nuclear coordinates were computed to confirm that each located structure had no imaginary vibrational frequency, i.e. minima are located. Binding modes for the reaction with CO_2_ and H_2_ molecules were systematically evaluated using an in-house code that has been applied previously.^53,54^

The PBE0-D3 functional and the def2-TZVP basis set have been selected based on extensive benchmarking employing the CCSDT­(Q, FC)/aug-cc-pVTZ-PP method with correction for semicore–valence correlation using the CCSD­(T, FC/Full)/aug-cc-pwCVQZ-PP for PdH, ZnH, Pd_2_, PdH_2_, PdCO_2_, while CCSD­(T, FC)/def2-TZVPPD was used as reference for binding energies of different adducts on Pd_*x*_Zn_*x*_ (x = 2–4) clusters. It is worth noting that the PBE0 functional previously has been applied to investigate hydrogen adsorption and dissociation on small palladium clusters.^55,56^

To explore the reaction pathways for the CO_2_ hydrogenation and H_2_ dissociation reactions, intermediates and products were located for all the investigated clusters. Initial geometries of the transition structures (TSs) and approximate reaction paths between the different binding modes were searched for using the Freezing String Method (FSM).^57,58^ The initial guess geometry of the transition structure was further refined using the eigenvector-following method, as implemented in the Q-Chem software.^52^ The computed TS structures exhibited one and only one imaginary frequency. Once the transition state geometry was located, Intrinsic Reaction Coordinate (IRC) computations were performed to verify the proposed energy pathway. Reaction energies are computed with respect to the separated bare clusters and isolated H_2_ and CO_2_ molecules.

The electronic structures of the most stable clusters were studied using the Multiwfn^59^ package for topological analysis with Bader’s quantum theory of atoms in molecules (QTAIM), as electron densities at the bond critical points are known to be good descriptors of atomic interactions.^60–62^ Localized molecular orbitals (LMOs) were computed using the Pipek-Mezey localization based on Becke populations method.^63^ The Natural Bond Orbitals (NBO)^64^ package, incorporated into Q-Chem, was used for Natural Population Analysis to investigate the atomic charges. Projected density of states was calculated for the bare clusters using the Hirshfeld method as implemented in the Multiwfn package.^59^

Gibbs free energies were computed using the Rigid Rotor Harmonic Oscillator approximation, to reveal qualitative trends across clusters rather than to provide quantitatively precise thermodynamic values.

## Results and Discussions

We have carried out an extensive search to identify the most stable bare clusters, potential intermediates and products of the reactions, and the favored reaction paths. We first analyze the geometric and electronic structures of the putative global minima of the Pd_6_, Pd_*x*_Zn_*x*_ (x = 2–4) clusters. Then, we discuss hydrogen adsorption and dissociation on these lowest energy clusters, and finally, we present several possible CO_2_ hydrogenation reaction pathways leading to formate or carboxylate intermediates.

### Gas Phase Cluster Structures

Previous studies have modeled the pure Pd_6_ cluster in both its singlet and triplet states.^65,66^ In agreement with previous results, we found the triplet multiplicity to be more stable than the singlet by 45 kJ/mol. Therefore, the triplet was selected for further investigation. On the other hand, all low-energy isomers of the Pd_*x*_Zn_*x*_ bimetallic clusters adopt a singlet state ground state, with corresponding triplet states lying at least 100 kJ/mol higher in energy than the lowest-energy singlet.

The global minimum for Pd_6_ adopts a quasi-octahedral structure (Figure 1), with a pentagonal pyramid structure located 88 kJ/mol higher in energy. Computed Pd–Pd distances in Pd_6_ are between 2.542 Å and 2.804 Å, while the natural charges of the Pd atoms span the range between −0.06 and 0.01 au Our findings are in line with the previously reported bond lengths of 2.70 Å by Li et al.^67^ and are slightly below the 2.75 Å obtained by Bertani et al.^66^ who reported a distorted octahedron with bond lengths closer to those of bulk Pd (2.75 Å).^68^ Additionally, the presence of low-frequency vibrational modes with Pd–Pd stretching character indicates that the Pd–Pd bond lengths in Pd_6_ are quite flexible.

**Figure 1:**
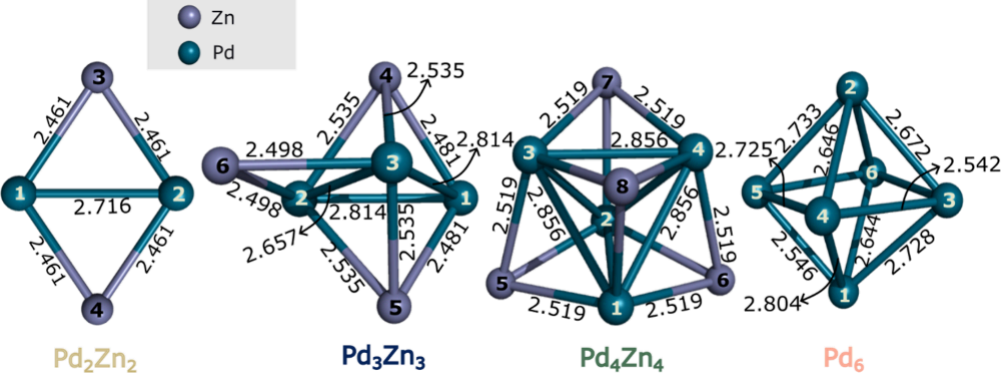
Left to right: global minima of Pd_2_Zn_2_, Pd_3_Zn_3_, Pd_4_Zn_4_ and Pd_6_ clusters computed using the PBE0-D3/def2-TZVP level of theory. Palladium atoms are represented by dark cyan spheres, while zinc atoms are shown as lavender colored spheres. Bond lengths are indicated in Å. Color coding follows the batlow sequential palette,^69^ used consistently across all figures to match chemical formulas and reaction pathways.

To the best of our knowledge, the structures of 1:1 PdZn bimetallic clusters containing 4, 6, or 8 atoms have not been reported in literature. All these mixed clusters have singlet ground state. The Pd_2_Zn_2_ cluster features a symmetrical planar structure in which the palladium atoms are separated by 2.716 Å and bound to both zinc atoms with an equal bond length of 2.461 Å. The structural search identified Pd_3_Zn_3_ as a trigonal bipyramid with an additional zinc attached in bridge position as the global minimum (Figure 1), while the second most stable isomer was found to exhibit a distorted pentagonal pyramid structure, lying 47 kJ/mol above the putative global minimum. The geometrical analysis summarized in Figure 1 reveals three Pd–Pd bonds, one with a somewhat shorter length of 2.657 Å at the bridging Zn, and two with significantly longer Pd–Pd distances of 2.814 Å. The Pd–Zn distances are either 2.481, 2.498 Å or 2.535 Å. Natural atomic charges indicate that Pd atoms carry negative charges (−0.32 and −0.34 au for higher and lower coordinated Pd, respectively), while the Zn atoms are positively charged (0.36 au for the highly coordinated 4 and 5 Zn atoms, and 0.26 a.u, for the lower coordinated Zn atom 6, Figure 1). The global minimum of Pd_4_Zn_4_ has a Pd_4_ tetrahedral core which is capped by four Zn atoms, resulting in a highly symmetrical structure. Pd–Pd distances in the tetrahedral core are all 2.856 Å, while Pd–Zn distances on the caps are 2.519 Å. Similarly to Pd_3_Zn_3_, the palladium atoms carry a uniform negative charge, whereas zinc atoms carry a uniform positive charge of 0.44 au However, it should be noted that there are four low-energy isomers within an energy range of 39 kJ/mol above the global minimum (see Table S8 in the Supporting Information).

Notably, in the bimetallic Pd_*x*_Zn_*x*_ clusters, zinc tends to occupy a lower-coordinated position than palladium. The distances between the zinc atoms are large, which corresponds to the weak Zn–Zn interactions. Interestingly, the average Pd–Pd bond length increases from 2.716 Å in Pd_2_Zn_2_ to 2.762 Å in Pd_3_Zn_3_ and 2.856 Å in Pd_4_Zn_4_. Similarly, the average Pd–Zn bond length increases from 2.461 Å to 2.512 Å and 2.586 Å, respectively. Overall, the most stable bimetallic Pd_*x*_Zn_*x*_ clusters have elongated Pd–Pd distances compared to the pure Pd_6_ cluster (or bulk Pd), indicating a potential fluxionality of the mixed Pd–Zn clusters. Interestingly, Pd–Zn bond lengths are shorter than in the 1:1 PdZn bulk alloy (2.64 Å).^68^

The projected density of states (PDOS) results from Figure S9 and Table S15 show that the Pd *d*-band centers in Pd_*x*_Zn_*x*_ clusters move progressively closer to the Fermi level (approximated as the HOMO energy) as Zn content increases (e.g., + 1.30 eV in Pd_4_Zn_4_ compared to. + 2.58 eV in Pd_2_Zn_2_).

### H_2_ Adsorption and Dissociation

The hydrogenation of metal clusters was studied by evaluating both molecularly adsorbed ″intact″ hydrogen (H_2_), and dissociated hydrogen binding modes. Figure 2 summarizes the possible isomers for both types of hydrogen adducts up to 50 kJ/mol relative energy compared to the lowest-energy structure. Metal clusters are well-known for their potential fluxional behavior.^70,71^ It has been shown that the availability of several energetically accessible binding sites can lead to an ensemble of structures^72^ which collectively determine their catalytic activities, as it has been shown for platinum-hydride clusters in the hydrogen evolution reaction.^73^ This observation aligns with our previous findings of a complex multistep reaction mechanism for the CO_2_ activation and dissociation on small Pd_*x*_Pt_4‑x_ clusters.^54^ Thus, we conducted an ensemble analysis of the obtained low-lying hydrogen adduct isomers to predict the reactivity. This ensemble behavior can be interpreted using a Boltzmann-weighted population model, where low-energy isomers contribute most significantly to the observed reactivity. Such approaches have been applied by the Alexandrova group.^70,72,73^

**Figure 2:**
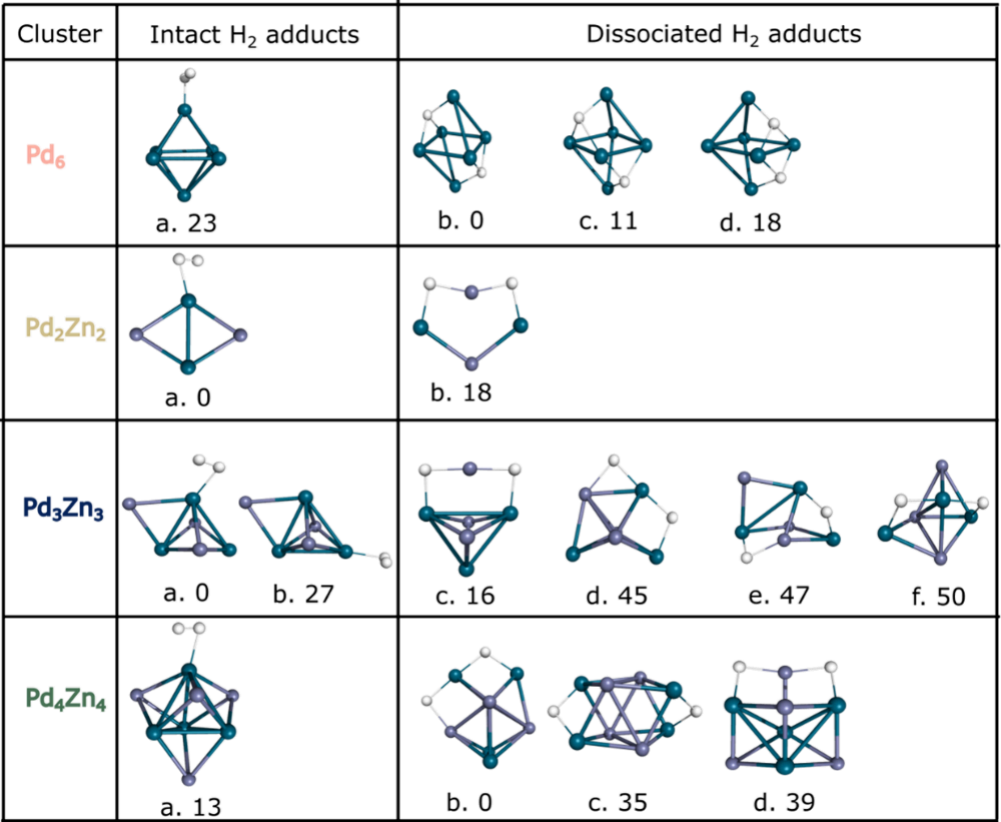
Geometries of the most stable H_2_ adducts of Pd_6_ and Pd_*x*_Zn_*x*_ (x = 2–4). Relative energies (calculated at the PBE0-D3/def2-TZVP level) are compared to the lowest energy isomers and indicated in kJ/mol. Palladium atoms are represented by dark cyan spheres, while zinc atoms are shown as lavender colored spheres.

The quasi-octahedral shape of Pd_6_ naturally leads to several symmetrically equivalent isomers for both the intact and the dissociated hydrogen binding, which, through a symmetry factor in the reactive collisions, will affect the reactivity. As such, there are six possible intact H_2_ adducts of Pd_6_ (Figure[Fig fig2] Pd6/a), while the possible isomers with dissociated hydrogen adducts is much larger (Figure 2 Pd_6_/b, /c and/d). This implies an important entropy effect, which further can stabilize the dissociated hydrogen adducts.

Due to its small size, the Pd_2_Zn_2_ cluster has only a few possible coordination possibilities with H_2_, of which only two exhibit a low relative energy: one intact (Pd_2_Zn_2_/a) and one dissociated adduct (Pd_2_Zn_2_/b). In contrast, the Pd_3_Zn_3_ palladium–zinc cluster behaves differently. As not all palladium sites are equivalent, there is a notable preference for H_2_ binding to specific palladium atoms. H_2_ prefers to bind to either of the two symmetry equivalent palladium atoms connected by a bridge-coordinated Zn (Figure 2 Pd_3_Zn_3_/a), with a preference of 27 kJ/mol compared to binding to the third palladium atom (Figure 2 Pd_3_Zn_3_/b). A similar preference is observed in case of dissociated hydrogen, where the structure shown in Figure 2 Pd_3_Zn_3_/c is 29 kJ/mol more stable than Pd_3_Zn_3_/d. Interestingly, the relative energies of the structures with dissociatively bound hydrogen increase further when the H atoms are bound to palladium instead of to zinc. The Pd_4_Zn_4_ cluster has a highly symmetrical structure with four equivalent palladium sites for intact H_2_ binding. In contrast to the smaller PdZn clusters, the lowest energy Pd_4_Zn_4_H_2_ isomer has dissociated hydrogen, with the H atoms bound mainly to palladium atoms (Figure 2 Pd_4_Zn_4_/b). Other nonsymmetry equivalent isomers (Figure 2 Pd_4_Zn_4_/c, /d) lie at least 35 kJ/mol higher in energy.

Overall, these results show that the zinc alloying of the palladium clusters leads to a significant stabilization of a few particular isomers with specific binding modes and positions of the hydrogen, and with a relatively low symmetry. Thus, the alloying of palladium with zinc is expected to reduce the ensemble effect in the H_2_ binding and dissociation.

After establishing the energetically most stable H_2_ adducts of the studied clusters, a topological analysis was conducted to further investigate the effects of the Zn atoms and cluster size on the hydrogenation process.

Intact H_2_ prefers to bind to a single palladium atom in η^2^ coordination. This can likely be attributed to Kubas interaction,^74^ as the partially filled *d* orbitals of Pd can participate in donation-back-donation mechanism with H_2_. The molecular form of hydrogen is reflected in the negative Laplacian values of the H–H bond critical points (BCP) (see Table 1), which are typically observed for covalent bonds. Orbital localization analysis further supports this statement by revealing a three-centered localized molecular orbital for each studied cluster, comprising the hydrogen atoms of the molecule and the coordinating Pd atom. Comparing the different studied clusters, the H_2_ binding strength decreases in the following order: Pd_3_Zn_3_ > Pd_6_ ≈ Pd_2_Zn_2_ ≫ Pd_4_Zn_4_. Generally, shorter Pd–H bond lengths and higher electron densities at the corresponding BCPs (along with lower electron densities at the H–H BCP and longer H–H bonds) indicate stronger interaction between the H_2_ molecule and the metal cluster. The underlying assumption is that these geometrical and electronic descriptors directly correlate with the binding strength. Interestingly, while this assumption is valid for the Pd_*x*_Zn_*x*_ bimetallic clusters, where the energetic order based on the binding energies aligns with the expected interaction strengths derived from the geometrical parameters, Pd_6_ stands out in this respect. While the binding energy of H_2_ on Pd_6_ is similar to that on Pd_2_Zn_2_, the Pd–H_2_ distance is the shortest and the H–H bond is the longest among the (nondissociated) H_2_ adducts of the studied clusters. This behavior might be related to the fact that Pd_6_ and its corresponding adduct have a triplet state, while the bimetallic PdZn clusters have a singlet ground state. By examining the difference in bond lengths between the hydrogenated and bare clusters, we found that the bonds associated with the coordinating Pd atom are significantly elongated within the Pd_6_ cluster (total bond length deviation (∑(*d*_*Pd*―*X*_^*adduct*^-*d*_*Pd*―*X*_^*cluster*^) of ∑0.348 Å). The sum of the bond length elongation is small for the Pd_2_Zn_2_ and Pd_3_Zn_3_ clusters, but it is again considerable (∑0.202 Å) for Pd_4_Zn_4_, as shown in Table 1. The changes in the geometry of the clusters indicate activation, which might result in a reduction of the energy barriers in subsequent reaction steps.

**Table 1:** Binding Energies (BE), Bond Lengths (d), Electron Densities at the Bond Critical Points BCPs (ρ_BCP_) and Their Laplacian (∇^2^ρ) for the η^2^-H_2_ Bond in the Intactly Bound H_2_ Complexes of the Studied Clusters^a^

	Pd_6_ – H_2_		Pd_2_Zn_2_–H_2_		Pd_3_Zn_3_–H_2_		Pd_4_Zn_4_–H_2_	
	Pd1–H_2_	H7–H8	Pd1–H_2_	H5–H6	Pd2–H_2_	H7–H8	Pd1–H_2_	H9–H10
BE (kJ/mol)	–69		–68		–76		–50	
*d*(Å)	1.695	0.859	1.734	0.835	1.729	0.841	1.764	0.829
ρ_BCP_(a.u.)	0.111	0.202	0.102	0.212	0.103	0.209	0.097	0.213
∇^2^ρ(a.u.)	0.366	–0.606	0.369	–0.687	0.368	–0.670	0.351	–0.702
∑(*d*_*Pd*―*X*_^*adduct*^-*d*_*Pd*―*X*_^*cluster*^) (Å)	0.348		0.051		0.079		0.202	
	Pd_6_(2H)		Pd_2_Zn_2_(2H)		Pd_3_Zn_3_(2H)		Pd_4_Zn_4_(2H)	
BE (kJ/mol)	–93		–50		–60		–63	

a ∑(*d*_*Pd*―*X*_^*adduct*^-*d*_*Pd*―*X*_^*cluster*^) represents the sum of all bond length differences related to coordinating Pd atom between the intact H_2_ adduct and the bare metal cluster

In line with previous results for palladium clusters of comparable sizes,^55^ dissociated H_2_ is energetically favored over molecular hydrogen on Pd_6_ (Figure 2). In Pd_6_(2H), there are six Pd–Pd bonds with an average length of 2.730 Å and six Pd–H bonds, as each hydrogen atom binds to three different palladium atoms, with an average bond length of 1.766 Å. The hydrogen atoms are slightly negatively charged (−0.22 au), suggesting a partially ionic palladium-hydride formation. This structure is basically the same as the most stable structure identified by Li et al., where 2.74 Å (Pd–Pd) and 1.74 Å (Pd–H) distances were found.^67^

While for Pd_6_ the adduct with dissociated hydrogen is clearly stabilized over the one with molecular hydrogen, alloying palladium with zinc stabilizes the binding with molecular hydrogen as was mentioned above. For Pd_2_Zn_2_ and Pd_3_Zn_3_ the relative stability is even reversed (Table 1). Interestingly, while intact H_2_ is always coordinated to a single palladium atom, in the dissociated hydrogen adducts of Pd_2_Zn_2_ and Pd_3_Zn_3_, both hydrogen atoms bind symmetrically to the same Zn atom in a quasi-linear structure, resembling to a ZnH_2_ molecule. Based on the QTAIM analysis, the involved Zn atom binds only to hydrogens, not to metal atoms, therefore this unit is indeed “detached” from the original cluster. The natural charges on the atoms further support the interpretation of the Pd_2_Zn_2_(2H) and Pd_3_Zn_3_(2H) clusters as composed of two distinct subunits: ZnH_2_ and Pd_2_Zn or Pd_3_Zn_2_, respectively. In both clusters, the two hydrogen atoms carry equal charges of – 0.43 au, while the Zn atoms bear charges of +1.01 and +1.06 au in Pd_2_Zn_2_ and Pd_3_Zn_3_, respectively. This charge distribution reinforces the idea that the ZnH_2_ moiety is connected to the Pd–Zn framework through two Pd–H bonds.

For the Pd_3_Zn_3_ cluster, we examined whether alternative dissociated hydrogen adduct isomers, in which both hydrogen atoms bridge only between Pd atoms, would be more stable (see Figure S3). However, that did not seem to be the case, confirming that the ZnH_2_–Pd_3_Zn_2_ structure is the most stable arrangement for hydrogenated Pd_3_Zn_3_(2H) clusters.

A similar loosely connected ZnH_2_ unit was not found in case of the most stable Pd_4_Zn_4_(2H) cluster, which has an asymmetric structure with one hydrogen atom bound to two Pd atoms with 1.722–1.734 Å bond lengths and the other to one Pd (1.722 Å) and one Zn atom (1.778 Å). This asymmetry is also reflected in the atomic charges (−0.27 and −0.47 au) and the three-centered localized molecular orbitals containing the above-described atoms (Figure S2). Although they are structurally distinct, the binding energies of Pd_3_Zn_3_(2H) and Pd_4_Zn_4_(2H) are lying close, at −60 and −63 kJ/mol, respectively.

As it is shown above, the most stable H_2_ adduct is the intact form for Pd_2_Zn_2_ and Pd_3_Zn_3_ (Figure 2), while the dissociated form is preferred for Pd_4_Zn_4_. The binding energies presented, along with the geometrical analysis (Table 1), suggest that this relative energy difference is closely related to the structural changes within the clusters: the formation of ZnH_2_ and the subsequent detachment of Zn atoms in the smaller Pd_*x*_Zn_*x*_ clusters, along with the relatively small binding energy of the Pd_4_Zn_4_–H_2_ cluster, both contribute to this difference.

Figure 3 shows the calculated energy barriers between the most stable molecular and dissociated hydrogen adducts of each studied cluster. Pd_6_ presents the lowest energy barrier of 42 kJ/mol for the rate-determining step from molecularly bound H_2_ to dissociated H_2_. This is consistent with the observation that the longest H–H distance among the H_2_ adducts occurs in Pd_6_, indicating the largest activation of the H–H bond. The Pd_6_–H_2_ cluster has a triplet ground state, and we found that the rate determining transition state has the same multiplicity, while the singlet state is 51 kJ/mol higher in energy. This difference in barriers for spin multiplicities has been noted for H_2_ dissociative adsorption on Pd_N_ (N = 2–4, 7, 13, 19, and 55) clusters, while no change in the ground state spin multiplicity is found after H_2_ molecular adsorption.^55^ Pd_6_ undergoes two additional reaction steps with 2 and 5 kJ/mol energy barriers, during which the hydrogen atoms migrate to their most preferred positions, where they are located on two opposite faces in the distorted octahedral structure in a μ_3_ binding mode. Similar results were obtained for the dihydride complex structures of Pd_6_(2H)^67^ and Pd_*x*_ (x = 2–4, 7, 13, 19, and 55)^55^ clusters, with dissociated H atoms preferentially bound on 3-fold faces.

**Figure 3:**
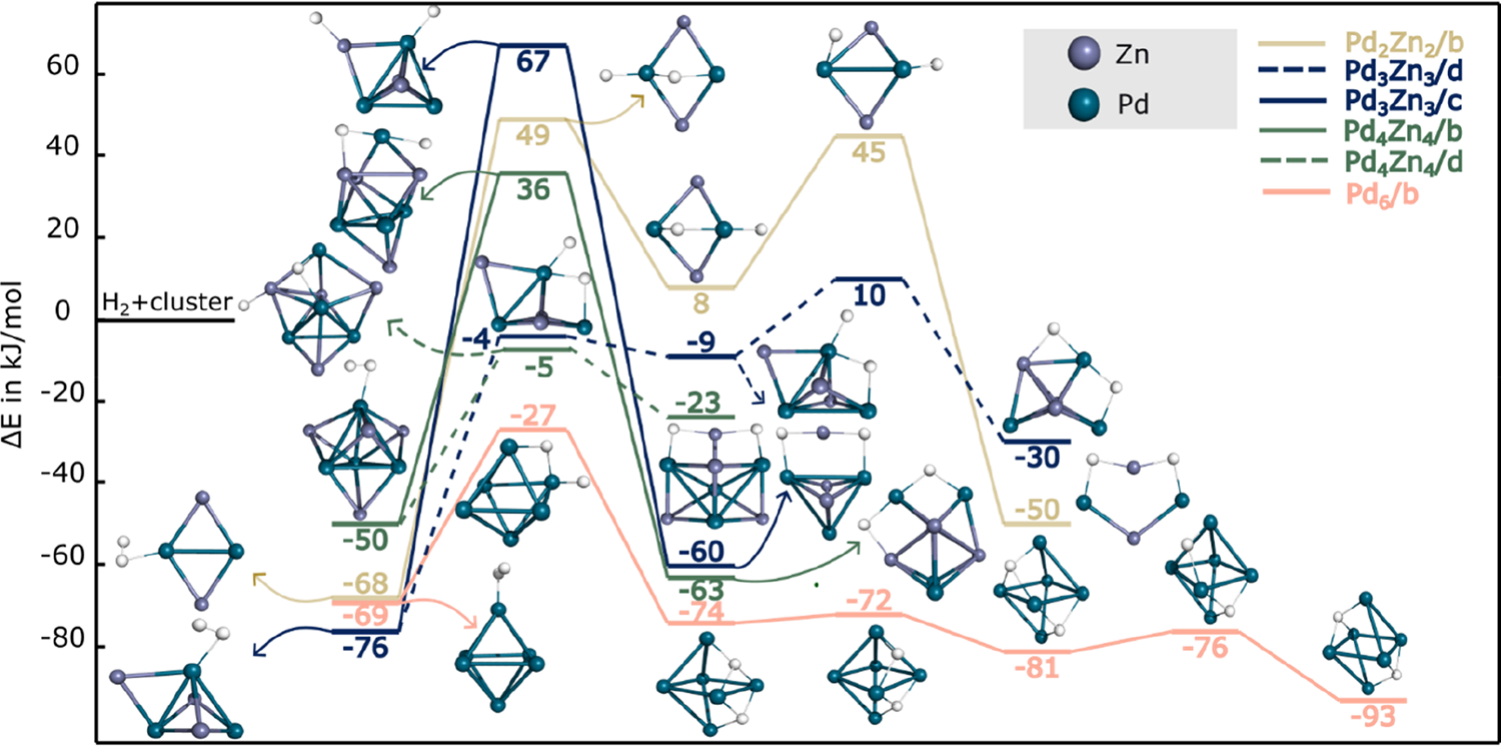
Reaction paths for the dissociation of hydrogen on Pd_6_, Pd_2_Zn_2_, Pd_3_Zn_3_, and Pd_4_Zn_4_. For Pd_3_Zn_3_ and Pd_4_Zn_4_, two representative paths are shown: one connecting the most stable intact and dissociated H_2_ adducts, and another involving a less stable dissociated structure with a significantly lower activation barrier (as discussed in the main text). H, Pd, and Zn atoms are depicted as white, cyan, and lavender colored spheres, respectively.

Among the Pd_*x*_Zn_*x*_ bimetallic clusters, H_2_ splitting on Pd_4_Zn_4_ has the smallest energy barrier of 86 kJ/mol, in accordance with the fact that the H_2_ adduct of this cluster showed the smallest stabilization among the investigated ones (Table 1). The initial structure of Pd_4_Zn_4_, which features a Pd_4_ tetrahedral core capped by four Zn atoms, becomes distorted causing each hydrogen atom to bridge Pd–Zn and Pd–Pd bonds, respectively. Hydrogen dissociation on smaller clusters, Pd_2_Zn_2_–H_2_ and Pd_3_Zn_3_–H_2_, has significantly larger energy barriers: 117 and 143 kJ/mol. Clearly, the original cluster structure was not significantly influenced by the incoming H_2_ upon adduct formation, while the formed product with the dissociated hydrogen results in a significantly different bonding (detached ZnH_2_) as was discussed above. In the reaction path Pd_2_Zn_2_–H_2_ first forms a high-energy intermediate structure in which one of the hydrogen atoms is bridged between the two Pd atoms of the cluster, then undergoes a second reaction step with a smaller 37 kJ/mol energy barrier to form the final product of Pd_2_Zn_2_(2H), while H_2_ splits and ZnH_2_–Pd_3_Zn_2_ forms (the most stable dissociated hydrogen adduct on Pd_3_Zn_3_) via a one-step mechanism (Pd_3_Zn_3_/c in Figure 3). In the case of Pd_3_Zn_3_ and Pd_4_Zn_4_, an alternative reaction pathway between the most stable intact hydrogen adduct and the second (or third, in case of Pd_4_Zn_4_) most stable dissociated adduct exhibits a significantly lower rate-determining energy barrier of 72 kJ/mol for Pd_3_Zn_3_/d and 45 kJ/mol for the Pd_4_Zn_4_/d route, respectively. They are also shown in Figure 3. This finding suggests that, although the final products in this steps are thermodynamically not the most stable ones (at −30 and −23 kJ/mol, respectively), the moderate activation energy allows their formation. Therefore, these adducts cannot be excluded as viable intermediates within the overall route.

The calculated reaction pathways indicate that molecular hydrogen binding is thermodynamically stable for both the Pd_6_ and for the palladium–zinc bimetallic clusters studied here. The dissociation of dihydrogen on the clusters is thermodynamically favored only for Pd_6_ and Pd_4_Zn_4_, and the formation of these structures is feasible since the corresponding barriers are low (42 kJ/mol) and moderate (86 kJ/mol), respectively. For Pd_2_Zn_2_ and Pd_3_Zn_3_/c, the high barriers leading to thermodynamically unfavored activated hydrogen adducts indicate that the involvement of such Zn containing species is unlikely in further reactions. Nonetheless, in the case of Pd_3_Zn_3__I the formation of a rather high-energy activated hydrogen adduct (at −30 kJ/mol in Figure 3) is kinetically possible.

According to the Hammer–Nørskov model,^75^ a shift of the *d*-band center toward the HOMO energy affects the activity of the catalyst and leads to a stronger chemisorption. Notably, we observe a strong linear correlation (R^2^ = 0.97, Figure S10) between the adsorption energies of the PdZn­(2H) adducts and the Pd *d*-band centers reported in Table S15. This trend suggests that dissociatively adsorbed H_2_ binds more strongly to Zn-rich clusters. By contrast, the Pd *d*-band center did not show a clear correlation with the adsorption energy of intact H_2_, the H_2_ dissociation barrier, or the CO_2_ binding energies of the bimetallic clusters.

To assess the isotopic sensitivity of the H_2_ activation step, we estimated the kinetic isotope effect (KIE) for Pd_6_ and Pd_3_Zn_3_/c by substituting H_2_ with D_2_ and using multiple approaches: zero-point vibrational energy (ZPVE) differences between the transition state and the first intermediate, Gibbs free energy barriers, and full vibrational partition functions via the Bigeleisen–Mayer formalism. The Gibbs- and Bigeleisen-based methods yielded comparable KIEs of 2.6–2.7 for Pd_6_ and 4.4 for Pd_3_Zn_3_, while ZPVE-based estimates were slightly lower (1.8 and 3.9 for Pd_6_ and Pd_3_Zn_3_, respectively). All methods yielded KIE values greater than 1, indicative of a normal isotope effect, where the reaction proceeds more slowly with D_2_ than H_2_.^76^ These values indicate a stronger isotopic sensitivity in the Zn-doped cluster and suggests that the H–H bond cleavage step plays a more significant role in the rate-determining transition state on Pd_3_Zn_3_ than on Pd_6_.

### CO_2_ Adsorption

CO_2_ adsorption was systematically investigated on bare Pd_6_, Pd_2_Zn_2_, and Pd_3_Zn_3_ clusters, for both intact (where the linear carbon dioxide binds with one of its oxygen atoms) and activated (where bent carbon dioxide binds through the carbon atom and one of its oxygen atoms) binding modes. The Pd_4_Zn_4_ cluster was not included in this CO_2_ adsorption study, as the adsorption trends could already be captured with the smaller Zn-doped systems. Additionally, computations on Pd_4_Zn_4_ were significantly more time-consuming due to its larger configurational space. For Pd_6_, CO_2_ adsorption was found to be significantly more favorable in the triplet state. Intact (physisorbed) CO_2_ adducts exhibit small interaction energies, of −21, −21 and −24 kJ/mol for Pd_6_, Pd_2_Zn_2_, and Pd_3_Zn_3_, respectively. On the other hand, adsorption energies for activated (chemisorbed) CO_2_ adducts span a wide range, from exothermic on Pd_6_ (−64 kJ/mol) to endothermic on Pd_2_Zn_2_ (+82 kJ/mol), and a slightly exothermic value of – 13 kJ/mol for Pd_3_Zn_3_, indicating a marked impact of Zn content on adsorption behavior. Earlier studies of CO_2_ physisorption reported similarly weak adsorption energies on both the pure Pd(111)^77^ surface (+9.6 kJ/mol, B3LYP/6–311++G­(2d))^74^ and on a Pd_4_ cluster (+22 kJ/mol, CCSD­(T)/def2-TZVPP),^54^ which are in line with the present values for intact CO_2_ adducts. To estimate the impact of the Basis Set Superposition Error (BSSE),^78,79^ we calculated the counterpoise correction for the CO_2_ binding modes of Pd_6_ and Pd_3_Zn_3_. BSSE destabilizes all adducts but by less than 3 kJ/mol, which is negligibly small for all four adducts considered.

Since the binding energy of the undissociated H_2_ (ranging between −50 and −76 kJ/mol) is about three times larger than that of CO_2_, the concentration of the hydrogen adducts is expected to be much larger than that of the CO_2_ adducts. Accordingly, it is reasonable to assume that in the CO_2_ hydrogenation reaction, the hydrogen adduct forms first, and that will react with CO_2_. This scenario is further supported by the typical use of hydrogen-rich feed gas to evaluate the catalytic activity of Pd/ZnO catalysts for CO_2_ hydrogenation to methanol.^11^ Based on these results, we conclude that for the Pd_*x*_Zn_*x*_ clusters, initiating CO_2_ hydrogenation through the activation of CO_2_ is highly unlikely; therefore, we will not pursue this option further In contrast, for pure Pd_6_ the quasi-barrierless activation (activation energy of 1 kJ/mol) of CO_2_ supports the feasibility of such a pathway. While Pd_6_ is primarily included as a reference system, we do examine its CO_2_-first scenario in the context of the intact H_2_ route (IH route).

### CO_2_ Hydrogenation

Based on the binding behavior of CO_2_ and H_2_, we propose two mechanistic routes for formate and carboxylate formation on the studied clusters, Pd_6_ and Pd_*x*_Zn_*x*_ (x = 2–4). The first route starts from a predissociated H_2_ adduct, followed by CO_2_ adsorption—referred to as the dissociated H_2_ route (HD route). The second involves coadsorption of CO_2_ on the preformed adduct with molecularly bound H_2_, referred to as the intact H_2_ route (IH route). The HD path assumes that, under reaction conditions, H_2_ activation precedes CO_2_ binding and that access to a dissociated H_2_ adduct is feasible. As discussed, activation of dihydrogen is feasible and thermodynamically favored in case of Pd_6_ and Pd_4_Zn_4_b, and also a reaction from Pd_3_Zn_3_/d and Pd_4_Zn_4_d cannot be ruled out, since the small barriers may allow population of this structure as well. The HD path from Pd_4_Zn_4_b leading to a formate adduct has a barrier exceeding 100 kJ/mol. We were able to locate a second HD path toward a formate adduct which exhibits a significantly lower barrier, however, the starting point of this is Pd_4_Zn_4_d, which is somewhat destabilized, but kinetically available dihydrogen adduct. This pathway is presented in Figure 4 together with the Pd_6_ and Pd_3_Zn_3_d HD paths, each leading to formate formation. The process begins with the formation of van der Waals (vdW) complexes between the dissociated H_2_ adducts and intact CO_2_ molecules. For all studied clusters, the adduct of the incoming CO_2_ with the H_2_ dissociated structure is stabilized, with an average energy drop of – 17 kJ/mol.

**Figure 4:**
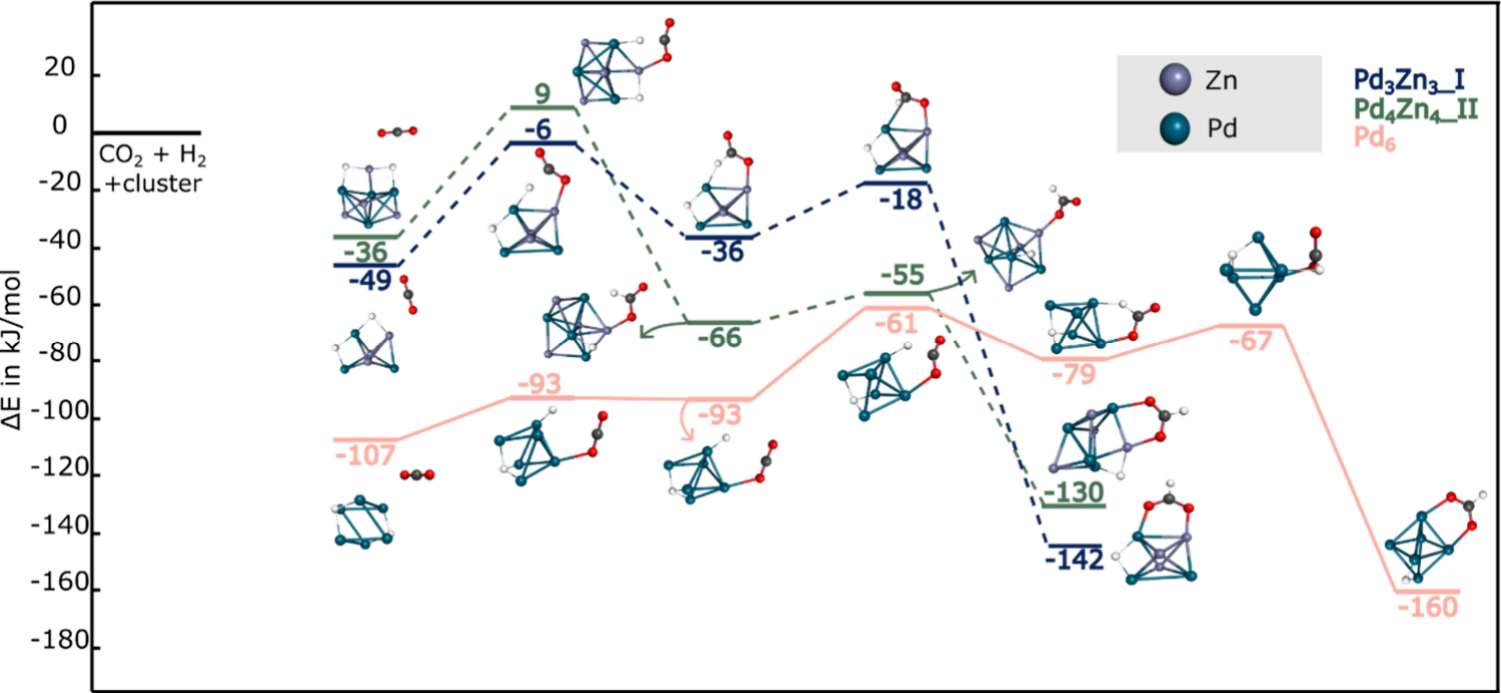
H_2_ dissociation route for the CO_2_ hydrogenation reaction on Pd_6_ and Pd_*x*_Zn_*x*_ (x = 3–4) toward formate. H, C, and O atoms are depicted as white, black, and red spheres, respectively.

For the Pd_6_ cluster, the vdW complex formed with CO_2_ requires 14 kJ/mol to activate a hydrogen atom by displacing it from its original μ_3_-coordination site. This is followed by a 32 kJ/mol barrier to activate the CO_2_ molecule and form a formate species bonded via its oxygen to a Pd atom. The HCOO group can then undergo a rotational rearrangement with a 12 kJ/mol barrier to reach the most stable formate configuration on the hydrogenated Pd_6_ cluster. The final product ist −160 kJ/mol below the gas-phase reactants, making it 67 kJ/mol more stable than the Pd_6_(2H) intermediate. Notably, the entire potential energy surface for formate formation on Pd_6_ remains on the triplet spin surface. Although the singlet–triplet energy gap is smaller for certain intermediates in this pathway (∼30 kJ/mol), compared to H_2_ dissociation, the overall reaction profile is consistently lower in energy on the triplet surface.

The hydrogenated forms of the bimetallic Pd_3_Zn_3_ and Pd_4_Zn_4_ clusters are less stable than the hydrogenated Pd_6_ adduct. In all PdZn clusters studied, CO_2_ prefers to bind through one of its oxygen atoms to a Zn site, with Zn–O distances of 2.80 and 2.92 Å for the Pd_3_Zn_3_(2H), and Pd_4_Zn_4_(2H) adducts shown in Figure 4, respectively. When the ZnH_2_ moiety is present (unlike the alternative isomers), the activation barriers for formate formation are consistently moderate: 62, 43, and 45 kJ/mol for Pd_2_Zn_2_, Pd_3_Zn_3_, and Pd_4_Zn_4_, respectively.

As mentioned above, the Zn atom in the ZnH_2_ moiety carries a substantial positive charge, which may contribute to the moderate activation energies observed for HCOO formation. However, the – 49 kJ/mol isomer of Pd_3_Zn_3_, which lacks this ZnH_2_ motif, also displays a comparably moderate barrier. In contrast, the hydrogenated Pd_4_Zn_4_ cluster exhibits a high barrier of 107 kJ/mol for formate formation. This highlights how structural differences, despite identical Pd:Zn stoichiometry, can result in distinct reactivity.

Like for Pd_6_, the intermediates for Pd_*x*_Zn_*x*_ (x = 2–4) can rearrange through low-energy steps to yield a thermodynamically stable formate product, where the HCOO group bridges a Pd and a Zn atom. The final relative energies for the formate adducts, calculated with respect to the bare cluster and gas-phase CO_2_ and H_2_, are – 177, −142, and – 130 kJ/mol for Pd_3_Zn_3__II, Pd_3_Zn_3__I, and Pd_4_Zn_4__II, respectively as partially shown in Figure 4. These values indicate that the formate-bound species are thermodynamically stable intermediates, capable of remaining adsorbed on the cluster surface and are available for subsequent H_2_ activation, possibly leading to methanol formation.

Based on Figure 4, we can conclude that, after overcoming the hydrogenation barrier, formate formation is feasible starting from Pd_6_, Pd_3_Zn_3_/d, and Pd_4_Zn_4_/d. Among these, the process is thermodynamically most favorable on the Pd_6_ cluster, with a final HCOO adsorption energy of −160 kJ/mol, compared to – 142 and – 130 kJ/mol for Pd_3_Zn_3_ and Pd_4_Zn_4_, respectively. It is worthy to note that we could locate further low barrier pathways to thermodynamically stable formate adducts from the thermodynamically most stable dissociated H_2_ adducts (from Pd_3_Zn_3__II in Figure S6, from Pd_2_Zn_2_ in Figure S7). This shows that once hydrogen dissociation can be catalyzed the formation of the formate is both kinetically and themodynamically feasible.

Notably, in the bimetallic clusters, the oxygen atoms that from the formate group consistently coordinate to both a Zn and a Pd atom—Zn being more electropositive than Pd and thus better at stabilizing negatively charged oxygen. Consistent with this, Natural Population Analysis (NPA) shows that the formate fragment carries a higher negative charge on the bimetallic clusters: – 0.77 au for Pd_4_Zn_4_ and Pd_2_Zn_2_, – 0.74 au for Pd_3_Zn_3_, compared to – 0.65 au on Pd_6_. This suggests that Zn coordination strengthens the electrostatic stabilization of the formate moiety; however, when considering both thermodynamics and kinetics, formate formation is more favored on the Pd_6_ cluster. Interestingly, Zn-bound formate species have also been reported as key intermediates in CO_2_ hydrogenation to methanol over Cu/ZnO catalysts,^80^ highlighting the broader relevance of Zn–O interactions in stabilizing formate across different catalytic systems.

While investigating the HD path, we find that the cluster size does not have a clear effect on the CO_2_ hydrogenation reaction. On Pd_2_Zn_2_ the pathway appears kinetically unfavorable, whereas Pd_3_Zn_3_ and Pd_4_Zn_4_ offer more alternative reaction pathways, owing to the larger number of possible isomers identified through ensemble analysis. Among these, some pathways are kinetically more favorable, while others lead to more stable (lower-energy) products.

Figure 5 presents the formate formation pathways via the IH route for the Pd_6_ and Pd_3_Zn_3_ clusters. These two systems were selected as representative cases; no further analysis of cluster size effects was pursued. While intact H_2_ binding appears to stabilize the CO_2_ adducts (−67 kJ/mol for Pd_6_ and −51 kJ/mol for Pd_3_Zn_3_), relative to the respective CO_2_ adducts (with adsorption energies of −64 kJ/mol for Pd_6_ and −24 kJ/mol for Pd_3_Zn_3_), a more relevant comparison, given our assumption that H_2_ adsorbs first, is to the H_2_ adducts. From this perspective, the CO_2_–H_2_ coadsorbed complex is strongly stabilized for Pd_6_ (−131 kJ/mol vs −69 kJ/mol, ΔE = −62 kJ/mol), but only slightly for Pd_3_Zn_3_ (−79 kJ/mol vs −76 kJ/mol, ΔE = −3 kJ/mol). This indicates that CO_2_ coadsorption is not significantly favored on Pd_3_Zn_3_. An alternative hydrogenated structure, Pd_3_Zn_3_H_2_/b (27 kJ/mol above the most stable H_2_ adduct), can bind CO_2_ with an interaction energy of approximately −26 kJ/mol. However, the resulting CO_2_–H_2_ adduct lies just 1 kJ/mol above the energy of the global H_2_ adduct, and therefore offers no substantial thermodynamic advantage. While this suggests that CO_2_ binding to higher-energy isomers is possible, the low population of Pd_3_Zn_3_H_2_/b and the high activation barrier observed for the formate formation pathway originating from this structure (Figure 5) limit its relevance under catalytic conditions.

**Figure 5:**
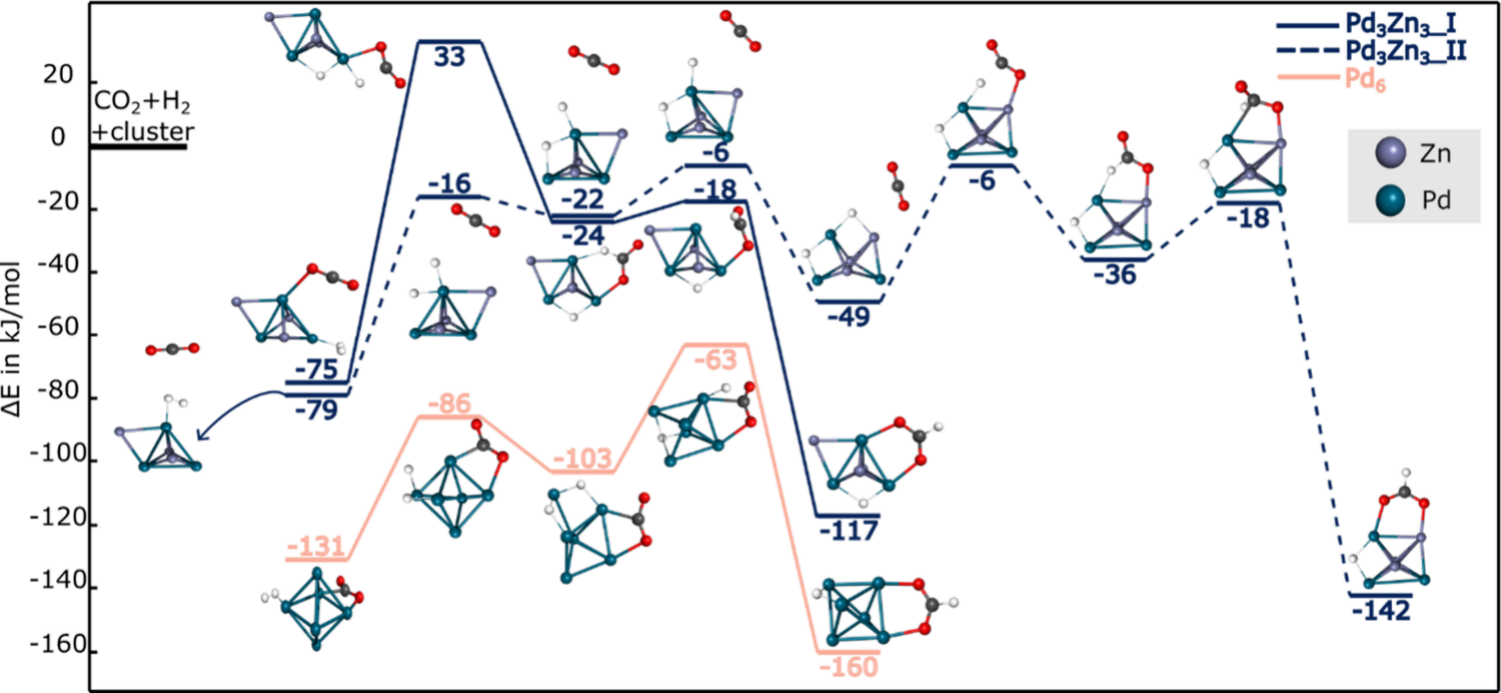
IH route for the CO_2_ hydrogenation reaction on Pd_6_ and Pd_3_Zn_3_ toward formate. Two paths are shown for Pd_3_Zn_3_ (with continuous and dashed lines). H, C, and O atoms are depicted as white, black, and red spheres, respectively. Relative energies are given in kJ/mol.

The calculated reaction pathways are summarized in Figure 5. The pathway on Pd_6_ is characterized by an initial barrier of +45 kJ/mol to dissociate H_2_ on the bent-CO_2_ adduct, followed by a + 40 kJ/mol barrier to transfer a bridged hydrogen to the carbon atom forming the formate intermediate. Also in the Pd_3_Zn_3__I pathway (Figure 5, blue dashed line) all barriers are submerged, with the highest barrier (+63 kJ/mol) corresponding to H_2_ splitting on the van der Waals complex of intact H_2_ and linear CO_2_ located at −79 kJ/mol. Basically, CO_2_ is mainly a spectator in this part of the process, in Figure 3 we have already presented this reaction (Pd_3_Zn_3__I), without CO_2_ the barrier was 72 kJ/mol. The rest of the reaction path is identical to the pathway shown in Figure 4, resulting in the formate adduct at −142 kJ/mol, adopting a bidentate geometry with Pd–O and Zn–O coordination. Unlike the pathways on Pd_6_ and Pd_3_Zn_3__II, the Pd_3_Zn_3__I pathway starts from an alternative Pd_3_Zn_3_ adduct (Figure 5, blue full line) initially involving a physisorbed CO_2_ with an intact H_2_, presenting a much higher initial barrier of +108 kJ/mol to dissociate H_2_ followed by only 6 kJ/mol needed for the formate rotation with a final product at −117 kJ/mol involving two Pd–O coordination.

In the final products, the formate unit carries NPA charges of −0.65 and −0.72 au for Pd_6_ (−160 kJ/mol) and Pd_3_Zn_3_ (−142 kJ/mol), respectively, suggesting a slightly enhanced charge stabilization in the bimetallic system.

To assess the thermodynamic relevance of entropy and temperature effects, we computed Gibbs free energy profiles (G, at 298.15 K and 1 atm) for the intact hydrogen (IH) formate pathway as depicted in Figure 5, for both the Pd_6_ and Pd_3_Zn_3__I clusters (the Gibbs free energy profile is shown in Figure S8). As expected, the translational entropy contribution to the Gibbs free energy is a significant destabilization factor in the adsorption reaction steps, and this effect is anticipated to increase further at higher temperatures. Thus, primarily due to entropy effects during the adsorption of the reactants, the Gibbs free energy profiles are elevated by up to ∼ 90 kJ/mol compared to the energy profile.

For Pd_6_, the thermal corrections do not significantly alter the progress of the reaction, as the height of the first barrier relative to the preceding reactant remains at 45 kJ/mol in both the electronic and Gibbs free energy profiles, and the pathway remains exergonic.

However, for Pd_3_Zn_3__I, we observe a more pronounced influence of thermal corrections on the progress of the reaction. While all transition structures are submerged in the energy profile, they are prominent on the Gibbs free energy profile. The energy of the highest-lying transition structure is −6 kJ/mol, while its Gibbs free energy is +79 kJ/mol. Additionally, the final formate product is destabilized from −142 kJ/mol (electronic) to −39 kJ/mol (Gibbs free energy).

When comparing the formate formation via the IH and HD routes, the rate-determining barriers for Pd_6_ are +32 and +45 kJ/mol, respectively, indicating that both routes are kinetically very similar and may be equally likely. For Pd_3_Zn_3_, the corresponding barriers are higher, at +63 and +72 kJ/mol, respectively. For Pd_3_Zn_3_, the intact CO_2_–H_2_ adduct is only slightly more stable than the preformed H_2_ adduct (−79 vs – 76 kJ/mol). This indicates that the preformed H_2_ adduct does not attract an incoming CO_2_ as was discussed above, therefore any subsequent reaction can only start after a rare event of direct collision. On the other hand, the destabilized Pd_3_Zn_3_d (+29 kJ/mol with respect to Pd_3_Zn_3_c), indicates a low population under reaction conditions, which makes the HD route unfavored.

Figure 6a presents the reaction energy profiles for CO_2_ hydrogenation to carboxylate (COOH) via the HD route on Pd_3_Zn_3_, Pd_4_Zn_4_, and Pd_6_ clusters. All pathways are thermodynamically favorable, with final carboxylate products ranging from – 93 to – 112 kJ/mol. Among the bimetallic clusters, Pd_3_Zn_3_ and Pd_4_Zn_4_ both yield COOH products at – 93 kJ/mol. The Pd_6_ cluster results in the most stabilized product (−112 kJ/mol). However, all pathways involve significant barriers: Pd_3_Zn_3_ and Pd_4_Zn_4_ each feature initial barriers of +171 and +178 kJ/mol, respectively, while Pd_6_, despite its deep starting point at – 141 kJ/mol, shows a + 164 kJ/mol activation energy. Although H_2_ dissociation on Pd_2_Zn_2_ is unlikely under typical conditions, we examined the hypothetical HD route to carboxylate. The reaction proceeds with a + 115 kJ/mol barrier, confirming its kinetic hindrance. These profiles highlight that although carboxylate formation is thermodynamically accessible on all clusters, the HD route is kinetically prohibited, given the substantial activation barriers observed for all clusters.

**Figure 6:**
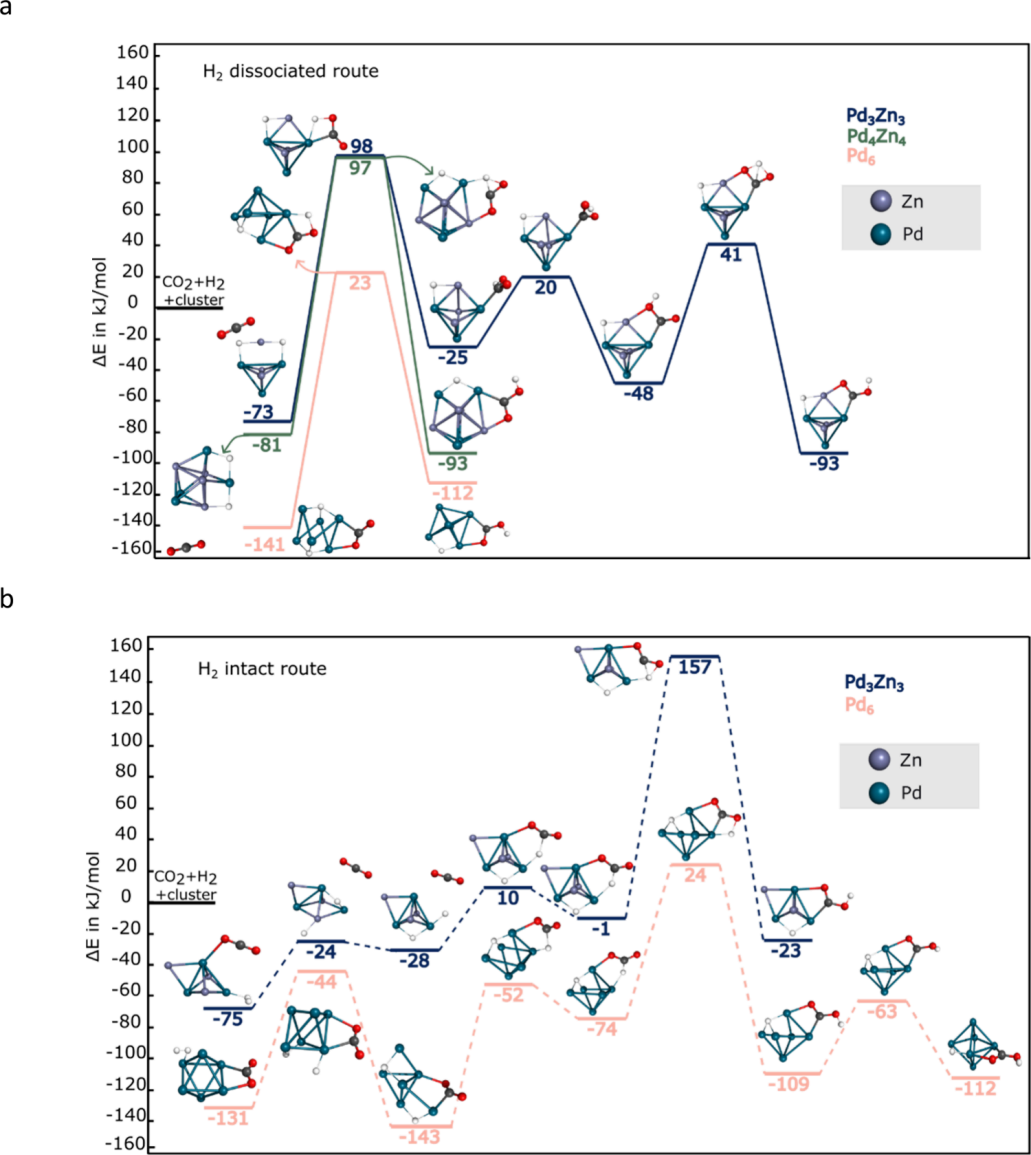
CO_2_ hydrogenation reaction paths on Pd_6_ and Pd_*x*_Zn_*x*_ (x = 3–4) toward carboxylate. a) H_2_ dissociated route. b) H_2_ intact route. H, C, and O atoms are depicted as white, black, and red spheres, respectively.

The bottom panel of Figure 6 illustrates the energy profiles for CO_2_ hydrogenation to carboxylate (COOH) via the H_2_ intact route on Pd_3_Zn_3_ and Pd_6_ clusters. For Pd_6_, the reaction begins at – 131 kJ/mol and proceeds through a complex sequence involving two key internal barriers. The first is a 87 kJ/mol increase to – 44 kJ/mol to dissociate H_2_. The second and highest barrier appears at +24 kJ/mol involving the hydrogen transfer from the carbon (formate intermediate) to oxygen (carboxylate intermediate), resulting in a barrier of 98 kJ/mol from the formate intermediate at −74 kJ/mol. Despite the reaction barriers, the final COOH product is significantly stabilized at – 112 kJ/mol (relative to the bare cluster, H_2_, and CO_2_) indicating a thermodynamically favorable overall process. However, when compared to the initial activated CO_2_ + H_2_ complex, the COOH adduct is less stable, indicating a thermodynamically uphill step relative to the activated CO_2_ + H_2_ complex at −131 kJ/mol.

In contrast, the Pd_3_Zn_3_ pathway begins at – 75 kJ/mol and climbs sharply to a transition state at +157 kJ/mol, yielding a final product at only – 23 kJ/mol. These results suggest that, while the intact H_2_ route to carboxylate on Pd_6_ is not barrierless, it is both kinetically and thermodynamically more favorable than on Pd_3_Zn_3_.

Comparing the reaction pathways shown in Figures 4, 5 and 6 clearly indicates that while HCOO formation is feasible, for both routes, on Pd_6_ and Pd_3_Zn_3_ clusters, COOH formation is highly unlikely, especially on Pd_3_Zn_3_. These findings align with previous results highlighting the importance of oxide surfaces in carboxylate formation on small Pd and PdZn metal clusters.^30^

Recent DFT studies on oxide-supported Pd-based systems, such as Pd_4_Zn_4_/TiO_2_,^30^ Pd_8_/TiO_2_,^30^ and Pd_5_Zn/ZrO_2_,^33^ have also explored the formate and carboxylate pathways for CO_2_ hydrogenation. In the Pd_5_Zn/ZrO_2_ system, the authors first model H_2_ dissociation on the cluster, and then introduce the resulting H atoms (H*) one at a time at each hydrogenation step, closely resembling our HD pathway. In contrast, the study on Pd_8_/TiO_2_ and Pd_4_Zn_4_/TiO_2_ assumes facile H_2_ activation by placing individual H atoms adsorbed on the cluster surface, treating hydrogen as predissociated.

In the Pd_4_Zn_4_/TiO_2_ study it was proposed that increasing Zn content weakens the CO_2_ adsorption and lowers the activation barriers for formate formation in Pd_8_/TiO_2_ and Pd_8‑n_Zn_n_/TiO_2_(n = 1–4) systems.^30^ While we have not quantified a direct correlation between the *d*-band center and CO_2_ adsorption in our gas-phase models, due to the lack of CO_2_ adsorption data for Pd_4_Zn_4_, we qualitatively observe that increasing Zn content brings noticeable shifts in the Pd *d*-band center, which may result in weaker CO_2_ binding. Despite the reduced CO_2_ adsorption strength (relative to Pd_6_), the formate pathway remains viable across compositions, owing to favorable transition state geometries. Moreover, the Pd_5_Zn/ZrO_2_ study highlights that Zn *d*-orbital contributions and cluster-support interface interactions reduce the barrier for carboxyl (COOH*) formation in comparison to the PdZn (101) surface.^33^

## Conclusions

We investigated how Pd–Zn alloying with a 1:1 ratio influences the structure and reactivity of small gas-phase Pd clusters as catalysts in CO_2_ hydrogenation. While Pd_6_ is a triplet, the studied Pd_*x*_Zn_*x*_ (x = 2–4) clusters have singlet ground state. As a first reaction step, H_2_ and CO_2_ adduct formation were both considered. For the intact reactants on the mixed Pd–Zn clusters the stability of the adducts is comparable to that on the Pd_6_ cluster. The H_2_ adducts are in all cases significantly more stable than the corresponding CO_2_ adducts, with about three times higher binding energies. The presence of Zn significantly reduces the stability of the activated adducts for both H_2_ (H_2_ dissociation) and CO_2_ (CO_2_ bending) reactants. While in the case of the pure Pd_6_ cluster both (H_2_ and CO_2_) activated adducts are more stable than the intact ones, all activated CO_2_ adducts of Pd_*x*_Zn_*x*_ clusters are less stable than the intact H_2_ adducts. Among the clusters studied, H_2_ dissociation is thermodynamically favored only for Pd_6_ and Pd_4_Zn_4_, and the associated activation barriers are low and moderate, respectively, indicating that these processes may occur under reaction conditions. In contrast, for Pd_2_Zn_2_ and Pd_3_Zn_3_, even the most stable dissociated hydrogen adducts are less stable than their intact H_2_ counterparts, and their formation is hindered by high kinetic barriers. In contrast to prior findings of facile H_2_ dissociation on Pd_8_/TiO_2_ ^30^ and Pd_5_Zn/ZrO_2_,^33^ our results show that H_2_ dissociation is only moderately favorable on most clusters and is associated with low barriers primarily in the Pd_6_ case. However, based on the computed tendencies with respect to the Pd_*x*_Zn_*x*_ cluster size, we expect that with further increase of the cluster size the hydrogen dissociation - which is a key step in the formate formation reaction will be possible.

For the further reaction steps, two mechanistic pathways were investigated: the intact H_2_ route, where CO_2_ reacts with molecularly adsorbed H_2_, and the hydrogen-dissociated route, where H_2_ dissociation precedes CO_2_ binding. In CO_2_ hydrogenation, formate formation dominates as the preferred pathway. Pd_6_ exhibits the lowest energy barriers and most favorable thermodynamics, showing that this cluster with triplet ground state can serve as an excellent catalyst, while Zn-containing clusters show more complex behavior. For Pd_2_Zn_2_ all reaction pathways are hindered by significant barriers, whether hydrogen splitting occurs in the presence or absence of CO_2_. The formation of the thermodynamically highly stable formate intermediates on the larger clusters is kinetically allowed on Pd_3_Zn_3_ and Pd_4_Zn_4_, although due to the relative instability of some intermediates to be attacked by the incoming CO_2_ a rather complex kinetics is expected for these pathways. Overall, while the reaction is essentially blocked on the smallest cluster, formate formation becomes possible on larger clusters. Carboxylate formation is also kinetically hindered across all clusters and is not expected to contribute significantly.

The reactivity is highly sensitive to cluster composition and geometry. Pd_6_ stands out as the most effective cluster for H_2_ activation and CO_2_ hydrogenation to formate, while Zn doping has an impact that varies depending on the mechanistic route and the specific cluster structure involved, but since only formate formation remains allowed, we can conclude that these Pd_*x*_Zn_*x*_ bimetallic catalysts pave the way for methanol formation.

## Supplementary Material



## Notes

Method benchmarking, low-energy isomers of the bare clusters and their H_2_/CO_2_ adducts, detailed electronic structure analysis, all located alternative reaction paths, HD reaction pathway for CO_2_ hydrogenation to formate and carboxilate on Pd_2_Zn_2_, Cartesian coordinates of the minima and transition structures (PDF)
