# Supplementary material for: CO2 Hydrogenation on Gas-Phase Palladium–Zinc Bimetallic Clusters
Source: ACS Omega. 2025 Sep 19;10(38):44234–47. doi: 10.1021/acsomega.5c05712 (PMC12489725; doi:10.1021/acsomega.5c05712)
Supplement: Supplementary file 1 [file ao5c05712_si_001.pdf]

CO_2 hydrogenation on gas-phase palladium-zinc bimetallic clusters

Bárbara Zamora Yusti,[a] Eszter Makkos,[a,b] Ewald Janssens,[c] László Nyulászi,[a,b] Tibor Höltzl[a,b,d]*

[a], Budapest University of Technology and Economics, Department of inorganic and Analytical Chemistry, Műegyetem rkp. 3. H1111, Budapest, Hungary

[b], HUN-REN-BME Computation Driven Chemistry Research Group, Budapest University of Technology and Economics, Műegyetem rkp. 3. H1111, Budapest, Hungary

[c], KU Leuven, Quantum Solid-State Physics, Department of Physics and Astronomy, Celestijnenlaan 200D, B3001 Leuven, Belgium

[d] Nanomaterials Science Group, Furukawa Electric Institute of Technology, Késmárk utca 28/A, H1158, Budapest, Hungary

Contents

List of tables S3

List of figures S4

Method benchmarks S5

Bond lengths and electronic structure-based descriptors S12

Ensemble of structures in the CO_2 hydrogenation on palladium-zinc clusters S18

HCOO and COOH formation on the studied clusters S19

Projected density of states (PDOS) S21

Cartesian coordinates (in Å) S22

List of tables

Table S1. *Method benchmarks for PdH and ZnH.* 5
Table S2. *Method benchmarks for Pd2.* 6
Table S3. *Method benchmarks for* PdH_2. 7
Table S4. *Method benchmarks for* $PdCO_2$. 8
Table S5. *The Root Mean Square Deviation (RMSD) and Maximum Absolute Error (MAE) values derived for all small systems (Pd + H → PdH, Zn+H → ZnH, Pd + Pd →* Pd_2, *Pd +* H_2 *→* PdH_2, *PdH + H →* PdH_2,*Pd +* CO_2 *→* $PdCO_2$*) presented in Tables 1-4 using a series of DFT functionals, each applied with the def2-TZVP basis set.* 9
Table S6. *Reaction energies (*E_{reac}*) of different adduct formations on the studied palladium-zinc clusters calculated with a series of DFT functionals, each applied with the def2-TZVP basis set. Frozen core approximated CCSD(T)/def2-TZVPPD single point energies were obtained with the PBE0-D3/def2-TZVP geometries and used as a reference to calculate the Signed Error.* 10
Table S7. *Statistical analysis of the benchmark results presented in Table S 6 for small palladium-zinc clusters. (RMSD: Root Mean Square Deviation of the reaction energies calculated for each DFT functionals from the reference value of CCSD(T)/def2-TZVPPD; MAE: Maximum Absolute Error, largest error calculated with each functional respect to the reference value.)* 11
Table S8. *Gas-phase bare clusters, relative energies and spin multiplicities.* 12
Table S9. *Natural charges for each atom in the* H_2 *adducts of* Pd_2Zn_2, Pd_3Zn_3, Pd_4Zn_4 *and* Pd_6. 13
Table S10. *Collected bond lengths (Å) electron densities at the bond critical points (BCP) and Laplacians (a.u.) for the* Pd_6, Pd_2Zn_2, Pd_3Zn_3, Pd_4Zn_4 *clusters.* 15
Table S11. *Collected bond lengths (Å) electron densities at the bond critical points (BCP) and Laplacians (a.u.) for the intact* H_2 *adducts of the* Pd_6, Pd_2Zn_2, Pd_3Zn_3, Pd_4Zn_4 *clusters.* 16
Table S12. *Collected bond lengths (Å) electron densities at the bond critical points (BCP) and Laplacians (a.u.) for the dissociated* H_2 *adducts of the* Pd_6,Pd_2Zn_2, Pd_3Zn_3, Pd_4Zn_4 *clusters.* 17
Table S13. *Basis-set superposition error (BSSE) of the* CO_2 *adducts of* Pd_6 *and* Pd_3Zn_3 *(from Figure S5). Basis-set superposition error (BSSE) is the difference between the uncorrected and the counterpoise corrected binding energies.* 19
Table S14. *Total natural atomic charges of the COOH and HCOO groups (from Figure 4) adsorbed on gas-phase* Pd_6, Pd_2Zn_2, Pd_3Zn_3 *and* Pd_4Zn_4 *clusters.* 19
Table S15. *Zn and Pd d-band centres for the bare clusters.* 21
Table S16. *Cartesian coordinates and SCF energies of neutral bare clusters from Table S8.* 22
Table S17. *Cartesian coordinates and SCF energies of adducts from Figure 2.* 22
Table S18. *Cartesian coordinates and SCF energies of adducts from Figure 3, reaction paths for* H_2 *dissociation.* 23
Table S19. *Cartesian coordinates and SCF energies of adducts from Figure S3.* 24
Table S20. *Cartesian coordinates and SCF energies of adducts from Figure S4.* 24
Table S21. *Cartesian coordinates and SCF energies of* CO_2 *adducts from Figure S5.* 24
Table S22. *Cartesian coordinates and SCF energies of structures in formate reaction paths (Figure 4).* .. 25
Table S23. *Cartesian coordinates and SCF energies of structures in Figure S6.* 26
Table S24. *Cartesian coordinates and SCF energies of structures in Figure 5.* 27
Table S25. *Cartesian coordinates and SCF energies of structures in COOH group reaction paths (Figure 6 Panel A).* 28
Table S26. *Cartesian coordinates and SCF energies of structures in COOH group reaction paths (Figure 6 Panel B).* 29
Table S27. *Cartesian coordinates and SCF energies of structures in* Pd_2Zn_2 *reaction paths (Figure S7 upper panel).* 30
Table S28. *Cartesian coordinates and SCF energies of structures in* Pd_2Zn_2 *reaction paths (Figure S7 lower panel).* 30

List of figures

Figure S1 *.Natural charges for each atom in the gas-phase Pd_2Zn_2, Pd_3Zn_3, Pd_4Zn_4 and Pd_6 clusters. 12*
Figure S2*. Localized molecular orbitals (LMOs) involving hydrogen for the intact (left panel) and dissociated (right panel) hydrogen adducts of Pd_6, Pd_2Zn_2, Pd_3Zn_3, and Pd_4Zn_4 clusters. 14*
Figure S3. *Geometries of the most stable CO_2 adducts of Pd_6H_2. Relative energies compared to the lowest energy isomers are indicated in kJ/mol. 18*
Figure S4. *Geometries of the most stable CO_2 adducts of $Pd_3Zn_3H_2$. Relative energies compared to the lowest energy isomer are indicated in kJ/mol. 18*
Figure S5. *CO_2 adducts for Pd_6, Pd_2Zn_2 and Pd_3Zn_3. Relative energies compared to the lowest energy isomer are free CO_2 indicated in kJ/mol. 18*
Figure S6*. Alternative H_2 dissociation route for the CO_2 hydrogenation reaction on Pd_4Zn_4_I and Pd_3Zn_3_II towards formate. H, C, and O atoms are depicted as white, black, and red spheres, respectively. Energies are included in kJ/mol. 19*
Figure S7*. Dissociated H_2 (HD) route for the CO_2 hydrogenation to formate (upper panel) and carboxylate (lower panel) on Pd_2Zn_2. H, C, and O atoms are depicted as white, black, and red spheres, respectively. Energies are included in kJ/mol. 20*
Figure S8*. Gibbs free energy profiles at 298K for the intact H_2 (IH) route for the CO_2 hydrogenation to formate on Pd_6 and Pd_3Zn_3_II (from Figure 5). H, C, and O atoms are depicted as white, black, and red spheres, respectively. 20*
Figure S9*. Projected density of states (PDOS) (panels arranged left-to-right, top-to-bottom) for: Pd_6, Pd_2Zn_2, Pd_3Zn_3, and Pd_4Zn_4. For the PdZn clusters, fragment 1 is composed of Zn atoms and fragment 2 is composed of Pd atoms. 21*
Figure S10*. Correlation between the adsorption energy of PdZn(2H) adducts and the Pd d-band center. 21*

Method benchmarks

Table S1. Method benchmarks for PdH and ZnH.

	Pd + H → PdH			Zn+H → ZnH		
**Method**	**E_{reac} (kJ/mol)**	**Unsigned Error[c] (kJ/mol)**	**d (Å)**	**E_{reac} (kJ/mol)**	**Unsigned Error [c] (kJ/mol)**	**d (Å)**
**Best estimate[a]**	-239.8	0.0	-	-91.5	0.0	-
**CCSD(T, Full)/aug-cc-pwCVTZ-PP, MRCC[b]**	-237.8	-2.0	-	-88.9	2.6	-
**CCSD(T, Full)/aug-cc-pwCVQZ-PP, MRCC[b]**	-236.7	-3.1	-	-90.4	1.1	-
**CCSD(T, Full)$_{Λ}$/aug-cc-pwCVTZ-PP, MRCC[b]**	-236.5	-3.3	-	-89.5	2.0	-
**CCSD(T, FC)/aug-cc-pVTZ-PP, MRCC[b]**	-226.2	-13.5	-	-91.2	0.4	-
**CCSDT(Q,FC)/aug-cc-pVTZ-PP, MRCC[b]**	-229.3	-10.5	-	-92.2	-0.7	-
**CCSD(T, Full)/aug-cc-pwCVQZ-PP**	-235.8	-4.0	1.522	-90.4	1.1	1.588
**CCSD(T, FC)/def2-TZVPPD[b**	-231.2	-8.5	-	-99.6	-8.1	-
**B3LYP/aug-cc-pwCVQZ-PP**	-249.9	10.2	1.538	-91.5	0.1	1.611
**B3LYP-D3(OP)/aug-cc-pwCVQZ-PP**	-250.1	10.4	1.538	-91.7	-0.1	1.612
**B3LYP-D4/aug-cc-pwCVQZ-PP**	-252.5	12.7	1.539	-94.0	-2.5	1.614
**TPSSh/aug-cc-pwCVQZ-PP**	-266.3	26.5	1.543	-117.8	-26.3	1.601
**B3LYP/def2-TZVP**	-249.5	9.8	1.537	-94.5	-3.0	1.627
**TPSSh/def2-TZVP**	-266.5	26.8	1.541	-120.4	-28.9	1.614
**ωB97M-V/def2-TZVP**	-227.7	-12.1	1.542	-99.8	-8.3	1.605
**CAM-B3LYP/def2-TZVP**	-243.5	3.8	1.536	-102.2	-10.6	1.610
**PBE-D3(OP)/def2-TZVP**	-263.2	23.5	1.529	-93.4	-1.9	1.624
**PBE0-D3(OP)/def2-TZVP**	-240.2	0.4	1.529	-100.2	-8.7	1.615
**PBE0-D3(OP)/aug-cc-pwCVQZ-PP**	-241.5	1.8	1.530	-97.3	-5.7	-1.600

(a) E(CCSD(T, Full)/aug-cc-pwCVQZ-PP) + (E(CCSDT(Q,FC)/aug-cc-pVTZ-PP)-E(CCSD(T, FC)/aug-cc-pVTZ-PP))

(b) Single point energies computed on CCSD(T, FC)/aug-cc-pwCVQZ-PP geometries

(c) Unsigned error compared to best estimate

Table S2. Method benchmarks for Pd2.

	Pd + Pd → Pd_2		
	**E_{reac} (kJ/mol)**	**Unsigned Error (c) (kJ/mol)**	**d (Å)**
**Best estimate**[(a)]	-71.4	0.0	2.437
**CCSD(T, Full)/aug-cc-pwCVTZ-PP, MRCC**[(b)]	-	-	-
**CCSD(T, Full)/aug-cc-pwCVQZ-PP, MRCC**[(b)]	-	-	-
**CCSD(T, Full)_lambda/aug-cc-pwCVTZ-PP, MRCC**[(b)]	-	-	-
**CCSD(T, FC)/aug-cc-pVTZ-PP, MRCC**[(b)]	-	-	-
**CCSDT(Q,FC)/aug-cc-pVTZ-PP, MRCC**	-	-	-
**CCSD(T, Full)/aug-cc-pwCVQZ-PP**	-	-	-
**CCSD(T, FC)/def2-TZVPPD**	-53.8	17.6	-
**B3LYP/aug-cc-pwCVQZ-PP**	-98.8	-27.4	2.490
**B3LYP-D3(OP)/aug-cc-pwCVQZ-PP**	-103.0	-31.6	2.507
**B3LYP-D4/aug-cc-pwCVQZ-PP**	-110.4	-39.0	2.533
**TPSSh/aug-cc-pwCVQZ-PP**	-	-	-
**B3LYP/def2-TZVP**	-101.0	-29.6	2.505
**TPSSh/def2-TZVP**	-132.3	-60.9	2.484
**ωB97M-V/def2-TZVP**	-45.9	25.5	2.494
**CAM-B3LYP/def2-TZVP**	-79.5	-8.1	2.485
**PBE-D3(OP)/def2-TZVP**	-151.7	-80.3	2.500
**PBE0-D3(OP)/def2-TZVP**	-101.4	-30.0	2.487
**PBE0-D3(OP)/aug-cc-pwCVQZ-PP**	-101.7	-30.3	2.470

(a) CCSD(T, Full)/CBS-aug-cc-pwCV(Q,5)Z, geometry: CCSD(T, FC)/def2-QZVPPD

(b) Single point energies computed on CCSD(T, FC)/def2-QZVPPD geometries

(c) Unsigned error compared to best estimate

Table S3. Method benchmarks for PdH_2.

	$Pd + H_2 \rightarrow PdH_2$		$PdH + H \rightarrow PdH_2$	
	E_{reac} (kJ/mol)	Unsigned Error(c) (kJ/mol)	E_{reac} (kJ/mol)	Unsigned Error(c) (kJ/mol)
**Best estimate[(a)]**	-87.6	0.0	-304.6	0.0
**CCSD(T, Full)/aug-cc-pwCVTZ-PP, MRCC[(b)]**	-87.4	0.2	-303.8	0.8
**CCSD(T, Full)/aug-cc-pwCVQZ-PP, MRCC[(b)]**	-87.9	-0.3	-308.0	-3.4
**CCSD(T, Full)_lambda/aug-cc-pwCVTZ-PP, MRCC[(b)]**	-	-	-	-
**CCSD(T, FC)/aug-cc-pVTZ-PP, MRCC[(b)]**	-79.6	8.0	-307.6	-3.0
**CCSDT(Q,FC)/aug-cc-pVTZ-PP, MRCC[(b)]**	-79.3	-	-304.2	0.4
**CCSD(T, Full)/aug-cc-pwCVQZ-PP[(b)]**	-	-	-	-
**CCSD(T, FC)/def2-TZVPPD[(b)]**	-83.6	4.0	-306.1	-1.5
**B3LYP/aug-cc-pwCVQZ-PP**	-79.7	7.8	-291.2	13.3
**B3LYP-D3(OP)/aug-cc-pwCVQZ-PP**	-80.2	7.4	-291.5	13.1
**B3LYP-D4/aug-cc-pwCVQZ-PP**	-83.8	3.7	-293.1	11.5
**TPSSh/aug-cc-pwCVQZ-PP**	-	-	-	-
**B3LYP/def2-TZVP**	-78.0	9.5	-288.9	15.7
**TPSSh/def2-TZVP**	-89.8	-2.2	-294.1	10.5
**ωB97M-V/def2-TZVP**	-74.7	12.9	-299.7	4.9
**CAM-B3LYP/def2-TZVP**	-81.6	6.0	-293.8	10.7
**PBE-D3(OP)/def2-TZVP**	-115.5	-28.0	-290.1	14.5
**PBE0-D3(OP)/def2-TZVP**	-90.2	-2.7	-286.5	18.1
**PBE0-D3(OP)/aug-cc-pwCVQZ-PP**	-93.0	-5.5	-288.5	16.1

(a) CCSD(T,Full)/aug-cc-pVQZ-PP + CCSDT(Q, FC)/aug-cc-pwCVTZ-PP – CCSD(T, FC)/aug-cc-pwCVTZ-PP, geometry: B3LYP/aug-cc-pwCVQZ-PP

(b) Single point energies computed on B3LYP/aug-cc-pwCVQZ-PP geometries

(c) Unsigned error compared to best estimate

Table S4. Method benchmarks for $PdCO_2$.

	$Pd + CO_2 \rightarrow PdCO_2$	
	E_{reac} (kJ/mol)	Unsigned Error (kJ/mol)
**Best estimate**	-26.8	0.0
**CCSD(T, Full)/aug-cc-pwCVTZ-PP, MRCC**	-25.2	1.6
**CCSD(T, Full)/aug-cc-pwCVQZ-PP, MRCC**	-25.3	1.5
**CCSD(T, Full)_lambda/aug-cc-pwCVTZ-PP, MRCC**	-	-
**CCSD(T, FC)/aug-cc-pVTZ-PP, MRCC**	-22.0	4.8
**CCSDT(Q,FC)/aug-cc-pVTZ-PP, MRCC**	-23.5	-
**CCSD(T, Full)/aug-cc-pwCVQZ-PP**	-	-
**CCSD(T, FC)/def2-TZVPPD**	-27.8	-1.0
**B3LYP/aug-cc-pwCVQZ-PP**	-20.0	6.8
**B3LYP-D3(OP)/aug-cc-pwCVQZ-PP**	-24.3	2.5
**B3LYP-D4/aug-cc-pwCVQZ-PP**	-29.9	-3.1
**TPSSh/aug-cc-pwCVQZ-PP**	-	-
**B3LYP/def2-TZVP**	-22.5	4.3
**TPSSh/def2-TZVP**	-53.4	-26.6
**ωB97M-V/def2-TZVP**	-20.3	6.4
**CAM-B3LYP/def2-TZVP**	-20.8	5.9
**PBE-D3(OP)/def2-TZVP**	-72.3	-45.5
**PBE0-D3(OP)/def2-TZVP**	-36.8	-10.0
**PBE0-D3(OP)/aug-cc-pwCVQZ-PP**	-35.4	-8.6

(a) CCSD(T,Full)/aug-cc-pVQZ-PP + CCSDT(Q, FC)/aug-cc-pwCVTZ-PP – CCSD(T, FC)/aug-cc-pwCVTZ-PP, geometry: B3LYP/aug-cc-pwCVQZ-PP

(b) Single point energies computed on B3LYP/aug-cc-pwCVQZ-PP geometries

(c) Unsigned error compared to best estimate

Table S5. The Root Mean Square Deviation (RMSD) and Maximum Absolute Error (MAE) values derived for all small systems (Pd + H → PdH, Zn+H → ZnH, Pd + Pd → Pd_2, Pd + H_2 → PdH_2, PdH + H → PdH_2, Pd + CO_2 → $PdCO_2$) presented in Tables 1-4 using a series of DFT functionals, each applied with the def2-TZVP basis set.

	RMSD (kJ/mol)	MAE (kJ/mol)
B3LYP	6.1	29.6
ωB97M-V	7.2	26.6
CAM-B3LYP	2.9	10.6
TPSSh	12.3	60.9
PBE-D3(OP)	16.7	80.3
PBE0-D3(OP)	6.3	30

Benchmark computations were carried out for simple test systems of Pd_2, PdH, ZnH, PdH_2 and PCO_2, including correlation level up to CCSDT(Q) and semicore-valence correlation using CCSD(T) (Table S1- 5). Please note that the geometries and spin states were selected to match those found in larger clusters, thus may not be the lowest energy ones. There are small differences between the results obtained using CCSDT(Q) and CCSD(T). Semicore-valence correlation is known to be important in Pd_2, thus it is expected to play a significant role in computing accurate atomization energies of Pd clusters. However, according to our benchmark computations, semicore-valence correlation plays only a minor role in the reactions of Pd with H, H_2 or CO_2.

The benchmark results show that among the tested DFT functionals, PBE0-D3 predicted the PdH and Pd-H_2 formation to be closer to the best estimate, while B3LYP performed better for the reaction energies of ZnH and Pd-CO_2. Two long-range hybrid functionals were also tested: ωB97M-V performed similarly to B3LYP and PBE0-D3 but severely underestimated the formation energy of a Pd_2 dimer. The reaction energies calculated with the commonly applied PBE-D3 dispersion-corrected GGA functional deviate to a largest extent from the best available estimate. Among the frequently employed hybrid functionals, TPSSh yielded the largest errors, with a maximum absolute error of 60.9 kJ/mol, however, it worked well for the H_2 or CO_2 adsorption on Pd. Overall, among all tested DFT functionals B3LYP, PBE0-D3, and ωB97M-V exhibited similarly good performances, while CAM-B3LYP achieved the best agreement with the benchmark reaction, providing a remarkably close reaction energy for the Pd_2 dimer formation to the best estimate (-8.1 kJ/mol signed error) and generally performing well in all other test cases.

Table S6. Reaction energies (E_{reac}) of different adduct formations on the studied palladium-zinc clusters calculated with a series of DFT functionals, each applied with the def2-TZVP basis set. Frozen core approximated CCSD(T)/def2-TZVPPD single point energies were obtained with the PBE0-D3/def2-TZVP geometries and used as a reference to calculate the Signed Error.

	**Intact H_2 adduct**		**Dissociated H_2 adduct**		**CO_2 adduct, HCOO/COOH formation**	
	**$Pd_2Zn_2+H_2 \rightarrow Pd_2Zn_2(H_2)$**		**$Pd_2Zn_2+H_2 \rightarrow Pd_2Zn_2(HH)$**		**$Pd_2Zn_2+CO_2 \rightarrow Pd_2Zn_2(CO_2)$**	
	Ereac (kJ/mol)	Signed Error (kJ/mol)	Ereac (kJ/mol)	Signed Error (kJ/mol)	Ereac (kJ/mol)	Signed Error (kJ/mol)
**CCSD(T)/def2-TZVPPD***	**-68.2**	**0**	**-52.5**	**0**	**-28.2**	**0**
B3LYP	-53.1	15.1	-16.1	36.4	-11.5	16.7
ωB97M-V	-64.0	4.1	-44.5	8.0	-21.3	6.9
ωB97X-D	-63.2	5.0	-36.7	15.8	-18.3	9.9
CAM-B3LYP	-64.2	3.9	-38.7	13.8	-18.3	9.8
B3LYP-D3(OP)	-56.1	12.0	-21.7	30.8	-20.4	7.8
TPSSh-D3(OP)	-62.6	5.6	-43.3	9.1	-19.0	9.2
PBE-D3(OP)	-76.4	-8.2	-49.1	3.4	-24.3	3.9
PBE0-D3(OP)	-67.7	0.5	-49.5	2.9	-20.7	7.5
	**$Pd_3Zn_3+H_2 \rightarrow Pd_3Zn_3(H_2)$**		**$Pd_3Zn_3+H_2 \rightarrow Pd_3Zn_3(HH)$**		**$Pd_2Zn_2(HH)+CO_2 \rightarrow Pd_2Zn_2H(HCOO)$**	
	Ereac (kJ/mol)	Signed Error (kJ/mol)	Ereac (kJ/mol)	Signed Error (kJ/mol)	Ereac (kJ/mol)	Signed Error (kJ/mol)
**CCSD(T)/def2-TZVPPD***	**-75.5**	**0**	**-64.8**	**0.0**	**-150.6**	**0**
B3LYP	-57.2	18.4	-17.9	46.9	-86.2	64.4
ωB97M-V	-69.5	6.0	-55.6	9.2	-145.6	5.1
ωB97X-D	-68.2	7.3	-44.3	20.5	-125.6	25.1
CAM-B3LYP	-68.9	6.6	-43.8	21.1	-134.8	15.8
B3LYP-D3(OP)	-62.9	12.6	-28.3	36.6	-104.9	45.8
TPSSh-D3(OP)	-69.2	6.3	-58.3	6.5	-130.0	20.6
PBE-D3(OP)	-81.1	-5.6	-58.9	5.9	-126.3	24.4
PBE0-D3(OP)	-75.9	-0.4	-59.9	4.9	-137.3	13.3
	**$Pd_4Zn_4+H_2 \rightarrow Pd_4Zn_4(H_2)$**		**$Pd_4Zn_4+H_2 \rightarrow Pd_4Zn_4(HH)$**		**$Pd_2Zn_2(HH)+CO_2 \rightarrow Pd_2Zn_2H(COOH)$**	
	Ereac (kJ/mol)	Signed Error (kJ/mol)	Ereac (kJ/mol)	Signed Error (kJ/mol)	Ereac (kJ/mol)	Signed Error (kJ/mol)
**CCSD(T)/def2-TZVPPD***	**-48.2**	**0**	**-59.2**	**0**	**-59.6**	**0**
B3LYP	-32.3	16.0	-5.3	53.9	9.1	68.7
ωB97M-V	-48.8	-0.6	-20.2	39.1	-56.2	3.4
ωB97X-D	-46.3	2.0	-19.6	39.6	-30.2	29.4
CAM-B3LYP	-44.8	3.5	-15.5	43.8	-36.5	23.1
B3LYP-D3(OP)	-38.6	9.6	-23.3	35.9	-8.5	51.1
TPSSh-D3(OP)	-42.9	5.3	-64.7	-5.4	-32.7	26.9
PBE-D3(OP)	-52.6	-4.3	-66.4	-7.2	-44.8	14.8
PBE0-D3(OP)	-48.9	-0.6	-62.9	-3.6	-49.4	10.2

The benchmark calculations on adduct formation of the investigated Pd_xZn_x clusters are summarised in Table S6. Assuming that the use of dispersion corrections or long-range functionals would be beneficial for accurately describing the formation of intact H_2 and CO_2 adducts, we applied the D3(OP) correction to each functional tested that does not include a built-in dispersion term. (B3LYP was employed both with and without D3).

Statistical analysis based on the reaction energies of Table S6 is summarised in Table S7. Independent of the applied functional, results for intact H_2 adduct formation ($Pd_xZn_x(H_2)$) showed significantly smaller deviations from the reference values (RMSD between 0.3 and 9.5 kJ/mol) than the dissociated H_2 structures (Pd_xZn_x(HH) RMSD: 2.3-26.7 kJ/mol). Interestingly, while B3LYP generally performed well for the small test systems in Table S1Table S4, it consistently yielded the largest errors for the Pd_xZn_x results in Table S6 , independently of the application of D3 correction. CAM-B3LYP, which was the best-performing functional for the small systems, yielded similar values to the other long-range DFT functionals: ωB97M-V and ωB97XD. TPPSh-D3 and PBE-D3, which appeared to be the worst-performing functionals for our purposes in Table S1, were ranked among the top three functionals in terms of accuracy for the considered systems in Table S6. The PBE0-D3 functional consistently demonstrated good performance among all the studied systems, often yielding the most accurate results, with a few exceptions, such as the Pd_2Zn_2-HCOO and -COOH formation cases, in which it was the second-best functional behind ωB97M-V.

Based on the results presented in Table S1 Table S7, we have selected the PBE0-D3/def2-TZVP method, which has shown consistently good performance in the benchmark calculations, with no instances of low accuracy.

Table S7. Statistical analysis of the benchmark results presented in Table S 6 for small palladium-zinc clusters. (RMSD: Root Mean Square Deviation of the reaction energies calculated for each DFT functionals from the reference value of CCSD(T)/def2-TZVPPD; MAE: Maximum Absolute Error, largest error calculated with each functional respect to the reference value.)

	**$Pd_xZn_x(H_2)$**		**Pd_xZn_x(HH)**		**$Pd_2Zn_2(CO_2)$/ (H_HCOO)/(H_COOH)**		**Pd_xZn_x_All**	
	RMSD (kJ/mol)	MAE (kJ/mol)	RMSD (kJ/mol)	MAE (kJ/mol)	RMSD (kJ/mol)	MAE (kJ/mol)	**RMSD (kJ/mol)**	**MAE (kJ/mol)**
B3LYP	9.5	18.4	26.7	53.9	31.9	68.7	14.2	68.7
omegaB97M-V	2.4	6.0	13.6	39.1	3.1	6.9	4.7	39.1
omegaB97XD	3.0	7.3	15.8	39.6	13.3	29.4	6.9	39.6
CAM-B3LYP	2.8	6.6	16.8	43.8	9.9	23.1	6.6	43.8
B3LYP-D3(OP)	6.6	12.6	19.9	36.6	23.0	51.1	10.4	51.1
TPSSh-D3(OP)	3.3	6.3	4.2	9.1	11.7	26.9	4.3	26.9
PBE-D3(OP)	3.6	8.2	3.3	7.2	9.6	24.4	3.6	24.4
PBE0-D3(OP)	0.3	0.6	2.3	4.9	6.1	13.3	2.2	13.3

Bond lengths and electronic structure-based descriptors

Table S8. Gas-phase bare clusters, relative energies and spin multiplicities.

Cluster	Relative energies (kJ/mol) and spin multiplicities (M)			
Pd_6	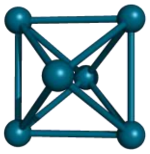 0 (M=3)<br>45 (M=1)<br>66 (M=5)		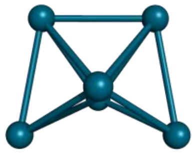 88 (M=1)	
Pd_2Zn_2 (M=1)	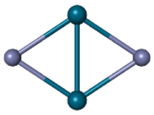 0			
Pd_3Zn_3 (M=1)	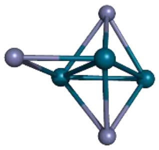 0		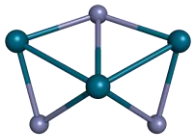 47	
Pd_4Zn_4 (M=1)	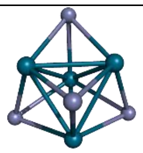 0	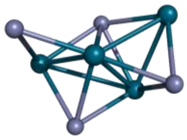 15	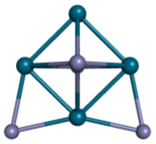 22	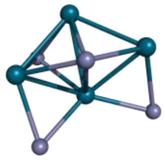 39

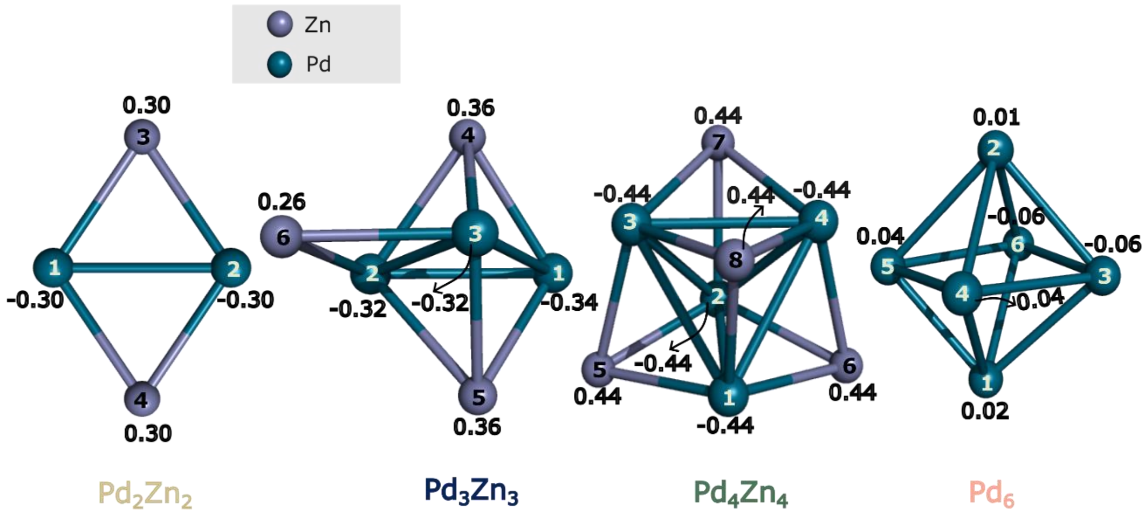

Figure S1 .Natural charges for each atom in the gas-phase Pd_2Zn_2, Pd_3Zn_3, Pd_4Zn_4 and Pd_6 clusters.

Table S9. Natural charges for each atom in the H_2 adducts of Pd_2Zn_2, Pd_3Zn_3, Pd_4Zn_4 and Pd_6.

Intact H_2 adducts											
Pd_6			Pd_2Zn_2			Pd_3Zn_3			Pd_4Zn_4		
**Atom**	**No**	**Charge**	**Atom**	**No**	**Charge**	**Atom**	**No**	**Charge**	**Atom**	**No**	**Charge**
Pd	1	-0.20	Pd	1	-0.35	Pd	1	-0.33	Pd	1	-0.44
Pd	2	-0.01	Pd	2	-0.35	Pd	2	-0.42	Pd	2	-0.43
Pd	3	0.04	Zn	3	0.35	Pd	3	-0.37	Pd	3	-0.43
Pd	4	0.08	Zn	4	0.34	Zn	4	0.40	Pd	4	-0.43
Pd	5	0.05	H	5	0.00	Zn	5	0.31	Zn	5	0.45
Pd	6	0.04	H	6	0.00	Zn	6	0.40	Zn	6	0.45
H	7	0.00				H	7	0.00	Zn	7	0.45
H	8	-0.00				H	8	0.01	Zn	8	0.37
									H	9	-0.00
									H	10	-0.00
Dissociated H_2 adducts											
Pd_6			Pd_2Zn_2			Pd_3Zn_3			Pd_4Zn_4		
**Atom**	**No**	**Charge**	**Atom**	**No**	**Charge**	**Atom**	**No**	**Charge**	**Atom**	**No**	**Charge**
Pd	1	0.00	Pd	1	-0.19	Pd	1	-0.32	Pd	1	-0.24
Pd	2	0.00	Pd	2	-0.19	Pd	2	-0.31	Pd	2	-0.33
Pd	3	0.11	Zn	3	1.01	Pd	3	-0.31	Pd	3	-0.55
Pd	4	0.11	Zn	4	0.23	Zn	4	0.37	Pd	4	-0.55
Pd	5	0.11	H	5	-0.43	Zn	5	1.06	Zn	5	0.59
Pd	6	0.11	H	6	-0.43	Zn	6	0.37	Zn	6	0.59
H	7	-0.22				H	7	-0.43	Zn	7	0.82
H	8	-0.22				H	8	-0.43	Zn	8	0.40
									H	9	-0.27
									H	10	-0.47

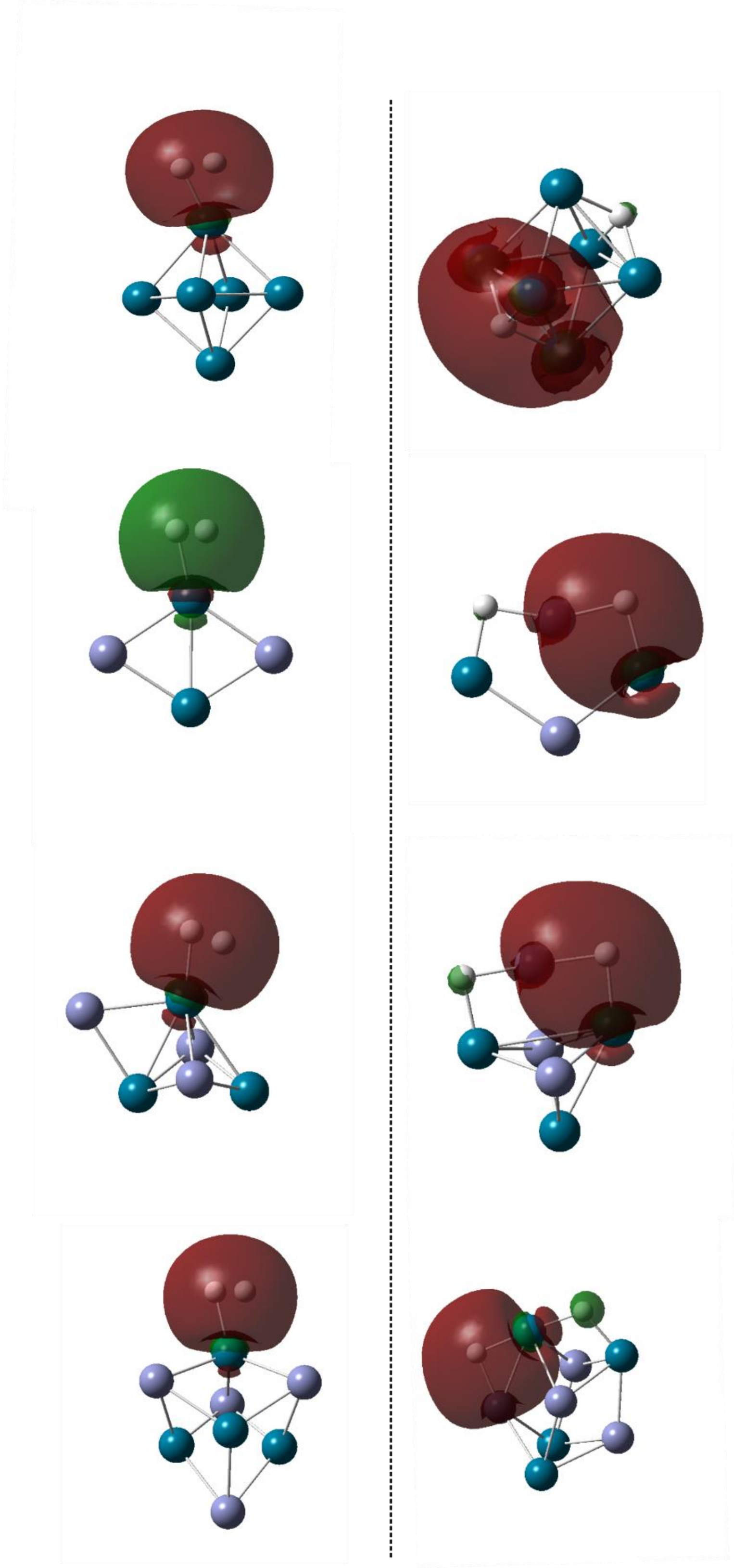

Figure S2. Localized molecular orbitals (LMOs) involving hydrogen for the intact (left panel) and dissociated (right panel) hydrogen adducts of Pd_6, Pd_2Zn_2, Pd_3Zn_3, and Pd_4Zn_4 clusters.

Table S10. Collected bond lengths (Å) electron densities at the bond critical points (BCP) and Laplacians (a.u.) for the Pd_6, Pd_2Zn_2, Pd_3Zn_3, Pd_4Zn_4 clusters.

Pd_6_cluster				
**Atom**	**Atom**	**Bond length (**	**rho (BCP)**	$\nabla^2\rho$
Pd1	Pd4	2.804	0.039	0.088
Pd1	Pd5	2.546	0.061	0.133
Pd1	Pd3	2.728	0.043	0.102
Pd1	Pd6	2.644	0.051	0.117
Pd2	Pd4	2.646	0.050	0.116
Pd2	Pd5	2.733	0.043	0.100
Pd2	Pd3	2.672	0.048	0.114
Pd2	Pd6	2.732	0.043	0.099
Pd3	Pd4	2.543	0.061	0.134
Pd3	Pd6	2.731	0.043	0.100
Pd4	Pd5	2.725	0.044	0.102
Pd5	Pd6	2.673	0.048	0.114

Pd_2Zn_2_cluster				
**Atom**	**Atom**	**Bond length**	**rho (BCP)**	$\nabla^2\rho$
Pd1	Pd2	2.716	0.047	0.096
Pd1	Zn3	2.461	0.052	0.129
Pd1	Zn4	2.461	0.052	0.129
Pd2	Zn3	2.461	0.052	0.129
Pd2	Zn4	2.461	0.052	0.129

Pd_3Zn_3 cluster				
**Atom**	**Atom**	**Bond length**	**rho (BCP)**	$\nabla^2\rho$
Pd1	Pd2	2.814	0.041	0.081
Pd1	Pd3	2.814	0.041	0.081
Pd2	Pd3	2.657	0.053	0.108
Pd1	Zn4	2.481	0.051	0.122
Pd1	Zn6	2.481	0.051	0.122
Pd2	Zn4	2.535	0.047	0.105
Pd2	Zn6	2.535	0.047	0.105
Pd2	Zn5	2.498	0.049	0.116
Pd3	Zn4	2.535	0.047	0.105
Pd3	Zn5	2.498	0.049	0.116
Pd3	Zn6	2.535	0.047	0.105

Pd_4Zn_4 cluster				
**Atom**	**Atom**	**Bond length**	**rho (BCP)**	$\nabla^2\rho$
Pd1	Pd2	2.856	0.038	0.076
Pd1	Pd3	2.856	0.038	0.076
Pd1	Pd4	2.856	0.038	0.076
Pd2	Pd3	2.856	0.038	0.076
Pd2	Pd4	2.856	0.038	0.076
Pd3	Pd4	2.856	0.038	0.076
Pd1	Zn6	2.519	0.047	0.107
Pd1	Zn5	2.519	0.047	0.107
Pd1	Zn7	2.519	0.047	0.107
Pd2	Zn5	2.519	0.047	0.107
Pd2	Zn6	2.519	0.047	0.107
Pd2	Zn8	2.519	0.047	0.107
Pd3	Zn5	2.519	0.047	0.107
Pd3	Zn7	2.519	0.047	0.107
Pd3	Zn8	2.519	0.047	0.107
Pd4	Zn6	2.519	0.047	0.107
Pd4	Zn7	2.519	0.047	0.107
Pd4	Zn8	2.519	0.047	0.107

Table S11. Collected bond lengths (Å) electron densities at the bond critical points (BCP) and Laplacians (a.u.) for the intact H_2 adducts of the Pd_6, Pd_2Zn_2, Pd_3Zn_3, Pd_4Zn_4 clusters.

**Pd_6_H_2 intact**				
**Atom**	**Atom**	**Bond length**	**rho (BCP)**	**$\nabla^2\rho$**
H7	H8	0.859	0.201	-0.606
H7	Pd1	1.695	0.111	0.366
Pd1	Pd4	2.754	0.041	0.098
Pd1	Pd5	2.778	0.039	0.090
Pd1	Pd3	2.776	0.038	0.092
Pd1	Pd6	2.764	0.040	0.090
Pd2	Pd4	2.726	0.044	0.100
Pd2	Pd5	2.680	0.048	0.107
Pd2	Pd3	2.630	0.052	0.117
Pd2	Pd6	2.668	0.049	0.110
Pd3	Pd4	2.699	0.046	0.106
Pd3	Pd6	2.542	0.061	0.137
Pd4	Pd5	2.526	0.063	0.138
Pd5	Pd6	2.692	0.047	0.108

**Pd_2Zn_2_H_2_intact**				
**Atom**	**Atom**	**Bond length**	**rho (BCP)**	**$\nabla^2\rho$**
H5	H6	0.835	0.212	-0.687
Pd1	H5	1.734	0.102	0.369
Pd1	Pd2	2.714	0.048	0.087
Pd1	Zn3	2.488	0.049	0.116
Pd1	Zn4	2.487	0.049	0.116
Pd2	Zn3	2.449	0.054	0.133
Pd2	Zn4	2.452	0.054	0.131

**Pd_3Zn_3 H_2 intact**				
**Atom**	**Atom**	**Bond length**	**rho (BCP)**	**$\nabla^2\rho$**
H7	H8	0.841	0.209	-0.670
Pd2	H8	1.729	0.103	0.368
Pd1	Pd2	2.833	0.039	0.076
Pd1	Pd3	2.777	0.043	0.088
Pd2	Pd3	2.662	0.054	0.098
Pd1	Zn4	2.479	0.052	0.122
Pd1	Zn6	2.479	0.052	0.122
Pd2	Zn4	2.553	0.045	0.098
Pd2	Zn6	2.553	0.045	0.098
Pd2	Zn5	2.517	0.047	0.107
Pd3	Zn4	2.529	0.048	0.106
Pd3	Zn5	2.471	0.052	0.125
Pd3	Zn6	2.529	0.048	0.106

**Pd_4Zn_4 H_2 intact**				
**Atom**	**Atom**	**Bond length**	**rho (BCP)**	**$\nabla^2\rho$**
H9	H10	0.829	0.213	-0.702
H9	Pd1	1.764	0.097	0.351
Pd1	Pd2	2.915	0.035	0.065
Pd1	Pd3	2.907	0.035	0.064
Pd1	Pd4	2.921	0.035	0.063
Pd2	Pd3	2.817	0.040	0.082
Pd2	Pd4	2.806	0.040	0.085
Pd3	Pd4	2.813	0.040	0.083
Pd1	Zn6	2.513	0.045	0.104
Pd1	Zn5	2.535	0.045	0.103
Pd1	Zn7	2.539	0.045	0.103
Pd2	Zn5	2.518	0.048	0.107
Pd2	Zn6	2.521	0.048	0.106
Pd2	Zn8	2.549	0.045	0.098
Pd3	Zn5	2.516	0.048	0.108
Pd3	Zn7	2.517	0.048	0.108
Pd3	Zn8	2.561	0.044	0.094
Pd4	Zn6	2.514	0.048	0.109
Pd4	Zn7	2.516	0.048	0.108
Pd4	Zn8	2.558	0.045	0.095

Table S12. Collected bond lengths (Å) electron densities at the bond critical points (BCP) and Laplacians (a.u.) for the dissociated H_2 adducts of the Pd_6,Pd_2Zn_2, Pd_3Zn_3, Pd_4Zn_4 clusters.

**Pd_6_H_2 dissociated**				
**Atom**	**Atom**	**Bond length**	**rho (BCP)**	$\nabla^2\rho$
Pd1	Pd5	2.804	0.038	0.088
Pd1	Pd6	2.793	0.039	0.090
Pd2	Pd3	2.804	0.038	0.088
Pd2	Pd4	2.793	0.039	0.090
Pd3	Pd6	2.594	0.055	0.121
Pd4	Pd5	2.594	0.055	0.121
Pd1	H7	1.717	0.099	0.122
Pd3	H7	1.792	0.085	0.134
Pd4	H7	1.788	0.086	0.134
Pd2	H8	1.717	0.099	0.122
Pd5	H8	1.792	0.085	0.134
Pd6	H8	1.788	0.086	0.134

**Pd_2Zn_2_H_2 dissociated**				
**Atom**	**Atom**	**Bond length**	**rho (BCP)**	$\nabla^2\rho$
Pd1	Zn4	2.521	0.049	0.106
Pd2	Zn4	2.521	0.049	0.106
Pd1	H5	1.694	0.105	0.156
Pd2	H6	1.695	0.105	0.156
Zn3	H5	1.705	0.081	0.155
Zn3	H6	1.704	0.081	0.155

**Pd_3Zn_3 H_2 dissociated**				
**Atom**	**Atom**	**Bond length**	**rho (BCP)**	$\nabla^2\rho$
Pd1	Pd2	2.790	0.042	0.081
Pd1	Pd3	2.790	0.042	0.081
Pd1	Zn4	2.488	0.051	0.119
Pd1	Zn6	2.488	0.051	0.119
Pd2	Zn4	2.535	0.047	0.106
Pd2	Zn6	2.535	0.047	0.106
Pd3	Zn4	2.535	0.047	0.106
Pd3	Zn6	2.535	0.047	0.106
Pd2	H8	1.746	0.094	0.163
Pd3	H7	1.746	0.094	0.163
Zn5	H7	1.722	0.078	0.148
Zn5	H8	1.722	0.078	0.148

**Pd_3Zn_3 H_2 dissociated**				
**Atom**	**Atom**	**Bond length**	**rho (BCP)**	$\nabla^2\rho$
Pd3	Pd4	2.783	0.042	0.089
Pd1	Zn6	2.540	0.046	0.092
Pd1	Zn5	2.565	0.046	0.092
Pd2	Zn5	2.481	0.053	0.120
Pd2	Zn6	2.480	0.053	0.121
Pd2	Zn8	2.519	0.050	0.099
Pd3	Zn5	2.446	0.057	0.130
Pd3	Zn7	2.499	0.053	0.105
Pd3	Zn8	2.607	0.043	0.083
Pd4	Zn6	2.446	0.057	0.130
Pd4	Zn7	2.499	0.053	0.105
Pd4	Zn8	2.607	0.043	0.083
Pd1	H9	1.722	0.099	0.131
Pd1	H10	1.722	0.098	0.175
Pd2	H9	1.734	0.095	0.144
Zn7	H10	1.778	0.069	0.149

Ensemble of structures in the CO_2 hydrogenation on palladium-zinc clusters

Similarly to the H_2 adducts of Pd_6, the CO_2 adducts of Pd_6H_2 show a significant ensemble effect, which is well visible by the several possible symmetry-equivalent isomers of the structures shown in Figure S3. This implies several possible binding sites for CO_2, followed by possible activation routes. This again leads to an entropy-driven stabilization of the carbon dioxide binding and activation.

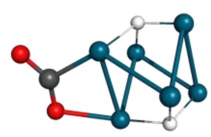	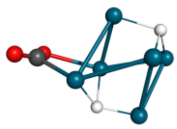	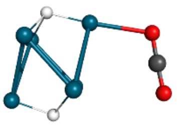	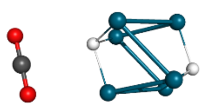
0<br>a	21.7<br>b	22.2<br>c	32<br>d

Figure S3. Geometries of the most stable CO_2 adducts of Pd_6H_2. Relative energies compared to the lowest energy isomers are indicated in kJ/mol.

However, the case is different for the carbon dioxide binding to Pd_3Zn_3 (Figure S4). In the three low-lying isomers CO_2 prefer to bound as a van der Waals complex, thus the ensemble effects have no implications on the carbon dioxide activation or dissociation.

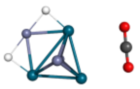<br>0<br>a	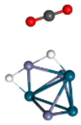<br>4<br>b	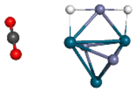<br>7<br>c

Figure S4. Geometries of the most stable CO_2 adducts of $Pd_3Zn_3H_2$. Relative energies compared to the lowest energy isomer are indicated in kJ/mol.

Physisorbed CO_2 adducts			Activated CO_2 adducts		
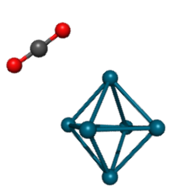	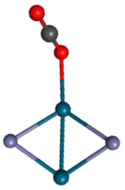	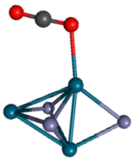	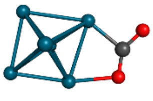	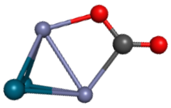	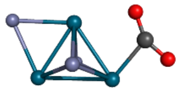
-21	-21	-24	-64	+82	-13

Figure S5. CO_2 adducts for Pd_6, Pd_2Zn_2 and Pd_3Zn_3. Relative energies compared to the lowest energy isomer are free CO_2 indicated in kJ/mol.

HCOO and COOH formation on the studied clusters

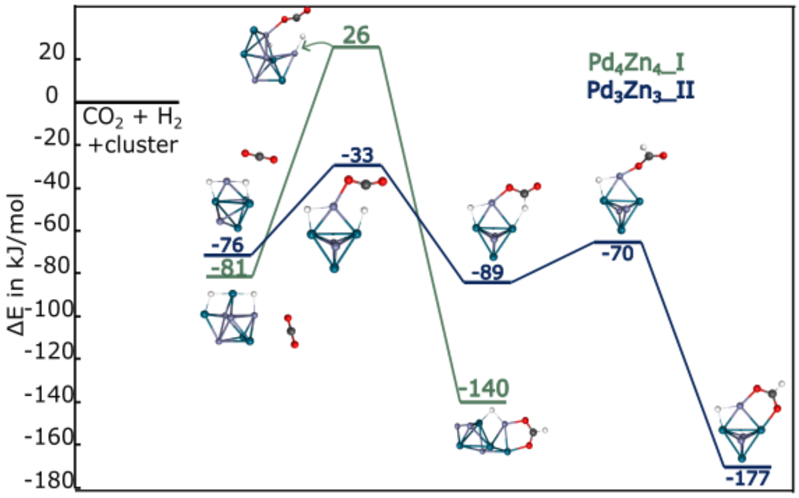

Figure S6. Alternative H_2 dissociation route for the CO_2 hydrogenation reaction on Pd_4Zn_4_I and Pd_3Zn_3_II towards formate. H, C, and O atoms are depicted as white, black, and red spheres, respectively. Energies are included in kJ/mol.

Table S13. Basis-set superposition error (BSSE) of the CO_2 adducts of Pd_6 and Pd_3Zn_3 (from Figure S5). Basis-set superposition error (BSSE) is the difference between the uncorrected and the counterpoise corrected binding energies.

Adduct	BSSE, kJ/mol
Pd_6-CO_2 (Physisorbed CO_2)	1
Pd_6-CO_2 (Activated CO_2)	3
Pd_3Zn_3-CO_2 (Physisorbed CO_2)	2
Pd_3Zn_3-CO_2 (Activated CO_2)	3

Table S14. Total natural atomic charges of the COOH and HCOO groups (from Figure 4) adsorbed on gas-phase Pd_6, Pd_2Zn_2, Pd_3Zn_3 and Pd_4Zn_4 clusters.

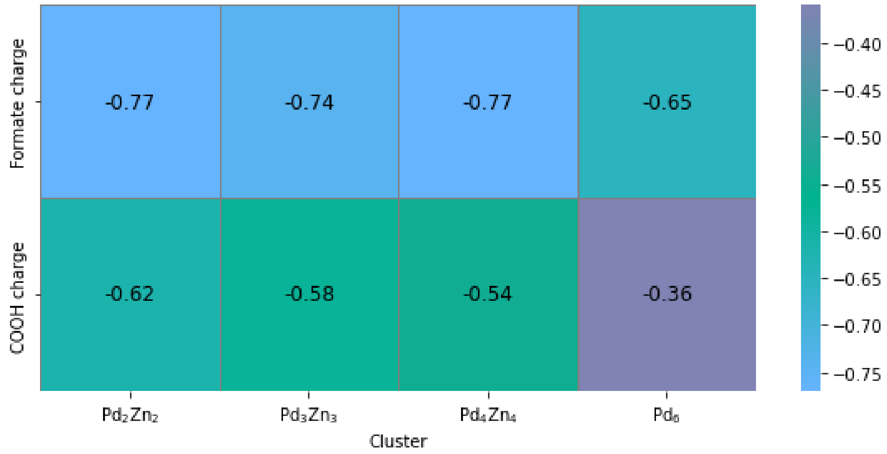

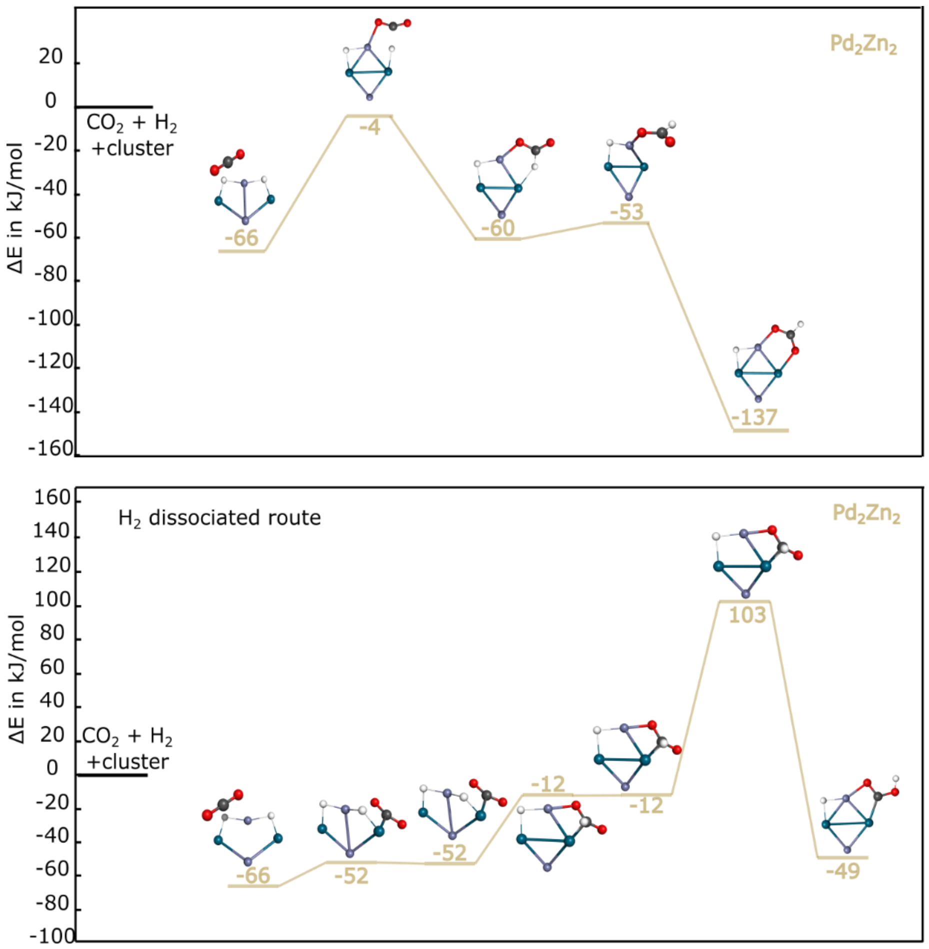

Figure S7. Dissociated H_2 (HD) route for the CO_2 hydrogenation to formate (upper panel) and carboxylate (lower panel) on Pd_2Zn_2. H, C, and O atoms are depicted as white, black, and red spheres, respectively. Energies are included in kJ/mol.

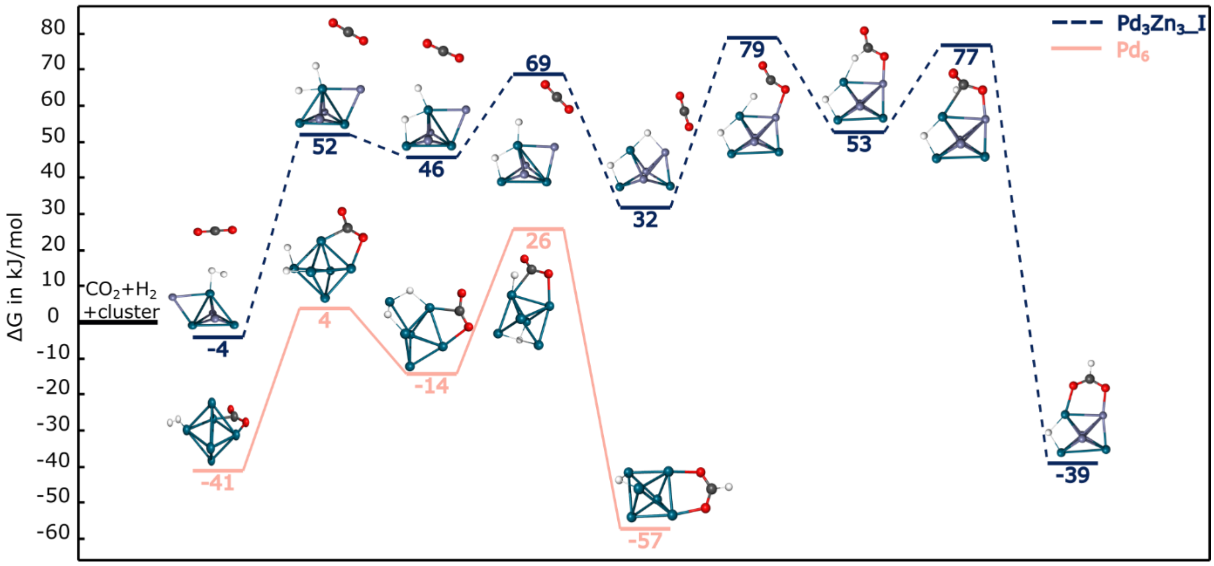

Figure S8. Gibbs free energy profiles at 298K for the intact H_2 (IH) route for the CO_2 hydrogenation to formate on Pd_6 and Pd_3Zn_3_II (from Figure 5). H, C, and O atoms are depicted as white, black, and red spheres, respectively.

Projected density of states (PDOS)

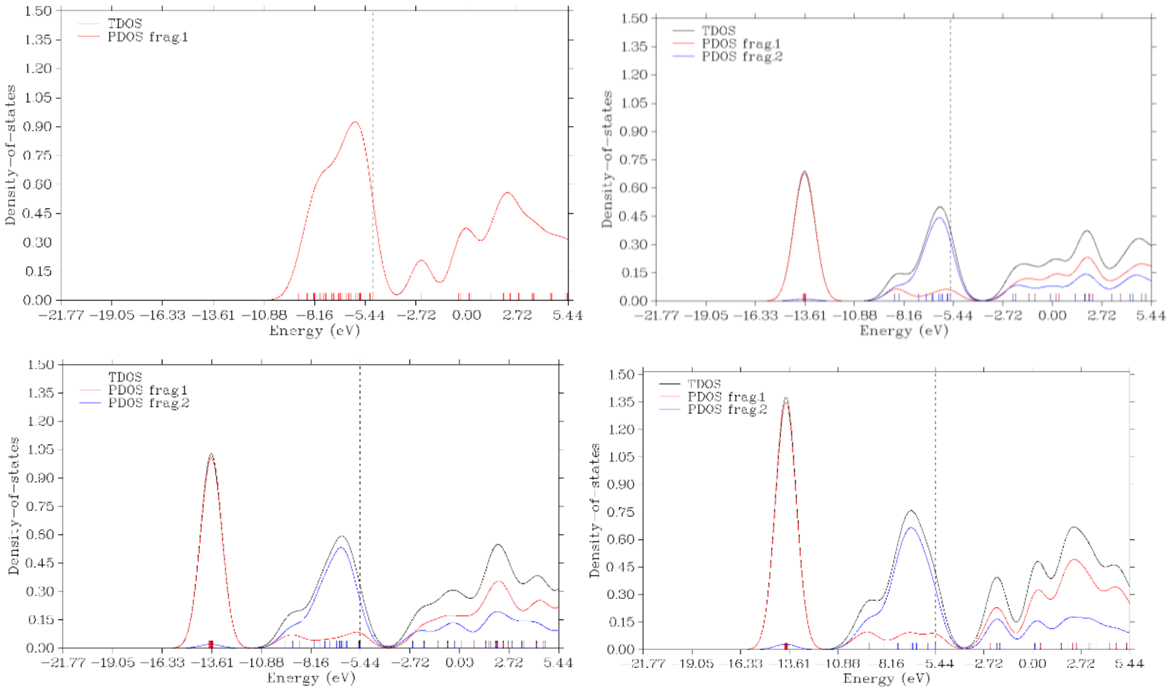

Figure S9. Projected density of states (PDOS) (panels arranged left-to-right, top-to-bottom) for: Pd_6, Pd_2Zn_2, Pd_3Zn_3, and Pd_4Zn_4. For the PdZn clusters, fragment 1 is composed of Zn atoms and fragment 2 is composed of Pd atoms.

Table S15. Zn and Pd *d*-band centres for the bare clusters.

	d-band centres	
Cluster	Zn (eV)	Pd (eV)
Pd_2Zn_2	0.21	2.58
Pd_3Zn_3	-0.01	1.89
Pd_4Zn_4	0.12	1.30
Pd_6	---	2.38

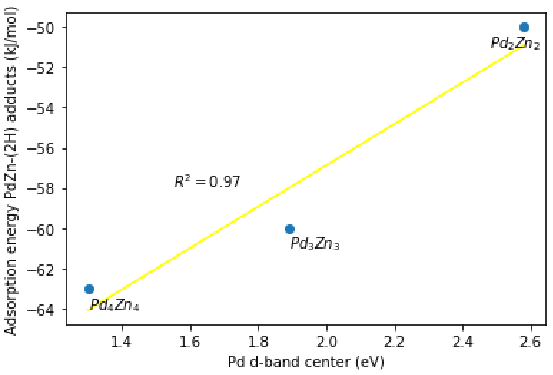

Figure S10. Correlation between the adsorption energy of PdZn(2H) adducts and the Pd d-band center.

Cartesian coordinates (in Å)

Table S16. Cartesian coordinates and SCF energies of neutral bare clusters from Table S8.

Cluster	Isomers			
Pd_6	Pd -0.093560 -1.318570 1.377140<br>Pd 0.008510 1.280860 -1.432710<br>Pd 1.858870 0.048240 0.049010<br>Pd 0.098950 1.473770 1.205140<br>Pd -1.859290 -0.038750 0.063730<br>Pd -0.013480 -1.445560 -1.262310<br>SCF energy (Eh) = -767,491301 (M=3)<br>SCF energy (Eh) = -767,4742385 (M=1)<br>SCF energy (Eh) = -767,4663479 (M=5)		Pd -0.001528983 -0.110947190 1.397037938<br>Pd 1.853378199 -1.236082085 0.117067492<br>Pd 0.002787779 -0.415631586 -1.521607128<br>Pd -1.360236460 1.502824913 -0.054342431<br>Pd -1.842978869 -1.254132018 0.112649408<br>Pd 1.348578433 1.513967966 -0.050805279<br>SCF energy (Eh) = -767,4576099 (M=1)	
Pd_2Zn_2	Pd -0.000002 1.358199 0.000022<br>Pd 0.000002 -1.358200 0.000022<br>Zn -2.051760 -0.000007 -0.000046<br>Zn 2.051754 -0.000001 -0.000046<br>SCF energy (Eh) = -3814,173593 (M=1)			
Pd_3Zn_3	Pd -2.011530 -0.000010 0.000000<br>Pd 0.469270 0.000000 -1.328530<br>Pd 0.469270 0.000000 1.328530<br>Zn -0.469880 -1.943460 0.000000<br>Zn 2.585020 0.000000 0.000000<br>Zn -0.469890 1.943460 0.000000<br>SCF energy (Eh) = -5721.320590 (M=1)		Pd 2.276738187 0.765318140 -0.007706132<br>Pd -2.276712867 0.765355613 -0.007727372<br>Pd 0.000008869 -0.504501284 0.911552907<br>Zn 1.697084788 -1.585181634 -0.460611676<br>Zn 0.000031572 1.587956558 -0.449638690<br>Zn -1.697151849 -1.585166294 -0.460474737<br>SCF energy (Eh) = -5721,302677 (M=1)	
Pd_4Zn_4	Pd 1.014310 -1.007580 -1.007790<br>Pd -1.009450 -1.012210 1.008020<br>Pd 1.005380 1.012430 1.011860<br>Pd -1.010240 1.007360 -1.012080<br>Zn 1.436870 -1.432670 1.439220<br>Zn -1.430000 -1.439840 -1.438910<br>Zn 1.435780 1.439540 -1.433430<br>Zn -1.442650 1.432970 1.433120<br>SCF energy (Eh) = -7628.474500 (M=1)<br>SCF energy (Eh) = -7628,417904 (M=3)	Pd -0.204864477 0.493086029 0.938054974<br>Pd 2.147090998 1.229358729 -0.441209203<br>Pd 1.031913939 -1.351445995 -0.636067293<br>Pd -2.094392323 -0.305617571 -0.834047574<br>Zn -2.493668636 1.438806692 0.946274398<br>Zn 2.092245002 -0.372410514 1.459526805<br>Zn 0.026136710 0.749793190 -1.628735319<br>Zn -0.970511603 -1.938933411 0.704151899<br>SCF energy (Eh) = -7628,468862 (M=1)<br>SCF energy (Eh) =-7628,435047 (M=3)	Pd 0.004838026 2.372613160 -0.003985855<br>Pd -0.002909710 -1.416820809 0.008948719<br>Pd -1.931006317 0.468680004 -0.000048962<br>Pd 1.932600862 0.460864102 -0.002256105<br>Zn 0.001633205 0.495722865 1.708325398<br>Zn -0.000013463 0.486088356 -1.706375813<br>Zn -2.426678913 -1.941993387 -0.003515917<br>Zn 2.419073110 -1.951429555 -0.003084641<br>SCF energy (Eh) = -7628,466275 (M=1)<br>SCF energy (Eh) = -7628,432322 (M=3)	Pd 0.168138728 -0.835248605 -0.561366404<br>Pd -2.064284735 0.094500221 0.622027502<br>Pd -0.172664958 1.987821124 -0.240614875<br>Pd 2.261283354 0.662078413 0.354561004<br>Zn 0.194084010 0.398807696 1.689888509<br>Zn -1.494472447 0.591028605 -1.821459487<br>Zn 2.495106366 -1.605019432 -0.619119853<br>Zn -1.494614221 -2.330384849 0.478621751<br>SCF energy (Eh) = -7628,4596 (M=1)

Table S17. Cartesian coordinates and SCF energies of adducts from Figure 2.

Cluster	Intact H_2 adducts	Dissociated H_2 adducts		
Pd_6	Pd 1.672101 0.019822 1.214935<br>Pd -1.672097 -0.019823 -1.214938<br>Pd -0.734022 1.390299 1.019688<br>Pd -0.765233 -1.302212 1.094982<br>Pd 0.734029 -1.390299 -1.019693<br>Pd 0.765235 1.302212 -1.094983<br>H 0.090984 0.055366 1.884003<br>H -0.090982 -0.055373 -1.884005<br>SCF energy (Eh) = -768.685960 (M=3)	Pd 1.9998017 0.2127965 -0.0499534<br>Pd -1.9732265 -0.1692007 0.0518582<br>Pd -0.1667878 1.2255171 1.3591033<br>Pd 0.1651680 -1.4456813 1.1605544<br>Pd 0.0341012 -1.2533607 -1.3546960<br>Pd -0.2166100 1.4204490 -1.1751558<br>H 3.5596530 0.2787868 0.6111513<br>H 3.6878062 0.1573002 -0.2298445<br>SCF energy (Eh) = -768.694756 (M=3)	Pd 0.452396 1.887613 0.923544<br>Pd -0.322586 -1.438457 -0.830996<br>Pd -0.044014 -0.622133 1.718699<br>Pd 2.077020 -0.156940 -0.058007<br>Pd 0.070179 1.125546 -1.468608<br>Pd -2.017527 0.621370 0.162734<br>H 1.432819 0.304577 1.428595<br>H -1.467840 -0.951351 0.350044<br>SCF energy (Eh) = -768.690540 (M=3)	Pd 0.149646 1.695399 0.915857<br>Pd -0.108774 -1.567006 -0.966062<br>Pd -0.141560 -0.933364 1.696676<br>Pd 2.085063 -0.242253 -0.098266<br>Pd 0.162199 0.834755 -1.675591<br>Pd -1.990818 0.225486 -0.036508<br>H -1.192463 0.528589 1.454783<br>H 1.089674 0.220737 1.158944<br>SCF energy (Eh) = -768.687858 (M=3)
Pd_2Zn_2	Pd -0.0055092 1.3304835 0.0120911<br>Pd 0.0066163 -1.3831070 0.0123670<br>Zn 2.0621268 -0.0529209 -0.0174632<br>Zn -2.0623058 -0.0673506 -0.0198222<br>H -0.3885907 3.0111183 0.1975237<br>H 0.3430342 3.0177115 -0.2040344<br>SCF energy (Eh) = -3815.367595 (M=1)		Pd 2.0473330 0.0090716 -0.0000867<br>Pd -2.0469413 0.0089177 -0.0006593<br>Zn -0.0005002 1.3239909 -0.0006195<br>Zn -0.0001260 -1.4623020 0.0006720<br>H 1.6707503 1.6604375 0.0002246<br>H -1.6699818 1.6613896 0.0325130<br>SCF energy (Eh) = -3815.360670 (M=1)	
Pd_3Zn_3	a.<br>Pd -2.006410125 0.000011669 -0.009213192<br>Pd 0.502416448 0.000479939 -1.319170271<br>Pd 0.425813483 -0.000332422 1.342038572<br>Zn -0.475176836 -1.949384159 0.009023310<br>Zn 2.570403907 -0.000009544 0.114171899<br>Zn -0.475257839 1.949479091 0.010236379<br>H 1.160606723 0.001055841 -2.921065966<br>H 0.327089776 0.000551508 -3.039827483<br>SCF energy (Eh) = -5722.517702 (M=1)<br>b.<br>Pd -2.032401 0.000700 -0.015261<br>Pd 0.477898 0.001391 -1.304836<br>Pd 0.525225 -0.000440 1.319284<br>Zn -0.443309 -1.943999 0.018026<br>Zn 2.662549 0.000377 -0.024311<br>Zn -0.442884 1.945147 0.020809<br>H -3.691688 -0.413515 0.434849<br>H -3.693845 0.411120 0.432206<br>SCF energy (Eh) = -5722.507261 (M=1)	Pd 1.898448388 0.000027769 0.000770364<br>Pd -0.371047490 1.631904922 -0.001146287<br>Pd -0.371005864 -1.631920650 0.001598901<br>Zn 0.234654240 0.001543951 1.844603411<br>Zn -2.141151241 -0.000035128 -0.000274948<br>Zn 0.235541015 -0.001546304 -1.843919637<br>H -2.113806881 -1.722961180 0.000866439<br>H -2.113893443 1.722875211 -0.001537515<br>SCF energy (Eh) = -5722.511636 (M=1)	Pd 1.649260 -1.271649 0.000089<br>Pd 1.146314 1.396895 0.000570<br>Pd -1.933998 -0.819859 0.000092<br>Zn -0.047607 -0.252532 1.592730<br>Zn -1.283450 1.482342 0.000251<br>Zn -0.047079 -0.252762 -1.592723<br>H 2.395225 0.204999 0.005358<br>H -0.007909 2.698949 -0.002064<br>SCF energy (Eh) = -5722.500388 (M=1)	Pd 2.358815419 0.002374835 0.113364150<br>Pd 0.173487666 1.430941761 -0.458116238<br>Pd -1.521094748 -1.310688502 0.127752807<br>Zn 0.278342676 -0.244082562 1.395321148<br>Zn -2.207416945 1.103629732 0.007875129<br>Zn 0.456727130 -0.921576862 -1.441209910<br>H 1.463956402 1.317500394 0.774830837<br>H -0.878744241 -1.465422076 1.730231673<br>SCF energy (Eh) = -5722.499654 (M=1)<br>Pd -1.735235171 -0.799369721 0.412496462<br>Pd 0.019801873 -0.454417622 -1.672003210<br>Pd 1.756879394 0.777697168 0.046172066<br>Zn 0.008260092 0.396962226 1.970314430<br>Zn 0.732034924 -1.577417505 0.373275804<br>Zn -0.706006527 1.458742208 -0.340266117<br>H -1.565269443 -1.057445623 -1.286921273<br>H 1.605473613 0.267175689 -1.598177538<br>SCF energy (Eh) = -5722.498515 (M=1)
Pd_4Zn_4	Pd 1.807188815 0.036833194 -0.006766974<br>Pd -0.637394064 1.554857989 -0.448217681<br>Pd -0.611619174 -1.170491091 -1.125487246<br>Pd -0.597387179 -0.404289080 1.577024391<br>Zn 0.796727391 0.574487045 -2.268740600<br>Zn 0.788097559 1.694424660 1.622408510<br>Zn 0.852067669 -2.227188662 0.629101409<br>Zn -2.586954751 -0.026508260 0.019700856<br>H 3.517656046 0.339168527 -0.331282454<br>H 3.531413854 -0.231095524 0.268543909<br>SCF energy (Eh) = -7629.661800 (M=1)	Pd -2.320218115 0.024633269 -0.165383894<br>Pd -0.445550661 -0.007951195 -2.236077730<br>Pd 1.378067085 1.379461262 1.262144123<br>Pd 1.346024618 -1.393956679 1.280221590<br>Zn -0.379019893 1.680383833 -0.414454400<br>Zn -0.415168527 -1.675389362 -0.395765416<br>Zn -0.659261305 0.019888312 1.743182431<br>Zn 1.704479528 -0.023997197 -0.919737628<br>H -2.134247667 0.011638681 -1.873027513<br>H -2.437736050 0.040050728 1.551953599<br>SCF energy (Eh) = -7629.666720 (M=1)	Pd 1.862128 -0.929617 1.031322<br>Pd 1.863527 0.926324 -1.025481<br>Pd -1.863316 -1.026410 -0.925162<br>Pd -1.861891 1.030384 0.930804<br>Zn 0.666819 -1.277362 -1.135206<br>Zn 0.668982 1.276076 1.141095<br>Zn -0.668706 -1.137176 1.279651<br>Zn -0.666641 1.139081 -1.273805<br>H 2.925963 -0.002294 0.002972<br>H -2.925714 0.002521 0.002846<br>SCF energy (Eh) = -7629.653527 (M=1)	Pd 1.841934 0.888299 0.041939<br>Pd -1.839654 0.892675 0.040760<br>Pd 0.000024 -0.733420 -1.463312<br>Pd -0.001444 -1.100168 1.283411<br>Zn 0.002852 1.928120 -1.189679<br>Zn 0.001034 1.312651 1.782804<br>Zn 1.966192 -1.627258 -0.169624<br>Zn -1.969108 -1.622411 -0.171194<br>H 1.709477 2.160634 -1.142984<br>H -1.702437 2.167522 -1.141696<br>SCF energy (Eh) = -7629.651768 (M=1)

Table S18. Cartesian coordinates and SCF energies of adducts from Figure 3, reaction paths for H_2 dissociation.

Cluster	H_2 adducts		
Pd_6	Pd 2.0051832 0.1578553 -0.0432710<br>Pd -1.9772904 -0.1133280 0.0439333<br>Pd -0.1366350 1.2205348 1.3668641<br>Pd 0.1209789 -1.4573883 1.1494238<br>Pd 0.0033200 -1.2429698 -1.3647025<br>Pd -0.1729483 1.4354253 -1.1660104<br>H 3.5641690 0.1752167 0.6229212<br>H 3.6915316 0.0563746 -0.2185725<br>SCF energy (Eh) = -768.685960 (M=3)	Pd 2.290436 0.037858 -0.503425<br>Pd -1.643094 0.036546 0.151348<br>Pd 0.626538 1.344233 1.201698<br>Pd 0.655776 -1.328123 1.006750<br>Pd -0.075683 -1.262063 -1.502862<br>Pd -0.102758 1.298151 -1.479124<br>H 2.216889 1.075991 0.795936<br>H 2.922986 -0.784464 0.649658<br>SCF energy (Eh) = -768.669652 (M=3)	Pd 0.149646 1.695399 0.915857<br>Pd -0.108774 -1.567006 -0.966062<br>Pd -0.141560 -0.933364 1.696676<br>Pd 2.085063 -0.242253 -0.098266<br>Pd 0.162199 0.834755 -1.675591<br>Pd -1.990818 0.225486 -0.036508<br>H -1.192463 0.528589 1.454783<br>H 1.089674 0.220737 1.158944<br>SCF energy (Eh) = -768.687858 (M=3)
	Pd 0.187715 1.664439 0.866150<br>Pd -0.060301 -1.423754 -1.058943<br>Pd -0.139393 -0.945470 1.600922<br>Pd 2.172259 -0.222581 -0.079783<br>Pd 0.348202 0.987391 -1.694551<br>Pd -1.960525 0.278789 -0.097408<br>H 1.122895 0.220299 1.139223<br>H -1.451597 0.196795 1.495906<br>SCF energy (Eh) = -768.686761(M=3)	Pd 0.452396 1.887613 0.923544<br>Pd -0.322586 -1.438457 -0.830996<br>Pd -0.044014 -0.622133 1.718699<br>Pd 2.077020 -0.156940 -0.058007<br>Pd 0.070179 1.125546 -1.468608<br>Pd -2.017527 0.621370 0.162734<br>H 1.432819 0.304577 1.428595<br>H -1.467840 -0.951351 0.350044<br>SCF energy (Eh) = -768.690540 (M=3)	Pd 0.357096 1.718019 1.118428<br>Pd -0.222259 -1.200980 -1.090925<br>Pd -0.127378 -0.912117 1.527698<br>Pd 2.179799 -0.119504 -0.006488<br>Pd 0.357471 1.236275 -1.479190<br>Pd -1.954007 0.586975 0.061968<br>H 1.340672 0.303441 1.372694<br>H -1.691890 -0.880862 0.802635<br>SCF energy (Eh) = -768.688525(M=3)
		Pd 1.9998017 0.2127965 -0.0499534<br>Pd -1.9732265 -0.1692007 0.0518582<br>Pd -0.1667878 1.2255171 1.3591033<br>Pd 0.1651680 -1.4456813 1.1605544<br>Pd 0.0341012 -1.2533607 -1.3546960<br>Pd -0.2166100 1.4204490 -1.1751558<br>H 3.5596530 0.2787868 0.6111513<br>H 3.6878062 0.1573002 -0.2298445<br>SCF energy (Eh) = -768.694756 (M=3)	
Pd_2Zn_2	Pd -0.0055092 1.3304835 0.0120911<br>Pd 0.0066163 -1.3831070 0.0123670<br>Zn 2.0621268 -0.0529209 -0.0174632<br>Zn -2.0623058 -0.0673506 -0.0198222<br>H -0.3885907 3.0111183 0.1975237<br>H 0.3430342 3.0177115 -0.2040344<br>SCF energy (Eh) = -3815.367595 (M=1)	Pd -0.019142 1.388018 0.079042<br>Pd 0.019469 -1.394319 0.127391<br>Zn -2.042621 -0.066051 -0.192461<br>Zn 2.050267 -0.019435 -0.188637<br>H -0.134300 2.765914 0.919216<br>H -0.110129 0.088517 1.017836<br>SCF energy (Eh) = -3815.323018	Pd -0.012013 1.021066 0.913507<br>Pd -0.142487 -1.468849 -0.339035<br>Zn -1.973634 0.312565 -0.295813<br>Zn 1.828143 0.166047 -0.393469<br>H 0.032120 2.594553 0.668004<br>H -0.072610 -0.751870 1.141368<br>SCF energy (Eh) = -3815.338792
	Pd 0.244967 1.312882 0.913767<br>Pd 0.084342 -1.513080 -0.275308<br>Zn -1.632584 0.244130 -0.013640<br>Zn 1.890493 0.253114 -0.602119<br>H -0.162054 2.762882 0.543108<br>H -0.807215 -1.208858 0.988227<br>SCF energy (Eh) = -3815.324772	Pd 2.0473330 0.0090716 -0.0000867<br>Pd -2.0469413 0.0089177 -0.0006593<br>Zn -0.0005002 1.3239909 -0.0006195<br>Zn -0.0001260 -1.4623020 0.0006720<br>H 1.6707503 1.6604375 0.0002246<br>H -1.6699818 1.6613896 0.0325130<br>SCF energy (Eh) = -3815.360670 (M=1)	
Pd_3Zn_3_I	Pd -2.006410125 0.000011669 -0.009213192<br>Pd 0.502416448 0.000479939 -1.319170271<br>Pd 0.425813483 -0.000332422 1.342038572<br>Zn -0.475176836 -1.949384159 0.009023310<br>Zn 2.570403907 -0.000009544 0.114171899<br>Zn -0.475257839 1.949479091 0.010236379<br>H 1.160606723 0.001055841 -2.921065966<br>H 0.327089776 0.000551508 -3.039827483<br>SCF energy (Eh) = -5722.517702 (M=1)	Pd -2.154040 -0.025860 -0.099830<br>Pd 0.223450 -0.007620 -1.288400<br>Pd 0.912080 0.037280 1.322590<br>Zn -0.472300 -1.828710 0.436160<br>Zn 2.597750 -0.019270 -0.551380<br>Zn -0.515760 1.846430 0.369950<br>H -0.135440 -0.046890 -2.817630<br>H -1.303960 -0.081030 -1.805210<br>SCF energy (Eh) = -5722.490530 (M=1)	Pd -2.040920 -0.008580 -0.028440<br>Pd 0.342970 0.010270 -1.362440<br>Pd 0.590240 -0.003010 1.397500<br>Zn -0.389010 -1.920220 0.167380<br>Zn 2.529440 0.004310 -0.208130<br>Zn -0.412220 1.919870 0.181210<br>H 0.409470 0.025780 -2.928940<br>H -1.300910 -0.084070 -1.589100<br>SCF energy (Eh) = -5722.492296 (M=1)
	SCF energy (Eh) = -5722.484853 (M=1)	Pd 1.68153 -1.21528 0.00970<br>Pd 1.08967 1.43886 -0.01098<br>Pd -1.89627 -0.89850 -0.00665<br>Zn -0.04142 -0.24334 1.59419<br>Zn -1.33877 1.43108 0.01455<br>Zn -0.03705 -0.25259 -1.59562<br>H -0.10461 2.70474 -0.03154<br>H 2.37478 0.28705 0.00331<br>SCF energy (Eh) = -5722.500400 (M=1)	
Pd_3Zn_3_II	Pd -2.006410125 0.000011669 -0.009213192<br>Pd 0.502416448 0.000479939 -1.319170271<br>Pd 0.425813483 -0.000332422 1.342038572<br>Zn -0.475176836 -1.949384159 0.009023310<br>Zn 2.570403907 -0.000009544 0.114171899<br>Zn -0.475257839 1.949479091 0.010236379<br>H 1.160606723 0.001055841 -2.921065966<br>H 0.327089776 0.000551508 -3.039827483<br>SCF energy (Eh) = -5722.517702 (M=1)	Pd -2.103981 -0.007602 0.308948<br>Pd -0.285491 -0.003528 -1.636738<br>Pd 0.474871 0.004441 1.189347<br>Zn -0.527998 -1.946968 -0.091799<br>Zn 1.996549 0.013485 -0.706552<br>Zn -0.543279 1.942390 -0.098818<br>H 3.410181 0.021123 -1.344179<br>H -0.820672 -0.004230 -3.140253<br>SCF energy (Eh) = -5722.463464 (M=1)	Pd 1.904579 0.000001 0.000012<br>Pd -0.364486 1.623981 0.000001<br>Pd -0.364482 -1.623981 -0.000010<br>Zn 0.240481 -0.000010 1.849837<br>Zn -2.143051 -0.000003 -0.000002<br>Zn 0.240505 0.000012 -1.849839<br>H -2.108067 -1.721547 -0.000108<br>H -2.108072 1.721542 0.000102<br>SCF energy (Eh) = -5722.511627 (M=1)
Pd_4Zn_4_I	Pd 1.807188815 0.036833194 -0.006766974<br>Pd -0.637394064 1.554857989 -0.448217681<br>Pd -0.611619174 -1.170491091 -1.125487246<br>Pd -0.597387179 -0.404289080 1.577024391<br>Zn 0.796727391 0.574487045 -2.268740600<br>Zn 0.788097559 1.694424660 1.622408510<br>Zn 0.852067669 -2.227188662 0.629101409<br>Zn -2.586954751 -0.026508260 0.019700856<br>H 3.517656046 0.339168527 -0.331282454<br>H 3.531413854 -0.231095524 0.268543909<br>SCF energy (Eh) = -7629.661800 (M=1)	Pd -2.652144312 0.070686918 -0.142330577<br>Pd 0.467246599 -0.409179562 -1.472395627<br>Pd 1.173422261 1.540867459 0.583109391<br>Pd 1.088889885 -1.191521936 1.026771819<br>Zn -0.865660767 1.595726205 -0.794064756<br>Zn -1.045172079 -1.913186113 -0.155053522<br>Zn -0.849907374 0.341594551 1.553503932<br>Zn 2.851659271 -0.144413102 -0.634543192<br>H -2.343245826 0.854711830 -1.622900711<br>H -2.653768706 0.182737249 1.602330347<br>SCF energy (Eh) = -7629.628800	Pd 1.874179 -1.392334 0.002951<br>Pd 1.821792 1.394346 -0.001941<br>Pd -1.842976 -0.040365 -1.391710<br>Pd -1.844330 -0.033724 1.390884<br>Zn 0.584521 0.050282 -1.680553<br>Zn 0.583021 0.057654 1.680667<br>Zn -0.642878 -1.730021 0.004099<br>Zn -0.660718 1.823821 -0.004549<br>H 2.868973 0.012770 -0.006763<br>H 0.814064 -2.749299 0.008374<br>SCF energy (Eh) = -7629.666600
Pd_4Zn_4_II	Pd 1.807188815 0.036833194 -0.006766974<br>Pd -0.637394064 1.554857989 -0.448217681<br>Pd -0.611619174 -1.170491091 -1.125487246<br>Pd -0.597387179 -0.404289080 1.577024391<br>Zn 0.796727391 0.574487045 -2.268740600<br>Zn 0.788097559 1.694424660 1.622408510<br>Zn 0.852067669 -2.227188662 0.629101409<br>Zn -2.586954751 -0.026508260 0.019700856<br>H 3.517656046 0.339168527 -0.331282454<br>H 3.531413854 -0.231095524 0.268543909<br>SCF energy (Eh) = -7629.661800 (M=1)	Pd -1.467130409 -0.823967441 0.313440114<br>Pd 0.000613898 0.354644009 -1.942888372<br>Pd -0.774396497 0.992645359 2.045426356<br>Pd 0.999444237 -1.826719776 -0.229260087<br>Zn -1.588964074 1.560980750 -0.378981674<br>Zn -0.946684450 -1.957009685 -1.827823631<br>Zn -0.024843243 -1.782137816 2.123800668<br>Zn 1.033628352 0.596827439 0.328376875<br>H -2.284496099 0.582791899 1.096311533<br>H -1.513250616 2.333932061 -1.765949982<br>SCF energy (Eh) = -7629,644732 (M=1)	Pd 1.841933674 0.888298949 0.041938711<br>Pd -1.839653532 0.892675289 0.040760098<br>Pd 0.000023943 -0.733420001 -1.463311890<br>Pd -0.001443619 -1.100168468 1.283410896<br>Zn 0.002852492 1.928120003 -1.189678607<br>Zn 0.001033852 1.312650693 1.782803529<br>Zn 1.966191973 -1.627258496 -0.169623940<br>Zn -1.969107515 -1.622410556 -0.171193513<br>H 1.709477230 2.160633620 -1.142983853<br>H -1.702437416 2.167521882 -1.141695720<br>SCF energy (Eh) = -7629,651768 (M=1)

Table S19. Cartesian coordinates and SCF energies of adducts from Figure S3.

Cluster	Adducts			
Pd_6	Pd -0.860371189 -0.986309731 1.207721642<br>Pd 1.376242493 0.849541710 -1.266451410<br>Pd 1.754975623 -1.026161507 0.725813160<br>Pd 0.448114761 1.447512708 1.205179469<br>Pd -1.422766232 0.856967596 -0.827211432<br>Pd -0.024831092 -1.504689997 -1.266626728<br>H 0.754902415 -0.117528504 1.791869318<br>H 0.025017594 0.109999229 -1.946068772<br>O -2.989429815 -0.774220950 0.844588400<br>C -3.040121195 0.127009803 0.018212499<br>O -3.800379765 0.851183350 -0.574256428<br>SCF energy (Eh) =-957.1794462 (M=3)	Pd 0.9977321 -0.7197530 1.5752536<br>Pd -0.5093204 0.7717344 -1.3898616<br>Pd 2.0103763 0.8878722 -0.4584090<br>Pd -0.3042290 1.5299307 1.0480460<br>Pd -1.5888136 -1.0444592 0.2985751<br>Pd 0.9426418 -1.5820412 -0.9478982<br>H 1.4439856 0.9286424 1.1645127<br>H -0.6393951 -1.1892464 -1.2011850<br>O -2.8722702 1.0686590 -1.0694766<br>C -3.0571778 0.2114997 -0.2376809<br>O -3.8386474 -0.2935911 0.5295116<br>SCF energy (Eh) = -957.1711993 (M=3)	Pd 1.3659034 1.3258016 0.0076112<br>Pd -1.9797304 -1.1783414 -0.0293450<br>Pd -1.0451851 1.0783630 -1.3493021<br>Pd -1.0414303 1.0825783 1.3487649<br>Pd 0.4326501 -1.0293982 1.3340676<br>Pd 0.4958954 -1.0325263 -1.3059537<br>H -0.2200969 1.9306771 -0.0284715<br>H -0.4825341 -1.9418850 -0.1544316<br>O 3.6877511 0.6414408 -0.0819036<br>C 3.7160651 -0.5197815 -0.0031334<br>O 3.8014365 -1.6674465 0.0735208<br>SCF energy (Eh) = -957.1709938 (M=3)	Pd -0.9556242 -1.0366711 0.8252152<br>Pd 1.6161955 1.1047275 -0.8013886<br>Pd -0.7163866 1.7302549 0.5947684<br>Pd -0.6056481 0.0485389 -1.6184213<br>Pd 1.3779429 -1.6574448 -0.5825717<br>Pd 1.2670240 0.0165880 1.6258021<br>H -1.5278319 0.4420042 -0.1628061<br>H 2.2046519 -0.3593042 0.1598418<br>O -3.8922260 -1.2401187 0.6903308<br>C -4.1712209 -0.4290347 -0.0903265<br>O -4.4691058 0.3670953 -0.8717889<br>SCF energy (Eh) = -957.1671815 (M=3)

Table S20. Cartesian coordinates and SCF energies of adducts from Figure S4.

Cluster	Adducts		
Pd_3Zn_3	Pd 0.516520947 -1.885684664 -0.055680015<br>Pd -1.248815887 0.179825472 0.651279977<br>Pd 1.821265859 0.556858366 -0.493592062<br>Zn -0.297490803 -0.191918691 -1.669583920<br>Zn 0.112066941 2.111772281 0.183855874<br>Zn 0.989274701 -0.265948750 1.764886396<br>H 1.713106557 2.300314420 -0.410118165<br>H -1.494637346 1.897635929 0.785538809<br>O -4.018321390 -0.839864310 0.606457807<br>C -4.353987359 -0.078612024 -0.200658012<br>O -4.706086079 0.671296169 -1.006000814<br>SCF energy (Eh) = -5910.983176 (M=1)	Pd 1.621245733 0.952242416 -0.677902702<br>Pd -0.723896210 1.194441560 0.836166859<br>Pd 0.354245814 -1.537978550 -0.531989703<br>Zn 1.262172511 -0.246906258 1.458526259<br>Zn -1.599837580 -1.049779578 0.793671856<br>Zn -0.641398512 0.535502750 -1.609545864<br>H -0.973875993 -2.486253959 0.080804974<br>H -2.093250371 0.427869857 1.585499690<br>O -4.154768953 -0.747252479 -0.306118098<br>C -4.341488121 0.392624661 -0.428974325<br>O -4.539400874 1.520574631 -0.558561686<br>SCF energy (Eh) = -5910.984251 (M=1)	Pd 1.255076231 -1.318997612 0.063541704<br>Pd 0.425099652 1.374178207 0.004692634<br>Pd -1.533297859 -1.238304146 0.030696922<br>Zn -0.092655686 -0.267932081 1.866202698<br>Zn -1.969261293 1.130716727 -0.038976796<br>Zn -0.044586360 -0.336970041 -1.809695540<br>H -2.983220537 -0.262072306 -0.082435832<br>H -0.909063605 2.500717684 -0.025106054<br>O 3.628114626 1.701606902 -0.045825102<br>C 3.762274109 0.553884212 -0.040974997<br>O 3.930492114 -0.594054848 -0.036351086<br>SCF energy (Eh) =-5910.985798 (M=1)

Table S21. Cartesian coordinates and SCF energies of CO_2 adducts from Figure S5.

Cluster	Physisorbed CO_2 adducts	Activated CO_2 adducts
Pd_6	Pd 1.605469 0.957005 -0.004872<br>Pd -1.990211 -0.954777 0.007589<br>Pd -0.290103 0.210478 1.834846<br>Pd 0.484069 -1.593017 0.109427<br>Pd -0.192442 -0.001217 -1.842221<br>Pd -1.161108 1.655183 -0.112392<br>O 3.928083 0.346796 -0.018907<br>C 2.992237 -0.417630 0.016113<br>O 2.707611 -1.607093 0.050662<br>SCF energy (Eh) = -955.965878 (M=3)	Pd 0.502124 -1.586618 0.015682<br>Pd -1.172106 1.654900 -0.011418<br>Pd 1.601507 0.969053 -0.026494<br>Pd -0.229781 0.127821 1.840348<br>Pd -1.982573 -0.966389 0.019985<br>Pd -0.256508 0.077171 -1.841941<br>O 2.671172 -1.601052 0.015483<br>C 2.978381 -0.413631 0.004028<br>O 3.934733 0.324627 0.003567<br>SCF energy (Eh) = -955.982275 (M=3)
Pd_2Zn_2	Pd -0.707699 0.294850 -0.419852<br>Pd 1.862752 -0.416573 -0.017046<br>Zn 0.015442 -2.022992 -0.045573<br>Zn 0.957049 1.764833 0.637821<br>O -4.501543 0.059070 0.837688<br>C -3.745019 0.421204 0.047018<br>O -2.999292 0.795544 -0.763362<br>SCF energy (Eh) = -4002.648243 (M=1)	Pd -1.104717 -1.300348 0.111987<br>Pd -1.082477 1.312142 0.115343<br>Zn 0.358973 -0.003070 -1.375730<br>Zn 0.839345 -0.010382 1.169550<br>O 2.311590 -0.002227 -1.153574<br>C 2.669777 -0.007451 0.083131<br>O 3.768755 -0.009553 0.557251<br>SCF energy (Eh) = -4002.609014 (M=1)
Pd_3Zn_3	Pd -1.110342 -1.610342 -0.049977<br>Pd -0.316362 1.089979 0.184722<br>Pd 1.572313 -0.709731 -0.229787<br>Zn -0.232447 -0.273853 -1.945119<br>Zn 2.091808 1.720518 -0.025897<br>Zn 0.178877 -0.730461 1.874065<br>O -2.319410 2.430131 0.420912<br>C -3.217926 1.726506 0.189875<br>O -4.132194 1.062098 -0.033120<br>SCF energy (Eh) = -5909.796476 (M=1)	Pd 1.709163 0.702158 -0.028019<br>Pd -1.206312 1.221001 -0.049883<br>Pd -0.243857 -1.238875 0.051702<br>Zn 0.098102 0.439349 1.932396<br>Zn -2.688912 -0.826946 0.034747<br>Zn 0.095912 0.277888 -1.962048<br>O 3.742678 -0.527727 0.031797<br>C 2.698850 -1.120416 0.041274<br>O 2.099836 -2.155182 0.068798<br>SCF energy (Eh) = -5909.792314 (M=1)

Table S22. Cartesian coordinates and SCF energies of structures in formate reaction paths (Figure 4).

Cluster	Adducts		
Pd_6	Pd 1.397367 0.531633 -0.865423<br>Pd -1.790994 -0.565299 0.720499<br>Pd 0.588135 0.231870 1.630342<br>Pd 0.507172 -1.993196 -0.025389<br>Pd -0.963086 -0.334657 -1.698177<br>Pd -0.870701 1.938918 -0.025840<br>O 3.828307 1.255143 -0.375398<br>C 4.318857 0.315918 0.102659<br>O 4.826120 -0.603448 0.579253<br>H 0.785970 -1.047597 -1.401228<br>H -0.960102 1.041391 1.394016<br>SCF energy (Eh) = -957.167010 (M=3)	Pd 1.097009 -1.196911 -0.257418<br>Pd -1.672848 1.205976 0.134762<br>Pd -1.113447 -0.838372 -1.570866<br>Pd 0.803863 1.271860 -1.165772<br>Pd 0.543720 0.894120 1.397974<br>Pd -1.212621 -1.314652 1.200106<br>O 3.477082 -1.348686 0.169990<br>C 4.075627 -0.357832 0.294629<br>O 4.697196 0.600848 0.430764<br>H 2.006699 1.882810 -0.394340<br>H -2.008532 -0.550161 -0.102273<br>SCF energy (Eh) = -957.161424 (M=3)	Pd 1.270061 1.111392 -0.249625<br>Pd -1.931345 -1.039224 0.381995<br>Pd -0.868118 1.283413 1.362783<br>Pd 0.767485 -0.960210 1.172739<br>Pd 0.205507 -1.317357 -1.267279<br>Pd -1.184511 0.988767 -1.360860<br>O 3.643474 0.934033 -0.582531<br>C 4.282918 0.067407 -0.140817<br>O 4.949099 -0.770759 0.280162<br>H 2.122675 -1.698434 0.957702<br>H -1.995327 0.683765 0.159095<br>SCF energy (Eh) = -957.161662(M=3)
	Pd 1.098895 -1.164057 0.651490<br>Pd -1.742658 1.165134 -0.692606<br>Pd -0.498559 -1.130156 -1.610803<br>Pd 1.016011 1.030283 -0.772911<br>Pd -0.187678 1.162762 1.497503<br>Pd -1.530608 -1.085583 0.961210<br>O 3.263279 -0.716172 0.664476<br>C 3.660255 0.155407 -0.064488<br>O 4.517061 0.629384 -0.707363<br>H 2.477990 1.323008 -0.128278<br>H -1.830770 -0.566744 -0.700305<br>SCF energy (Eh) = -957.149339(M=3)	Pd 1.187239 -1.165732 0.514935<br>Pd -1.750466 1.185075 -0.569573<br>Pd -0.558583 -1.100243 -1.636399<br>Pd 0.904794 1.110933 -0.860025<br>Pd -0.084980 1.105588 1.518836<br>Pd -1.443769 -1.142713 1.007637<br>O 3.208673 -0.837221 0.338817<br>C 3.619615 0.259862 -0.145400<br>O 4.722857 0.682672 -0.340592<br>H 2.734871 1.009971 -0.449255<br>H -1.787496 -0.562190 -0.583242<br>SCF energy (Eh) = -957.156248 (M=3)	Pd 1.319432 1.142183 -1.044447<br>Pd -1.448048 -1.162132 0.404706<br>Pd 0.402041 0.767283 1.455215<br>Pd 1.167126 -1.416312 0.034231<br>Pd -0.340351 -0.837003 -1.986982<br>Pd -1.301546 1.384899 -0.743451<br>O 3.340416 0.740593 -0.638705<br>C 3.251856 -0.196468 0.217273<br>O 3.363960 -0.178825 1.431091<br>H 3.099276 -1.258904 -0.280920<br>H -1.102303 0.513636 0.752189<br>SCF energy (Eh) = -957.151513 (M=3)
	Pd 1.759518 0.423079 -1.240465<br>Pd 0.668339 1.651901 0.984064<br>Pd 1.364699 -0.914125 1.144190<br>Pd -1.273665 -0.393734 1.217060<br>Pd -0.062951 -1.665245 -0.850765<br>Pd -0.894121 0.840177 -1.083716<br>C -3.670351 0.297398 -0.320407<br>O -2.958217 0.659539 -1.287224<br>H -4.758249 0.338493 -0.496383<br>O -3.321095 -0.102594 0.812828<br>H 2.033220 1.320873 0.137188<br>SCF energy (Eh) = -957.187124 (M=3)		
Pd_3Zn_3_I	Pd 2.393730 0.605370 -0.113590<br>Pd -0.079540 1.658780 0.114170<br>Pd 0.080140 -2.088460 -0.060220<br>Zn 0.737040 -0.290540 1.574440<br>Zn -1.520230 -0.304060 0.188750<br>Zn 0.484000 -0.149450 -1.619230<br>H -1.778640 1.497700 0.222540<br>H 1.613400 2.059860 0.107120<br>O -5.243500 1.419980 -0.271680<br>C -4.590130 0.493400 -0.086950<br>O -3.938660 -0.454830 0.098810<br>SCF energy (Eh) = -5910.974200 (M=1)	Pd 2.039010 1.063590 -0.010110<br>Pd -0.626530 1.466120 0.012350<br>Pd 0.741980 -1.981680 -0.011220<br>Zn 0.536780 -0.092740 1.652710<br>Zn -1.422940 -0.928300 0.027630<br>Zn 0.506480 -0.085540 -1.656120<br>H -2.263270 1.031540 0.021390<br>H 0.924680 2.289470 0.007840<br>O -4.498880 1.204440 -0.029950<br>C -3.741710 0.323120 -0.011800<br>O -3.491910 -0.863390 -0.004090<br>SCF energy (Eh) = -5910.957616 (M=1)	Pd 1.857440 1.197420 0.059310<br>Pd -0.787180 1.410270 -0.049200<br>Pd 1.012830 -1.827380 -0.008130<br>Zn 0.421300 -0.050050 1.692540<br>Zn -1.306270 -1.273760 -0.002270<br>Zn 0.515770 -0.026770 -1.702870<br>H -2.606520 1.047840 -0.186430<br>H 0.654700 2.355180 0.108990<br>O -4.559220 0.834780 0.091920<br>C -3.496330 0.318070 -0.052500<br>O -3.167850 -0.920760 0.011980<br>SCF energy (Eh) = -5910.969240 (M=1)
	Pd 1.576270 1.469390 0.043410<br>Pd -1.073820 1.258460 -0.118870<br>Pd 1.293870 -1.678880 0.084230<br>Zn 0.325140 0.030430 1.685580<br>Zn -1.075570 -1.561510 -0.073540<br>Zn 0.509390 -0.043430 -1.663800<br>H -2.905590 0.458740 -1.230990<br>H 0.213290 2.398810 -0.173910<br>O -3.760110 0.738080 0.579190<br>C -3.206320 0.025340 -0.226070<br>O -2.923610 -1.241430 -0.090320<br>SCF energy (Eh) = -5910.962449 (M=1)	Pd 1.772790 1.229930 -0.010600<br>Pd -0.904550 1.385330 0.004930<br>Pd 1.050970 -1.828180 0.009010<br>Zn 0.422550 -0.031270 1.681350<br>Zn -1.266460 -1.243700 -0.010100<br>Zn 0.416230 -0.042860 -1.676070<br>H -4.734200 0.171340 -0.002250<br>H 0.529060 2.335140 -0.049430<br>O -2.988280 1.139740 0.013480<br>C -3.638380 0.083560 0.001480<br>O -3.188900 -1.099560 -0.009260<br>SCF energy (Eh) = -5911.009640 (M=1)	
Pd_4Zn_4	Pd 1.736272 0.701500 0.511162<br>Pd -1.166007 1.274350 0.861943<br>Pd 0.431091 -2.034269 0.254970<br>Zn 0.293820 -0.360111 2.202269<br>Zn -1.781935 -0.967671 0.093243<br>Pd -0.011516 0.124536 -1.553708<br>H -1.181292 -2.559483 -0.115607<br>H -2.661343 0.424901 0.623496<br>O -4.506864 -1.288661 -0.897964<br>C -5.079941 -0.298816 -0.700731<br>O -5.660187 0.679586 -0.510210<br>Zn 2.220390 -1.028201 -1.226236<br>Zn 0.422575 2.447997 -0.707770<br>SCF energy (Eh) = -7818.123100 (M=1)	Pd 1.590557 0.795414 0.804330<br>Pd -1.283391 1.066727 0.660675<br>Pd 0.546362 -1.889127 0.237048<br>Zn 0.020010 -0.409921 2.276532<br>Zn -1.678604 -1.104668 -0.442807<br>Pd 0.322015 0.189394 -1.623579<br>H -0.790640 -2.628722 -0.505803<br>H -2.799883 0.443317 0.175325<br>O -3.731970 -1.323057 -0.919443<br>C -4.177887 -0.296214 -0.463430<br>O -5.049670 0.439292 -0.235810<br>Zn 2.556712 -0.788081 -0.872372<br>Zn 0.390983 2.472067 -0.623139<br>SCF energy (Eh) = -7818.105800 (M=1)	Pd 1.643053 1.295531 -0.507772<br>Pd -1.285464 1.457325 -0.256015<br>Pd 0.432188 -0.397710 1.163126<br>Zn 0.359327 2.055142 1.552320<br>Zn -1.760736 -1.148637 0.515438<br>Pd 0.391841 -2.365987 -0.548128<br>H -0.473623 -1.614329 1.836805<br>H -3.250186 0.898486 -0.256509<br>O -3.629077 -1.032921 0.331716<br>C -4.080216 0.116583 -0.054863<br>O -5.219485 0.441811 -0.219510<br>Zn 2.593437 -1.023542 0.036487<br>Zn -0.029069 -0.181016 -1.615161<br>SCF energy (Eh) = -7818.134500
	Pd 2.076504 1.299122 -0.219238<br>Pd -0.787637 1.834906 0.457712<br>Pd 0.616474 -0.508044 1.111283<br>Zn 1.128692 1.703036 2.108482<br>Zn -1.718879 -0.744770 0.560277<br>Pd 0.206364 -1.940130 -1.055540<br>H -0.442576 -1.655036 1.643356<br>H -4.651081 0.671208 0.109222<br>O -3.525403 -0.992125 0.389561<br>C -4.432247 -0.340113 -0.295193<br>O -5.024788 -0.762960 -1.250862<br>Zn 2.562363 -1.198789 -0.301271<br>Zn 0.052736 0.490408 -1.432103<br>SCF energy (Eh) = -7818.130300 (M=1)	Pd -0.937039 0.617254 1.304115<br>Pd 1.823255 0.528504 0.854148<br>Pd -0.975314 0.705366 -1.562925<br>Zn 0.279057 2.302421 -0.029678<br>Zn 1.140092 -0.523272 -1.321998<br>Pd -0.990395 -1.778728 -0.352511<br>H -0.082359 -0.230860 -2.627712<br>O 2.978907 -1.046278 -1.721516<br>C 3.902135 -0.620805 -0.967013<br>O 3.789888 0.071546 0.054828<br>Zn -2.915833 -0.060951 -0.060264<br>Zn 0.482227 -1.421204 1.585735<br>H 4.920637 -0.908513 -1.272006<br>SCF energy (Eh) = -7818.158700 (M=1)	

Table S23. Cartesian coordinates and SCF energies of structures in Figure S6.

Cluster	Adducts		
Pd_3Zn_3_II	Pd 1.621246 0.952242 -0.677903<br>Pd -0.723896 1.194442 0.836167<br>Pd 0.354246 -1.537979 -0.531990<br>Zn 1.262173 -0.246906 1.458526<br>Zn -1.599838 -1.049780 0.793672<br>Zn -0.641399 0.535503 -1.609546<br>H -0.973876 -2.486254 0.080805<br>H -2.093250 0.427870 1.585500<br>O -4.154769 -0.747252 -0.306118<br>C -4.341488 0.392625 -0.428974<br>O -4.539401 1.520575 -0.558562<br>SCF energy (Eh) = -5910.984251 (M=1)	Pd 2.148332 0.691200 0.065924<br>Pd -0.570384 1.232324 -0.054199<br>Pd 0.600184 -1.594720 0.018600<br>Zn 0.568355 0.113495 1.898542<br>Zn -1.691297 -0.937869 -0.075666<br>Zn 0.736910 0.080365 -1.886051<br>H -0.843727 -2.501624 -0.117787<br>H -2.249095 1.106268 -0.116560<br>O -3.747072 -0.664765 0.007492<br>C -3.890319 0.534885 0.026102<br>O -4.519941 1.505520 0.083220<br>SCF energy (Eh) = -5910.968083 (M=1)	Pd 2.135307 0.649150 0.003851<br>Pd -0.539066 1.165324 -0.078089<br>Pd 0.529887 -1.534734 0.067858<br>Zn 0.610974 0.208439 1.936137<br>Zn -1.808829 -1.165153 0.044093<br>Zn 0.690338 0.025710 -1.942534<br>H -0.791429 -2.606967 0.082712<br>H -2.467226 1.445002 -0.156089<br>O -3.491111 -0.344037 0.023097<br>C -3.517030 0.945897 -0.056735<br>O -4.465915 1.668031 -0.047783<br>SCF energy (Eh) = -5910.989450 (M=1)
	Pd -2.046867 0.527096 -0.361182<br>Pd 0.610888 0.574521 -1.022780<br>Pd -0.393120 -0.883530 1.275788<br>Zn -0.812092 -1.483813 -1.172041<br>Zn 1.921838 -0.863629 0.739716<br>Zn -0.315572 1.658670 1.040710<br>H 0.982404 -1.614823 1.992557<br>H 4.594019 0.652562 0.840683<br>O 3.324145 -0.397048 -0.361472<br>C 4.271045 0.501521 -0.210873<br>O 4.778977 1.111663 -1.109459<br>SCF energy (Eh) = -5910.982249 (M=1)	Pd 2.056500 0.648992 -0.006707<br>Pd -0.600488 1.220326 -0.002093<br>Pd 0.530058 -1.608525 0.000273<br>Zn 0.559475 0.075123 1.900978<br>Zn -1.766035 -1.041302 0.011305<br>Zn 0.550093 0.067341 -1.905648<br>H -0.859984 -2.584147 0.026422<br>H -4.616789 1.387374 -0.015938<br>O -3.531782 -0.291568 0.001039<br>C -3.596091 0.977874 -0.008214<br>O -2.658680 1.783941 -0.009542<br>SCF energy (Eh) = -5911.023018 (M=1)	
Pd_4Zn_4	Pd -0.833364 -2.252022 -0.344198<br>Pd -2.390367 -0.064177 0.452878<br>Pd 1.626754 0.734459 1.263336<br>Pd 1.109762 1.294415 -1.396268<br>Zn -0.307394 -0.711851 1.628993<br>Zn -0.948612 -0.014301 -1.565641<br>Zn 1.373681 -1.030671 -0.510137<br>Zn -0.649199 1.766678 0.489998<br>H -2.423659 -1.753350 0.093442<br>H 0.774747 -2.691061 -0.770210<br>O 3.989139 -1.683286 -0.442680<br>C 4.522058 -0.803114 0.097400<br>O 5.071794 0.059244 0.630013<br>SCF energy (Eh) = -7818.140200 (M=1)	Pd -0.385286 -0.891101 -1.392172<br>Pd -0.745013 -2.293127 0.839889<br>Pd 0.842105 2.291170 1.049115<br>Pd -1.504968 1.602065 -0.236743<br>Zn 1.569014 -1.753590 -0.119565<br>Zn -2.602419 -0.645898 -0.111253<br>Zn 0.928536 1.142909 -1.117521<br>Zn -0.126936 0.049921 1.158142<br>H 3.140791 -1.336422 -0.322584<br>H 0.253045 0.159886 -2.528347<br>O 3.092732 0.770207 -1.374080<br>C 3.939505 0.095523 -0.874133<br>O 5.032888 -0.150569 -0.553235<br>SCF energy (Eh) = -7818.099300 (M=1)	Pd 0.708035 -0.939193 0.819796<br>Pd 2.175024 0.712915 -0.676567<br>Pd -2.272448 -0.302547 -1.276544<br>Pd -0.595307 1.430068 0.025270<br>Zn 2.874135 -1.639494 -0.144234<br>Zn 1.293959 1.382287 1.617466<br>Zn -1.689131 -0.852725 1.091726<br>Zn 0.133036 -0.381557 -1.627762<br>H -0.329905 -1.876328 1.771614<br>O -4.214688 -0.265512 -0.353785<br>C -4.395766 -0.491158 0.850249<br>O -3.524212 -0.772042 1.726254<br>H -5.431674 -0.448047 1.220890<br>SCF energy (Eh) = -7818.162700 (M=1)

Table S24. Cartesian coordinates and SCF energies of structures in Figure 5.

Cluster	Adducts		
Pd_3Zn_3_I	Pd 2.078100 1.235940 -0.020350<br>Pd -0.557030 0.221890 0.006570<br>Pd 1.481120 -1.491660 -0.004230<br>Zn 1.108060 0.071950 1.938990<br>Zn -0.860250 -2.273190 0.014700<br>Zn 1.074640 0.057440 -1.951970<br>H -2.197440 0.757690 0.013810<br>H -1.751660 1.474960 0.010430<br>O -4.823080 2.820090 0.112530<br>C -5.018100 1.682180 0.040620<br>O -5.211160 0.543860 -0.031860<br>SCF energy (Eh) = -5910.985542 (M=1)	Pd 2.054360 1.112560 -0.095200<br>Pd -0.621980 0.798030 0.039270<br>Pd 0.779130 -1.539460 0.046020<br>Zn 1.003080 0.081530 1.927300<br>Zn -1.724830 -1.445240 0.041840<br>Zn 0.838620 -0.041470 -1.939290<br>H -1.766930 1.860830 0.035820<br>H 0.181240 2.137530 -0.133250<br>O -4.773200 2.108130 0.088960<br>C -4.703340 0.960960 -0.015720<br>O -4.655530 -0.193170 -0.120000<br>SCF energy (Eh) = -5910.961695 (M=1)	Pd 1.949800 1.286180 -0.019730<br>Pd -0.729220 0.672700 0.068630<br>Pd 0.964280 -1.508130 -0.058210<br>Zn 0.919390 0.006890 1.911080<br>Zn -1.549700 -1.655120 0.023590<br>Zn 0.765930 0.112920 -1.928040<br>H -1.857660 1.764650 0.128900<br>H 0.345970 1.936210 0.042280<br>O -4.782480 2.130110 0.108520<br>C -4.673700 0.985190 0.002860<br>O -4.594830 -0.166030 -0.103360<br>SCF energy (Eh) = -5910.963874 (M=1)
	Pd 2.142740 0.955100 -0.080790<br>Pd -0.550100 1.154140 0.136540<br>Pd 0.797240 -1.682000 -0.083860<br>Zn 0.818140 -0.100620 1.844630<br>Zn -1.563860 -1.029360 0.051990<br>Zn 0.541870 0.049670 -1.843760<br>H -1.985280 1.821650 0.219170<br>H 0.892590 2.096590 -0.079690<br>O -5.066950 1.486290 -0.038640<br>C -4.662720 0.408880 -0.018910<br>O -4.276800 -0.688270 -0.001190<br>SCF energy (Eh) = -5910.957615 (M=1)	Pd 2.393730 0.605370 -0.113590<br>Pd -0.079540 1.658780 0.114170<br>Pd 0.080140 -2.088460 -0.060220<br>Zn 0.737040 -0.290540 1.574440<br>Zn -1.520230 -0.304060 0.188750<br>Zn 0.484000 -0.149450 -1.619230<br>H -1.778640 1.497700 0.222540<br>H 1.613400 2.059860 0.107120<br>O -5.243500 1.419980 -0.271680<br>C -4.590130 0.493400 -0.086950<br>O -3.938660 -0.454830 0.098810<br>SCF energy (Eh) = -5910.974200 (M=1)	Pd 2.039010 1.063590 -0.010110<br>Pd -0.626530 1.466120 0.012350<br>Pd 0.741980 -1.981680 -0.011220<br>Zn 0.536780 -0.092740 1.652710<br>Zn -1.422940 -0.928300 0.027630<br>Zn 0.506480 -0.085540 -1.656120<br>H -2.263270 1.031540 0.021390<br>H 0.924680 2.289470 0.007840<br>O -4.498880 1.204440 -0.029950<br>C -3.741710 0.323120 -0.011800<br>O -3.491910 -0.863390 -0.004090<br>SCF energy (Eh) = -5910.957616 (M=1)
	Pd 1.857440 1.197420 0.059310<br>Pd -0.787180 1.410270 -0.049200<br>Pd 1.012830 -1.827380 -0.008130<br>Zn 0.421300 -0.050050 1.692540<br>Zn -1.306270 -1.273760 -0.002270<br>Zn 0.515770 -0.026770 -1.702870<br>H -2.606520 1.047840 -0.337920<br>H 0.654700 2.355180 0.108990<br>O -4.559220 0.834780 0.091920<br>C -3.496330 0.318070 -0.052500<br>O -3.167850 -0.920760 0.011980<br>SCF energy (Eh) = -5910.969240 (M=1)	Pd 1.576270 1.469390 0.043410<br>Pd -1.073820 1.258460 -0.118870<br>Pd 1.293870 -1.678880 0.084230<br>Zn 0.325140 0.030430 1.685580<br>Zn -1.075570 -1.561510 -0.073540<br>Zn 0.509390 -0.043430 -1.663800<br>H -2.905590 0.458740 -1.230990<br>H 0.213290 2.398810 -0.173910<br>O -3.760110 0.738080 0.579190<br>C -3.206320 0.025340 -0.226070<br>O -2.923610 -1.241430 -0.090320<br>SCF energy (Eh) = -5910.962449 (M=1)	Pd 1.772790 1.229930 -0.010600<br>Pd -0.904550 1.385330 0.004930<br>Pd 1.050970 -1.828180 0.009010<br>Zn 0.422550 -0.031270 1.681350<br>Zn -1.266460 -1.243700 -0.010100<br>Zn 0.416230 -0.042860 -1.676070<br>H -4.734200 0.171340 -0.002250<br>H 0.529060 2.335140 -0.049430<br>O -2.988280 1.139740 0.013480<br>C -3.638380 0.083560 0.001480<br>O -3.188900 -1.099560 -0.009260<br>SCF energy (Eh) = -5911.009640 (M=1)
Pd_3Zn_3_II	Pd 1.131734319 -1.580367127 0.183934532<br>Pd -0.100307081 0.983653460 -0.158346994<br>Pd -1.679030323 -1.032210320 0.352872085<br>Zn 0.043293161 -0.269652215 2.027366186<br>Zn -2.595475425 1.297921234 0.005871245<br>Zn -0.316647324 -0.956256075 -1.767785332<br>O 1.799757516 2.433305554 -0.513818516<br>C 2.788339860 1.844762629 -0.335744051<br>O 3.786471877 1.296677676 -0.164984321<br>H 2.557320935 -2.546679825 -0.199985719<br>H 2.650190884 -2.382252490 0.602077685<br>SCF energy (Eh) = -5910.983931 (M=1)	Pd -1.671461 0.009071 0.290649<br>Pd 0.788793 0.000340 -1.347313<br>Pd 0.958314 -0.008950 1.318218<br>Zn 0.010065 -1.953771 -0.029087<br>Zn 3.031318 -0.005160 -0.128832<br>Zn 0.037877 1.956125 -0.017320<br>O -5.134216 -0.012491 0.198299<br>C -4.054579 0.003129 -0.254578<br>O -3.306166 0.021128 -1.204063<br>H -0.923784 -0.018001 1.668723<br>H -3.083241 -0.006918 1.130528<br>SCF energy (Eh) = -5910.943041 (M=1)	Pd -1.431059 -0.330310 1.601783<br>Pd -0.589075 0.395647 -0.952742<br>Pd 1.288309 0.011687 0.983716<br>Zn -0.155127 -1.858945 0.052016<br>Zn 1.811705 0.605595 -1.402156<br>Zn -0.384189 1.907857 1.036853<br>O -4.564371 0.403387 -1.021978<br>C -3.524039 0.156064 -0.476448<br>O -3.297560 -0.177051 0.731760<br>H 0.066673 -0.442878 2.242394<br>H -2.565343 0.216449 -1.158973<br>SCF energy (Eh) = -5910.964527 (M=1)
	Pd -1.486808 -0.422822 1.642528<br>Pd -0.767477 0.179913 -0.983068<br>Pd 1.170961 0.141413 0.943949<br>Zn -0.080255 -1.926473 0.175523<br>Zn 1.585160 0.683281 -1.469457<br>Zn -0.692666 1.862185 0.907647<br>O -3.657427 1.330447 -0.779839<br>C -3.291255 0.225531 -0.434370<br>O -3.345051 -0.327447 0.719528<br>H 0.025842 -0.396492 2.243775<br>H -2.849539 -0.464914 -1.254809<br>SCF energy (Eh) = -5910.962292 (M=1)	Pd 1.572739 -1.002262 0.018625<br>Pd -0.064299 1.242240 -0.015766<br>Pd -1.262092 -1.207651 0.019901<br>Zn 0.091634 -0.289559 1.938480<br>Zn -2.485776 0.980453 -0.015975<br>Zn 0.094741 -0.348487 -1.923005<br>O 1.770318 2.385021 -0.032825<br>C 2.845199 1.749618 -0.023613<br>O 3.044142 0.510533 -0.005240<br>H 0.368381 -2.084618 0.031228<br>H 3.763351 2.362296 -0.032976<br>SCF energy (Eh) = -5911.000079 (M=1)	
Pd_6	Pd 0.907547 -1.577815 -0.007225<br>Pd -1.525979 1.577724 0.011513<br>Pd 1.266470 1.157328 0.004518<br>Pd -0.327733 -0.078876 1.806075<br>Pd -1.722097 -1.235652 0.000171<br>Pd -0.333225 -0.065349 -1.801122<br>O 2.994023 -1.067272 -0.025462<br>C 2.955692 0.162268 -0.011270<br>O 3.679925 1.129868 -0.002558<br>H -1.033450 3.190158 0.025450<br>H -1.891087 3.238832 0.034605<br>SCF energy (Eh) = -957.176002 (M=3)	Pd -1.456983 1.088186 0.102816<br>Pd 1.847835 -1.169512 -0.133496<br>Pd -0.834676 -1.577546 0.057982<br>Pd 0.487099 -0.022869 1.834254<br>Pd 1.111409 1.713493 0.017461<br>Pd 0.121490 0.248677 -1.828134<br>O -3.245566 -0.135477 0.017586<br>C -2.773857 -1.268261 -0.060159<br>O -3.097170 -2.427250 -0.166690<br>H 1.290135 -2.519339 0.376692<br>H 1.870515 -1.032069 1.494224<br>SCF energy (Eh) = -957.158942 (M=3)	Pd 0.845740 -1.444276 0.150055<br>Pd -1.298266 2.713442 -0.634713<br>Pd 1.056516 1.268788 -0.147831<br>Pd -1.058783 0.384576 1.155133<br>Pd -1.693677 -1.835390 0.007123<br>Pd -0.897361 -0.001175 -1.551954<br>O 2.916255 -0.886058 0.334694<br>C 2.800377 0.307967 0.108100<br>O 3.408871 1.340558 -0.031762<br>H 0.272898 2.843711 -0.164852<br>H -1.631831 1.918326 0.724560<br>SCF energy (Eh) = -957.165500 (M=3)
	Pd 0.994718 0.913818 -0.770363<br>Pd -1.683128 -1.315890 1.038982<br>Pd 1.039313 -1.360257 0.621350<br>Pd -0.701113 -1.137661 -1.547253<br>Pd -1.638674 1.175133 -0.313928<br>Pd -0.045244 0.746268 1.734688<br>O 3.060664 0.351381 -0.484275<br>C 3.059943 -0.833819 -0.117579<br>O 3.682357 -1.833709 -0.304616<br>H 2.399597 -0.930815 1.346791<br>H -1.909090 -0.554641 -0.477800<br>SCF energy (Eh) = -957.150204 (M=3)	Pd 1.759518 0.423079 -1.240465<br>Pd 0.668339 1.651901 0.984064<br>Pd 1.364699 -0.914125 1.144190<br>Pd -1.273665 -0.393734 1.217060<br>Pd -0.062951 -1.665245 -0.850765<br>Pd -0.894121 0.840177 -1.083716<br>C -3.670351 0.297398 -0.320407<br>O -2.958217 0.659539 -1.287224<br>H -4.758249 0.338493 -0.496383<br>O -3.321095 -0.102594 0.812828<br>H 2.033220 1.320873 0.137188<br>SCF energy (Eh) = -957.187124 (M=3)	

Table S25. Cartesian coordinates and SCF energies of structures in COOH group reaction paths (Figure 6 Panel A).

Cluster	Adducts		
Pd_6	Pd 0.845562 -1.238729 1.008033<br>Pd -1.339885 1.170321 -1.091779<br>Pd 1.432968 0.968847 -0.582611<br>Pd -0.455132 1.174765 1.489213<br>Pd -1.788008 -1.070608 0.509554<br>Pd -0.018782 -1.217173 -1.491516<br>O 2.972161 -1.054666 0.609950<br>C 3.044081 0.014607 0.017627<br>O 3.823453 0.827935 -0.411367<br>H -0.663475 -0.534441 1.728349<br>H -0.009197 0.490329 -1.862642<br>SCF energy (Eh) = -957.179787 (M=3)	Pd 0.799739 -1.588410 -0.037498<br>Pd -1.367819 1.409523 -0.088423<br>Pd 1.368786 1.038808 0.535446<br>Pd -0.896226 -0.401527 1.719332<br>Pd -1.908077 -1.203821 -0.759374<br>Pd 0.228090 0.296522 -1.774685<br>O 2.977302 -1.146378 -0.227588<br>C 2.967631 0.060309 -0.029310<br>O 3.867648 0.946489 -0.127646<br>H -0.858635 -1.700155 0.472923<br>H 2.796190 1.828486 0.158695<br>SCF energy (Eh) = -957.117412 (M=3)	Pd 0.650895 -1.700193 -0.000140<br>Pd -1.190890 1.682673 0.000203<br>Pd 1.534933 1.095364 -0.000315<br>Pd -0.260740 0.076923 1.765657<br>Pd -2.085118 -0.974128 0.000327<br>Pd -0.261433 0.076937 -1.765622<br>O 2.798720 -1.390511 -0.000461<br>C 3.016601 -0.170216 -0.000396<br>O 4.288821 0.231018 -0.000332<br>H -0.932366 -2.185116 0.000169<br>H 4.844873 -0.568435 -0.000327<br>SCF energy (Eh) = -957.168920 (M=3)
Pd_3Zn_3	Pd 0.516521 -1.885685 -0.055680<br>Pd -1.248816 0.179825 0.651280<br>Pd 1.821266 0.556858 -0.493592<br>Zn -0.297491 -0.191919 -1.669584<br>Zn 0.112067 2.111772 0.183856<br>Zn 0.989275 -0.265949 1.764886<br>H 1.713107 2.300314 -0.410118<br>H -1.494637 1.897636 0.785539<br>O -4.018321 -0.839864 0.606458<br>C -4.353987 -0.078612 -0.200658<br>O -4.706086 0.671296 -1.006001<br>SCF energy (Eh) = -5910.983176 (M=1)	Pd 1.327006 -1.547514 -0.010565<br>Pd -1.232246 -0.434339 0.000833<br>Pd 1.463991 1.214503 -0.025424<br>Zn 0.547253 -0.152318 -1.923384<br>Zn -0.840810 1.966452 -0.017761<br>Zn 0.569248 -0.132673 1.897358<br>H 0.752399 2.776913 -0.029232<br>H -2.572450 0.909043 -0.004838<br>O -3.450408 -1.972052 0.067748<br>C -3.208780 -0.811829 0.037312<br>O -3.798108 0.301543 0.018828<br>SCF energy (Eh) = -5910.918150 (M=1)	Pd -1.671293 -1.235498 -0.639736<br>Pd -1.345057 1.413353 0.132728<br>Pd 1.070768 -0.895654 -0.132864<br>Zn -0.298636 0.368900 -1.933768<br>Zn 1.042071 1.415108 0.443136<br>Zn -0.826797 -0.584340 1.600144<br>H 3.342708 -0.148974 -1.369984<br>H -0.229893 2.622514 0.649455<br>O 3.870399 -0.377310 -0.595524<br>C 3.007527 -0.735404 0.387806<br>O 3.418382 -1.072462 1.454211<br>SCF energy (Eh) = -5910.965100(M=1)
	Pd -1.688300 -1.359597 -0.080258<br>Pd -1.373467 1.400089 0.047850<br>Pd 1.082281 -0.901809 -0.068040<br>Zn -0.619537 -0.072564 -1.897750<br>Zn 1.029658 1.478996 -0.122360<br>Zn -0.532184 -0.209303 1.795910<br>H 3.995549 -0.437995 -1.470052<br>H -0.238031 2.690835 0.104345<br>O 3.725643 -1.169356 -0.901880<br>C 3.040974 -0.662959 0.193125<br>O 3.609829 -0.340152 1.187781<br>SCF energy (Eh) = -5910.947894 (M=1)	Pd 2.076653 0.006542 0.031699<br>Pd 0.399832 2.206592 -0.016025<br>Pd -0.567808 -0.940822 0.055433<br>Zn 0.506997 0.500981 1.877529<br>Zn -1.749227 1.191403 0.095180<br>Zn 0.483132 0.424002 -1.820789<br>H -4.267421 -0.438910 -0.135587<br>H -1.204406 2.856042 -0.031098<br>O -3.320862 -0.203442 0.058037<br>C -2.549334 -1.354302 -0.104797<br>O -3.074218 -2.395711 -0.402249<br>SCF energy (Eh) = -5910.974000 (M=1)	Pd 1.933689 -0.924522 0.013586<br>Pd 0.811839 1.582992 -0.033799<br>Pd -0.821451 -1.115799 0.024275<br>Zn 0.548476 -0.056218 1.884797<br>Zn -1.544712 1.312500 -0.016670<br>Zn 0.538059 -0.132022 -1.884096<br>H -4.294007 -0.325236 0.007124<br>H -0.476020 2.722356 -0.043655<br>O -3.288842 0.381504 0.003038<br>C -2.814197 -0.907423 0.019519<br>O -3.859086 -1.590456 0.023602<br>SCF energy (Eh) = -5910.939918(M=1)
		Pd 0.527953136 1.791200295 0.116291461<br>Pd -0.123939089 -1.264066828 -0.059884847<br>Zn 0.846955492 0.022963954 1.901108478<br>Zn -1.625295459 0.781554905 0.044282612<br>Zn 0.852654667 0.231417943 -1.861594786<br>H -1.076857696 2.426258793 0.149099848<br>O -2.946500725 -0.652819533 -0.064823290<br>C -2.108766837 -1.618005223 -0.107012266<br>O -2.659917515 -2.812406719 -0.187001081<br>H -3.628890314 -2.694488129 -0.196787874<br>SCF energy (Eh) = -5910.990917 (M=1)	
Pd_4Zn_4	Pd -0.833364 -2.252022 -0.344198<br>Pd -2.390367 -0.064177 0.452878<br>Pd 1.626754 0.734459 1.263336<br>Pd 1.109762 1.294415 -1.396268<br>Zn -0.307394 -0.711851 1.628993<br>Zn -0.948612 -0.014301 -1.565641<br>Zn 1.373681 -1.030671 -0.510137<br>Zn -0.649199 1.766678 0.489998<br>H -2.423659 -1.753350 0.093442<br>H 0.774747 -2.691061 -0.770210<br>O 3.989139 -1.683286 -0.442680<br>C 4.522058 -0.803114 0.097400<br>O 5.071794 0.059244 0.630013<br>SCF energy (Eh) = -7818.140200 (M=1)	Pd 2.319874 0.285433 -0.017459<br>Pd 0.372794 2.360991 -0.023677<br>Pd -1.713185 -0.901734 -1.395541<br>Pd -1.696511 -0.876811 1.425915<br>Zn 0.315600 0.436157 -1.611773<br>Zn 0.335086 0.464862 1.598312<br>Zn 0.089692 -1.869630 0.013807<br>Zn -1.863944 1.251950 -0.002400<br>H 2.003773 1.988386 -0.029008<br>H 3.641961 -0.854452 -0.015704<br>O 3.987675 -2.175723 -0.011241<br>C 2.763098 -1.854635 -0.005100<br>O 1.832980 -2.705743 0.006744<br>SCF energy (Eh) = -7818.072400 (M=1)	Pd 2.268238 0.297363 0.001531<br>Pd 0.325641 2.392973 0.004924<br>Pd -1.683568 -0.926406 -1.403392<br>Pd -1.683349 -0.930555 1.404884<br>Zn 0.310714 0.467631 -1.585089<br>Zn 0.311049 0.463006 1.589251<br>Zn 0.152394 -1.855753 -0.000735<br>Zn -1.888657 1.222147 0.003810<br>H 1.972832 1.992535 0.004618<br>H 4.587419 -1.335115 -0.003066<br>O 4.020292 -2.113647 -0.003952<br>C 2.746891 -1.703406 -0.002301<br>O 1.946090 -2.664405 -0.003304<br>SCF energy (Eh) = -7818.144800 (M=1)

Table S26. Cartesian coordinates and SCF energies of structures in COOH group reaction paths (Figure 6 Panel B).

Cluster	Adducts		
Pd_6	Pd 0.907547 -1.577815 -0.007225<br>Pd -1.525979 1.577724 0.011513<br>Pd 1.266470 1.157328 0.004518<br>Pd -0.327733 -0.078876 1.806075<br>Pd -1.722097 -1.235652 0.000171<br>Pd -0.333225 -0.065349 -1.801122<br>O 2.994023 -1.067272 -0.025462<br>C 2.955692 0.162268 -0.011270<br>O 3.679925 1.129868 -0.002558<br>H -1.033450 3.190158 0.025450<br>H -1.891087 3.238832 0.034605<br>SCF energy (Eh) = -957.176002 (M=3)	Pd 0.889431 1.444684 0.242451<br>Pd -1.399502 -1.782744 -0.150142<br>Pd 1.146227 -1.219618 -0.142523<br>Pd -0.311313 -0.082372 -1.975810<br>Pd -1.647121 0.931549 0.124256<br>Pd -0.264657 -0.091706 2.007113<br>O 3.171998 0.986778 0.171965<br>C 3.013649 -0.185977 0.328901<br>O 3.389199 -1.279901 0.612238<br>H -1.197054 -1.485453 -1.764818<br>H 1.040859 -2.452704 -1.088321<br>SCF energy (Eh) = -957.142744 (M=3)	Pd 0.397037 -0.817918 0.154216<br>Pd -0.782910 1.684188 0.626980<br>Pd 1.721043 2.102693 -0.776834<br>Pd -1.453563 -0.428020 2.153919<br>Pd -2.242020 -0.772731 -0.409974<br>Pd -0.493559 0.807930 -1.709061<br>O 2.591430 -0.528662 0.026863<br>C 2.763058 0.670530 0.082470<br>O 3.462592 1.540653 0.555077<br>H -2.217149 0.383863 0.854837<br>H 0.763903 1.835671 -2.163475<br>SCF energy (Eh) = -957.180754 (M=3)
	Pd -0.386568 -1.047735 1.202328<br>Pd 0.949192 1.326009 -1.300602<br>Pd -1.540890 0.106314 -0.838334<br>Pd 0.849342 -1.345090 -1.318625<br>Pd 2.098979 0.066799 0.766105<br>Pd -0.259612 1.634599 0.926414<br>O -2.498413 -1.226629 1.502433<br>C -3.080867 -0.868812 0.464179<br>O -4.064142 -1.150484 -0.148430<br>H 1.175223 -1.302598 0.362768<br>H -2.697874 0.618606 0.154427<br>SCF energy (Eh) = -957.146057 (M=3)	Pd -0.838041428 -0.402453567 1.270964980<br>Pd 1.573618106 0.567733234 -1.337305258<br>Pd -1.019928350 -0.043446774 -1.330491105<br>Pd 0.890470985 -1.887458281 -0.422400801<br>Pd 1.846271947 0.120896498 1.283721720<br>Pd 0.003615413 1.919163116 0.176512353<br>O -2.854112422 -0.035776075 1.253474524<br>C -3.462951477 0.141292831 0.155669185<br>O -4.590698151 0.459978946 -0.089464292<br>H 0.787912129 -1.209988843 1.130695376<br>H -2.786031852 -0.067329684 -0.814279681<br>SCF energy (Eh) = -957.154255 (M=3)	Pd -0.322183 -1.374456 1.046336<br>Pd 0.791677 1.396983 -1.168673<br>Pd -1.692940 0.052883 -0.873972<br>Pd 0.928456 -1.176648 -1.341863<br>Pd 2.133916 0.051826 0.834611<br>Pd -0.370975 1.252557 1.126523<br>O -2.517082 -1.447196 1.365348<br>C -2.892933 -0.684706 0.485763<br>O -4.026949 -0.186701 0.222734<br>H 1.308982 -1.391446 0.523323<br>H -3.291400 0.528740 -0.759121<br>SCF energy (Eh) = -957.116990 (M=3)
	Pd 0.124434 -0.865840 0.122443<br>Pd -1.971678 2.228627 0.130527<br>Pd 0.804019 1.745083 0.165412<br>Pd -1.143462 0.632219 1.983326<br>Pd -2.700835 -0.430270 -0.058497<br>Pd -0.879027 0.804364 -1.708912<br>O 2.209388 -0.566668 -0.469082<br>C 2.336682 0.655270 -0.386618<br>O 3.542427 1.141731 -0.719066<br>H -1.411531 -0.951503 0.879417<br>H 3.505464 2.099174 -0.620000<br>SCF energy (Eh) = -957.167760 (M=3)	Pd 0.254802 -1.624487 -0.363713<br>Pd -0.948441 1.799779 0.518170<br>Pd 1.574398 0.524725 0.586593<br>Pd -0.871065 -0.441475 1.796444<br>Pd -2.301238 -0.355487 -0.579837<br>Pd 0.018747 0.720044 -1.602806<br>O 2.451417 -1.769643 -0.720107<br>C 2.835302 -0.748830 -0.159364<br>O 4.187031 -0.558604 -0.022852<br>H -1.342816 -1.477864 0.232005<br>H 4.526316 -0.094927 -0.797714<br>SCF energy (Eh) = -957.150222 (M=3)	Pd 0.650895 -1.700193 -0.000140<br>Pd -1.190890 1.682673 0.000203<br>Pd 1.534933 1.095364 -0.000315<br>Pd -0.260740 0.076923 1.765657<br>Pd -2.085118 -0.974128 0.000327<br>Pd -0.261433 0.076937 -1.765622<br>O 2.798720 -1.390511 -0.000461<br>C 3.016601 -0.170216 -0.000396<br>O 4.288821 0.231018 -0.000332<br>H -0.932366 -2.185116 0.000169<br>H 4.844873 -0.568435 -0.000327<br>SCF energy (Eh) = -957.168920 (M=3)
Pd_3Zn_3	Pd 1.131734319 -1.580367127 0.183934532<br>Pd -0.100307081 0.983653460 -0.158346994<br>Pd -1.679030323 -1.032210320 0.352872085<br>Zn 0.043293161 -0.269652215 2.027366186<br>Zn -2.595475425 1.297921234 0.005871245<br>Zn -0.316647324 -0.956256075 -1.767785332<br>O 1.799757516 2.433305554 -0.513818516<br>C 2.788339860 1.844762629 -0.335744051<br>O 3.786471877 1.296677676 -0.164984321<br>H 2.557320935 -2.546679825 -0.199985719<br>H 2.650190884 -2.382252490 0.602077685<br>SCF energy (Eh) = -5910.983931 (M=1)	Pd 1.178284 -1.761575 -0.496976<br>Pd -0.050442 0.483636 0.232215<br>Pd -2.596328 -0.366847 -0.940106<br>Zn -1.077827 -1.755209 0.548717<br>Zn -2.268629 1.382852 0.760929<br>Zn -0.405339 -0.502178 -2.114429<br>O 2.268065 2.063200 0.062651<br>C 3.077569 1.233948 0.014721<br>O 3.900886 0.425478 -0.036737<br>H -2.440944 -2.546393 0.642227<br>H 0.821867 -0.901255 0.968321<br>SCF energy (Eh) = -5910.964680 (M=1)	Pd 0.117317 -1.986044 -0.205063<br>Pd -0.183021 0.717317 0.247857<br>Pd -2.460024 -0.806534 -0.265755<br>Zn -1.010596 -1.062481 1.799754<br>Zn -2.438098 1.664414 0.150882<br>Zn -0.608285 -0.388770 -1.956869<br>O 2.104602 1.755598 0.504656<br>C 2.980883 1.129715 0.064905<br>O 3.865025 0.535532 -0.372067<br>H -1.608336 -2.314430 -0.499610<br>H 1.625455 -1.538644 0.102207<br>SCF energy (Eh) = -5910.966010 (M=1)
	Pd 0.389143 -1.674950 -0.191678<br>Pd -0.397589 1.017909 0.152874<br>Pd -2.320606 -0.870272 -0.244296<br>Zn -0.814212 -0.816331 1.814980<br>Zn -2.770436 1.566243 0.162268<br>Zn -0.596510 -0.241732 -1.999577<br>O 1.889620 1.297227 0.452277<br>C 2.645885 0.455746 0.048211<br>O 3.705580 0.152989 -0.356044<br>H -1.216312 -2.224313 -0.371359<br>H 1.881717 -0.939297 0.089736<br>SCF energy (Eh) = -5910.951464 (M=1)	Pd 1.483439 -1.279226 0.092327<br>Pd -0.028505 1.123760 0.003409<br>Pd -1.335573 -1.265238 -0.029695<br>Zn 0.015370 -0.418768 1.955209<br>Zn -2.463256 0.980204 -0.031337<br>Zn 0.152059 -0.461908 -1.916535<br>O 1.926471 1.956845 0.072093<br>C 2.953497 1.244427 -0.059483<br>O 4.087190 1.489696 -0.358347<br>H 0.135271 -2.223688 0.129772<br>H 2.783802 0.062789 0.193135<br>SCF energy (Eh) = -5910.955702 (M=1)	Pd 0.658479 -1.336717 -0.288593<br>Pd -0.505197 1.328834 0.193506<br>Pd -2.141014 -0.866515 -0.273988<br>Zn -0.614208 -0.660248 1.756809<br>Zn -2.927289 1.449807 0.198916<br>Zn -0.527852 0.069459 -1.998533<br>O 1.750284 1.252484 0.345838<br>C 2.157414 0.125763 0.045093<br>O 3.364503 -0.246708 -0.084361<br>H -0.943478 -2.097776 -0.395667<br>H 2.440514 -1.219099 -0.303419<br>SCF energy (Eh) = -5910.895725 (M=1)
		Pd 1.551761 -0.870569 -0.043256<br>Pd -0.214371 1.332668 0.034069<br>Pd -1.231627 -1.245389 -0.030655<br>Zn 0.100666 -0.326220 1.914025<br>Zn -2.596098 0.855611 0.031376<br>Zn 0.073440 -0.214274 -1.941835<br>O 2.108271 1.963807 0.057495<br>C 2.647244 0.852008 0.017262<br>O 3.987657 0.821760 0.021257<br>H 0.320576 -2.103154 -0.079283<br>H 4.285598 1.749396 0.058278<br>SCF energy (Eh) = -5910.964251 (M=1)	

Table S27. Cartesian coordinates and SCF energies of structures in Pd_2Zn_2 reaction paths (Figure S7 upper panel).

Pd_2Zn_2	Pd 1.698627 0.880573 -0.340408<br>Pd -2.209688 -0.236127 0.105136<br>Zn -0.200129 -0.340356 -1.265048<br>Zn -0.328687 1.068637 1.145587<br>H 1.398214 -0.024725 -1.751873<br>H -1.790366 -0.884242 -1.420297<br>O 2.656530 -1.892723 1.471703<br>C 1.807109 -2.289691 0.795684<br>O 0.958815 -2.713005 0.128850<br>SCF energy (Eh) = -4003.833727 (M=1)	Zn 2.160342 1.028698 -0.140712<br>Pd -0.284645 1.339761 0.104483<br>Pd 1.333561 -1.287002 0.018837<br>Zn -1.057756 -0.975102 0.043913<br>C -3.436399 -0.000933 -0.114385<br>O -4.208015 0.868272 -0.128128<br>H -1.911161 0.745608 0.095574<br>O -3.138296 -1.173006 -0.168961<br>H -0.027660 -2.336953 0.198676<br>SCF energy (Eh) = -4003.809823 (M=1)	Zn -0.884885 2.032212 1.134907<br>Pd 0.818096 0.895137 -0.177633<br>Pd -1.984091 0.533424 -0.493542<br>Zn -0.434382 -1.319585 -0.542736<br>C 2.136712 -1.850591 0.038971<br>O 3.208368 -2.341218 0.233269<br>H 2.020668 -0.703234 0.154365<br>O 1.035236 -2.453487 -0.289045<br>H -1.996325 -0.997246 -1.210482<br>SCF energy (Eh) = -4003.831211(M=1)
	Zn -1.01985 1.98892 1.34878<br>Pd -0.05411 1.57628 -0.88984<br>Pd -2.24840 0.33357 -0.09491<br>Zn -0.20288 -0.93096 -0.59316<br>C 2.30348 -2.17343 0.10936<br>O 2.69728 -2.59670 1.16129<br>H 3.00408 -1.72733 -0.62723<br>O 1.06153 -2.21568 -0.31215<br>H -1.84456 -0.71299 -1.30734<br>SCF energy (Eh) = -4003.828751 (M=1)	Zn 1.895276 1.233663 -0.000910<br>Pd -0.534354 1.204407 0.001672<br>Pd 1.403831 -1.161053 0.004389<br>Zn -1.001905 -1.230171 0.004726<br>C -3.351396 0.116167 0.005241<br>O -2.677404 1.153064 0.003950<br>H -4.447145 0.218730 0.006114<br>O -2.924495 -1.080856 0.005780<br>H 0.226015 -2.416907 0.004651<br>SCF energy (Eh) = -4003.860748 (M=1)	

Table S28. Cartesian coordinates and SCF energies of structures in Pd_2Zn_2 reaction paths (Figure S7 lower panel).

Pd_2Zn2_2	Pd 1.698627 0.880573 -0.340408<br>Pd -2.209688 -0.236127 0.105136<br>Zn -0.200129 -0.340356 -1.265048<br>Zn -0.328687 1.068637 1.145587<br>H 1.398214 -0.024725 -1.751873<br>H -1.790366 -0.884242 -1.420297<br>O 2.656530 -1.892723 1.471703<br>C 1.807109 -2.289691 0.795684<br>O 0.958815 -2.713005 0.128850<br>SCF energy (Eh) = -4003.833727 (M=1)	Zn -0.408568 -1.553803 -0.211776<br>Pd -2.284977 0.102718 -0.183123<br>Pd 1.784210 -0.290795 0.553381<br>Zn -0.240197 1.140484 0.632247<br>H -1.837608 1.638404 0.410626<br>O 1.127970 1.648141 -1.398174<br>C 1.916033 0.759219 -1.237962<br>O 2.831774 0.097593 -1.632317<br>H 1.161730 0.891515 1.615108<br>SCF energy (Eh) = -4003.828141 (M=1)	Zn -0.429723 -1.558328 -0.200063<br>Pd -2.305621 0.096903 -0.129723<br>Pd 1.782449 -0.336662 0.560237<br>Zn -0.200011 1.178296 0.498187<br>H -1.818674 1.643551 0.391471<br>O 1.085146 1.612441 -1.330281<br>C 1.916822 0.742593 -1.180242<br>O 2.862211 0.171592 -1.636961<br>H 1.157765 0.883094 1.575384<br>SCF energy (Eh) = -4003.828274 (M=1)
	Zn -1.033800 -1.687820 -0.622232<br>Pd -1.753303 0.534980 0.094761<br>Pd 0.946056 -0.613302 0.794671<br>Zn 0.399494 1.623094 -0.165052<br>H -1.178722 2.177080 -0.041302<br>O 2.092206 1.214068 -0.980210<br>C 2.307324 0.013783 -0.586060<br>O 3.083171 -0.814684 -0.940578<br>H 2.094315 0.089715 1.628573<br>SCF energy (Eh) = -4003.812932 (M=1)	Zn -0.822222 -1.442730 -1.345642<br>Pd -1.621585 0.438963 -0.009109<br>Pd 0.983127 -0.850424 0.506802<br>Zn 0.506289 1.590492 0.229054<br>H -1.089529 2.025519 0.495536<br>O 2.228213 1.393811 -0.576348<br>C 2.388230 0.116724 -0.608679<br>O 3.136065 -0.559620 -1.238686<br>H 2.250303 -0.642198 1.427657<br>SCF energy (Eh) = -4003.812983 (M=1)	Zn -1.199044 -1.578173 -0.715849<br>Pd -1.783724 0.598732 0.256231<br>Pd 0.990238 -0.829385 0.593246<br>Zn 0.364475 1.562773 -0.221814<br>H -1.181912 2.186892 0.153969<br>O 2.200133 1.430162 -0.757416<br>C 2.407176 0.247806 -0.318662<br>O 3.506567 -0.355518 -0.424555<br>H 2.622719 -1.198827 0.267721<br>SCF energy (Eh) = -4003.769188 (M=1)
	Zn 1.653087 -1.520835 -0.028421<br>Pd 1.682043 0.928570 0.128196<br>Pd -0.850759 -1.045770 -0.048931<br>Zn -0.657660 1.426138 -0.046871<br>H 0.749294 2.387193 -0.062066<br>O -2.614129 1.273483 -0.034583<br>C -2.589917 -0.000817 -0.025804<br>O -3.781007 -0.572134 0.002324<br>H -4.450272 0.138540 0.013321<br>SCF energy (Eh) = -4003.827294 (M=1)		
